# A Phase I, Randomized, Double-Blind, Placebo-Controlled, Single Ascending Dose, Multiple Ascending Dose and Food Effect Study to Evaluate the Tolerance, Pharmacokinetics of Jaktinib, a New Selective Janus Kinase Inhibitor in Healthy Chinese Volunteers

**DOI:** 10.3389/fphar.2020.604314

**Published:** 2020-12-14

**Authors:** Jingrui Liu, Binhua Lv, Hewen Yin, Xiaoxue Zhu, Haijing Wei, Yanhua Ding

**Affiliations:** ^1^ Phase I Clinical Trial Unit, The First Hospital of Jilin University, Changchun, China; ^2^ Suzhou Zelgen Biopharmaceuticals Co., Ltd., Jiangsu, China

**Keywords:** jaktinib, pharmacokinetics, food effect, safety, phase I

## Abstract

**Background:** Jaktinib is a novel selective janus kinase 1/2 inhibitor. The phase I first-in-human study evaluated the tolerance and pharmacokinetics of jaktinib in healthy Chinese subjects.

**Methods:** A randomized, double-blind, placebo-controlled study were designed. A total of 126 healthy subjects were enrolled into the single ascending dose, multiple ascending dose and food effect study. Safety endpoints included adverse events, abnormal vital signs, 12-lead ECGs, abdominal ultrasound, chest x-ray, physical examination and clinical laboratory tests. Blood, urine and feces samples were collected at predetermined time points for pharmacokinetic analysis of jaktinib, the metabolites ZG0244 and ZG0245, which are formed by oxidation or hydrolysis metabolic pathway, respectively.

**Results:** Jaktinib was absorbed with a median time to peak plasma concentration of 1.25–3.5 h and was eliminated with a half-life of 2.952–9.040 h. Linear pharmacokinetic characteristic was presented over the dose range from 25 to 400 mg. No obvious accumulation was observed after multiple doses for 10 days. Administration after a high-fat breakfast significantly increased the absorption of jaktinib. The accumulated fraction of jaktinib and the determined metabolites excreted in urine and feces was 19.478%. Jaktinib was well tolerated in all single dose cohorts. In multiple dose cohorts, 200 mg q24 h method was evaluated as maximally tolerated dose. Neutropenia, diarrhea, dizziness and headache were the most frequently reported treatment related adverse events. No deaths, serious or Grade ≥4 adverse events was developed.

**Conclusion:** Jaktinib was well tolerated when single dose ranging from 25 to 400 mg and multiple dose up to 200 mg q24 h. The safety and pharmacokinetic characteristics support the next trial in myelofibrosis patients.

## Introduction

Myelofibrosis (MF), a category of chronic myeloproliferative neoplasms (MPN) that can present a *de novo* disease known as primary MF (PMF) or develop from other types of MPN including polycythemia vera (PV) and essential thrombocythemia (ET) (Mesa et al., 2016; Tefferi, 2016; Iurlo and Cattaneo, 2017; Rumi and Cazzola, 2017). It is a rare disease with clonal proliferation of pluripotent hematopoietic stem cells. The typical clinical characteristics of MF are anemia, splenomegaly, thrombocytosis, bone marrow fibrosis and constitutional symptoms (weight loss, fever, fatigue and cachexia) (Pardanani et al., 2011; Harrison et al., 2018). The age range of disease onset in most patients is 50–70 years old, with median survival in the range of 4–7 years (Barosi, 2003; Cervantes et al., 2009; Xu et al., 2019). The conventional therapies for MF, such as splenectomy and radiotherapy, have limited impact on the patients’ survival and often used for palliative treatment (Abdel-Wahab and Levine, 2009; Verstovsek et al., 2012). Allogeneic hematopoietic stem cell transplantation (allo-HSCT) is considered as the only curative treatment for patients with MF. However, a significant proportion of patients are transplant ineligible, due to poor performance status and advanced age at the time of diagnosis.

In 2005, the relationship between Janus kinase (JAK) mutations and MF was unveiled. The JAK2 V617 F mutation has been identified in 50–60% of patients with MF(Levine et al., 2007; Delhommeau et al., 2010; Klampfl et al., 2013). Ruxolitinib, a small-molecule selective JAK1/2 inhibitor, has been approved as the first JAK inhibitor for the treatment of MF in 2011. Two pivotal phase three clinical trials demonstrated the efficacy of ruxolitinib in the significant reduction of spleen volume, improvement on MF-related symptoms and prolonged overall survival (OS) (Verstovsek et al., 2012; Verstovsek et al., 2017; Al-Ali et al., 2020). However, the dose-related adverse reactions, including anemia, thrombocytopenia and infections were seen to be a major limitation in the use of ruxolitinib. Momelotinib, another selective small-molecule JAK1/2 inhibitor, has gained U.S. FDA Fast Track designation for the treatment of patients within intermediate/high-risk MF who previously used ruxolitinib in 2019. Several clinical studies showed that momelotinib was noninferior to ruxolitinib in spleen response but significantly improved anemia response (Pardanani et al., 2013; Gupta et al., 2017; Mesa et al., 2017; Harrison et al., 2018).

Jaktinib is a newly developed selective JAK1/2 inhibitor with 50% inhibitory concentration (IC50) of 0.1 μM on cells transformed by selected oncogenic mutants of tyrosine kinases. It has the similar inhibition potency to momelotinib and ruxolitinib. Preclinical toxicology studies were conducted in both Wistar rats and Beagle dogs with oral administration of jaktinib. The no observed adverse effect level (NOAEL) were 18.3 mg/kg/day and 30 mg/kg/day in Beagle dogs and Wistar rats, respectively. Based on the NOAEL dose and a 10-fold safety factor, the recommended phase I starting dose was 25 mg/day. This was a first-in-human study to investigate the tolerance, safety, pharmacokinetics and food effect of jaktinib in healthy Chinese volunteers.

## Materials and Methods

### Healthy Volunteers

Eligible volunteers were healthy male and female Chinese aged 18–45 years with body mass index of 18–28 kg/m^2^, and a total body weight of male and female subjects was no less than 50 and 45 kg, respectively. Other eligibility criteria included no history of cardiac, hepatic, renal, gastrointestinal, or neurologic diseases. All subjects were required to practice birth control and have no plan to conceive during the next 6 months.

Exclusion criteria included smoking more than five cigarettes per day within the past 3 months; any clinically significant laboratory test or 12-lead ECG abnormality; history of drug abuse and/or alcoholism; intake of any other drugs, vitamins, or herbal medicine within 14 days or intake of any drugs known to influence the activity of drug metabolizing enzymes (CYP3A4, 1A2, 2D6 and 2C9) within 28 days before the first dose of the trial medication; pregnant or breastfeeding; multiple food and drug allergies.

The study was approved by the Ethics Committee of the First Hospital of Jilin University, and conducted in accordance with the ethical principles originating in the Declaration of Helsinki version 2013 and in compliance with International Conference on Harmonization Good Clinical Practice Guidelines. All volunteers provided written informed consent.

### Study Design

This was a phase I, randomized, double-blind, placebo-controlled, dose-escalation study conducted at First Hospital of Jilin University, China (ClinicalTrials.gov identifier: NCT03314402) from October 2017 to March 2018. Jaktinib hydrochloride 50 mg film-coated tablets were produced and supplied by Suzhou Zelgen Biopharmaceuticals Co., Ltd., China. The study comprised three parts: single ascending dose (SAD), multiple ascending dose (MAD) and food effect.

In SAD part, volunteers were designed into eight SAD cohorts (25, 50, 100, 150, 200, 250, 300 and 400 mg). Among these cohorts, 25 mg was bisected by the 50 mg specification under good manufacturing practices (GMP) condition and provided the certificate of analysis. In each cohort, eight healthy volunteers were randomized (3:1) to receive jaktinib or placebo with a similar proportion of male and female. Jaktinib or placebo was administrated after an overnight fast.

MAD study was conducted after evaluating the tolerance of the single dose 100 mg cohort. In MAD part, volunteers were designed into five cohorts (100 mg q24h, 150 mg q24h, 100 mg q12h, 200 mg q24h, 150 mg q12h). In each cohort, 10 volunteers were randomized (8:2) to receive jaktinib or placebo in a close proportion of male and female and jakitinib or placebo was administrated for 10 days under fasting condition ranging from 100 mg q24h to 150 mg q12h. The MAD part was initiated after the safety and tolerance evaluation from SAD 100 mg cohorts. Progression to higher MAD doses was allowed after review the safety data of the prior MAD cohorts.

Food effect part was a two-period crossover study and conducted after finishing both SAD and MAD parts. 12 volunteers were randomized (1:1) with a similar proportion of male and female to receive their first dose of jaktinib 200 mg under fasted (group A) or fed (group B) conditions (period 1). After a washout period of 5 days, volunteers received their second dose of jaktinib 200 mg under the other condition (fed in group A and fasted in group B, period 2). For group A, the urine and feces samples for pharmacokinetic (PK) analysis were collected in the first period.

Dose-limiting toxicities (DLTs) were defined according to the NCI CTCAE v.4.03 as any occurrence of the following drug-related adverse events (AEs): more than one third of volunteers suffered Grade ≥4 drug-related hematological toxicities or Grade ≥3 drug-related nonhematological toxicities; one drug-related serious adverse events (SAEs).

The primary objectives of the study were to evaluate the safety and tolerance, determine the DLT and maximal tolerated dose (MTD) of jaktinib. The secondary objectives were to explore the PK profile and the food effect on PK profile of jaktinib.

### Pharmacokinetics

Blood samples (4 ml) for pharmacokinetic analysis of jaktinib, main metabolites ZG0244 (similar JAK1/2 inhibitory activity to jaktinib) and ZG0245 (no JAK1/2 inhibitory activity) were collected in tubes containing K_2_EDTA anticoagulant at 0 (pre-dose), 0.25, 0.5, 1, 1.5, 2, 3, 4, 5, 6, 8, 12, 14, 24 and 36 h post-dosing in SAD cohorts. For MAD cohorts, blood samples were collected at 0, 0.25, 0.5, 1, 1.5, 2, 3, 4, 5, 6, 8 and 12 h on day 1; 0 h on day 2, 3, 4, 6 and 7; 0, 0.25, 0.5, 1, 1.5, 2, 3, 4, 5, 6, 8, 12, 24, 36 and 48 h on day 10. In food effect cohort, blood samples were collected at 0, 0.25, 0.5, 1, 1.5, 2, 3, 4, 5, 6, 8, 12, 24, 36, 48, 72, 96 and 120 h in period one of group A; 0, 0.25, 0.5, 1, 1.5, 2, 3, 4, 5, 6, 8, 12, 24, 36 and 48 h in period two of group A and the two periods of group B. Blood samples were centrifuged at 3,000 rpm for 10 min at 2–8 °C and stored at -80 °C until liquid chromatography-mass spectrophotometry (LC-MS/MS) analysis. Urine and feces samples were collected in period one of group A of food effect cohort with the intervals 0, 0–6, 6–12, 12–24, 24–48, 48–72, 72–96, 96–120 and 0–120 h, respectively.

Plasma pharmacokinetic data were analyzed by standard non-compartmental methods using WinNonlin version 7.0 (Certara USA Inc.) and the pharmacokinetic parameters included peak plasma concentration (C_max_), time to peak plasma concentration (T_max_), area under the plasma concentration–time curve from time 0 to 12 or 24 h (AUC_0-12_, AUC_0-24_), AUC from time 0 to the last timepoint with a quantifiable concentration (AUC_last_), AUC from time 0 to infinity (AUC_0-∞_), terminal elimination half-life (t_1/2_), clearance (CL/F), apparent volume of distribution (V_z_/F) and the parameters at steady state in MAD cohorts: C_,max,ss_, C_min,ss_, T_max,ss_, AUC_0-24,ss_, AUC_last,ss_, AUC_0-∞,ss_, CL/F_ss_ and V_z_/F_ss_. Urine and feces PK parameter was accumulated excretion recovery ratio from time zero to 120 h (Fe_0–120h_).

### Safety Analysis

Safety was assessed according to the NCI CTCAE version 4.03, including AEs of changes in vital signs, 12-lead ECGs, abdominal ultrasound, chest X-ray, physical examination and clinical laboratory tests. Abdominal ultrasound and chest X-ray were only performed at screening. 12-lead ECGs, physical examination and clinical laboratory tests were conducted in screening and day 3 after administration in SAD cohorts; days 4, 7 and 12 in MAD cohorts; day 8 in food effect cohort. Vital signs were measured at screening and admission, 0 h (pre-dose) and 2, 6, 24, 48 h after administration in SAD cohorts; 0, 2, 6 h on day 1, 0 h on days 2, 3, 4, 7, 0, 2, 6, 24, 48 h after the last dose in MAD cohorts; 0, 2, 6, 24, 48, 72, 120 h in period 1 and 2, 6, 24, 48 h in period two in food effect cohort.

### Concomitant Medications

No concomitant medications were allowed during the study except for the treatments of AEs. Potent inhibitors and inducers of hepatic metabolic enzymes were prohibited from 4 weeks before administration to throughout the study and closely monitoring should be given when combining CYP2C9 substrates with narrow range of treatment.

### Statistical Analysis

Statistical analysis was performed using SAS software, version 9.4 (SAS Institute Inc., United States). Descriptive statistics were used to summarize continuous variables as cases, means with standard deviations, medians, maximum and minimum. For categorical variables, frequencies and percentages were used. A regression power model, relating log-transformed C_max_ and AUC parameters to log-transformed dose, was used to investigate dose proportionality. Food effect pharmacokinetic parameters were analyzed using the mixed effect model with period, sequence and treatment as fixed effects and subjects as the random effect. Logarithmic transformation of C_max_, AUC_0-∞_ and AUC_last_ were performed to calculate the 90% confidence intervals (CIs) of the geometric mean ratio (GMR) between fasting and fed administration. If the calculated 90% CI values fall entirely within the range of 0.80–1.25, it is indicative of no significant food effect.

## Results

### Demographics and Baseline Data

A total of 126 healthy volunteers were enrolled into the study; 64, 50 and 12 volunteers enrolled in the SAD, MAD and food effect part, respectively. The baseline demographic characteristics of the enrolled cases are presented in Table 1.

**TABLE 1:** Summary of baseline demographic characteristics

jaktinib dose cohorts	Gender, n (%)		Race, n (%)		Age, years [mean (SD)]	BMI, kg/m^2^ [mean (SD)]
	Male	Female	Asian	Others		
Single ascending dose cohorts						
25 mg (n = 6)	3 (50%)	3 (50%)	6 (100%)	0	32.8 (9.22)	24.0 (2.47)
50 mg (n = 6)	3 (50%)	3 (50%)	6 (100%)	0	30.5 (6.80)	23.4 (2.48)
100 mg (n = 6)	3 (50%)	3 (50%)	6 (100%)	0	33.7 (4.55)	22.5 (2.19)
150 mg (n = 6)	3 (50%)	3 (50%)	6 (100%)	0	30.2 (6.08)	23.0 (1.95)
200 mg (n = 6)	3 (50%)	3 (50%)	6 (100%)	0	32.3 (5.65)	23.0 (1.94)
250 mg (n = 6)	3 (50%)	3 (50%)	6 (100%)	0	32.3 (6.44)	21.8 (1.33)
300 mg (n = 6)	3 (50%)	3 (50%)	6 (100%)	0	36.2 (5.88)	24.9 (1.32)
400 mg (n = 6)	3 (50%)	3 (50%)	6 (100%)	0	29.5 (6.98)	24.1 (2.87)
Multiple ascending dose cohorts						
100 mg q24h (n = 8)	4 (100%)	4 (100%)	8 (100%)	0	29.5 (8.94)	22.8 (2.22)
150 mg q24h (n = 8)	4 (100%)	4 (100%)	8 (100%)	0	30.8 (6.36)	23.2 (2.76)
100 mg q12h (n = 8)	4 (100%)	4 (100%)	8 (100%)	0	29.1 (7.59)	21.6 (2.36)
200 mg q24h (n = 8)	4 (100%)	4 (100%)	8 (100%)	0	28.8 (5.52)	22.2 (1.86)
150 mg q12h (n = 8)	4 (100%)	4 (100%)	8 (100%)	0	32.9 (6.10)	22.7 (2.19)
Placebo (n = 10)	6 (60%)	4 (40%)	10 (100%)	0	31.0 (5.91)	22.4 (2.31)
Food effect cohort						
Group A (fasted-fed) (n = 6)	3 (50%)	3 (50%)	6 (100%)	0	32.2 (7.63)	22.6 (2.50)
Group B (fed-fasted) (n = 6)	3 (50%)	3 (50%)	6 (100%)	0	35.3 (2.94)	23.7 (1.84)

N, number of subjects; SD, standard deviation; BMI, body mass index.

### Pharmacokinetic Properties

**FIGURE 1:**
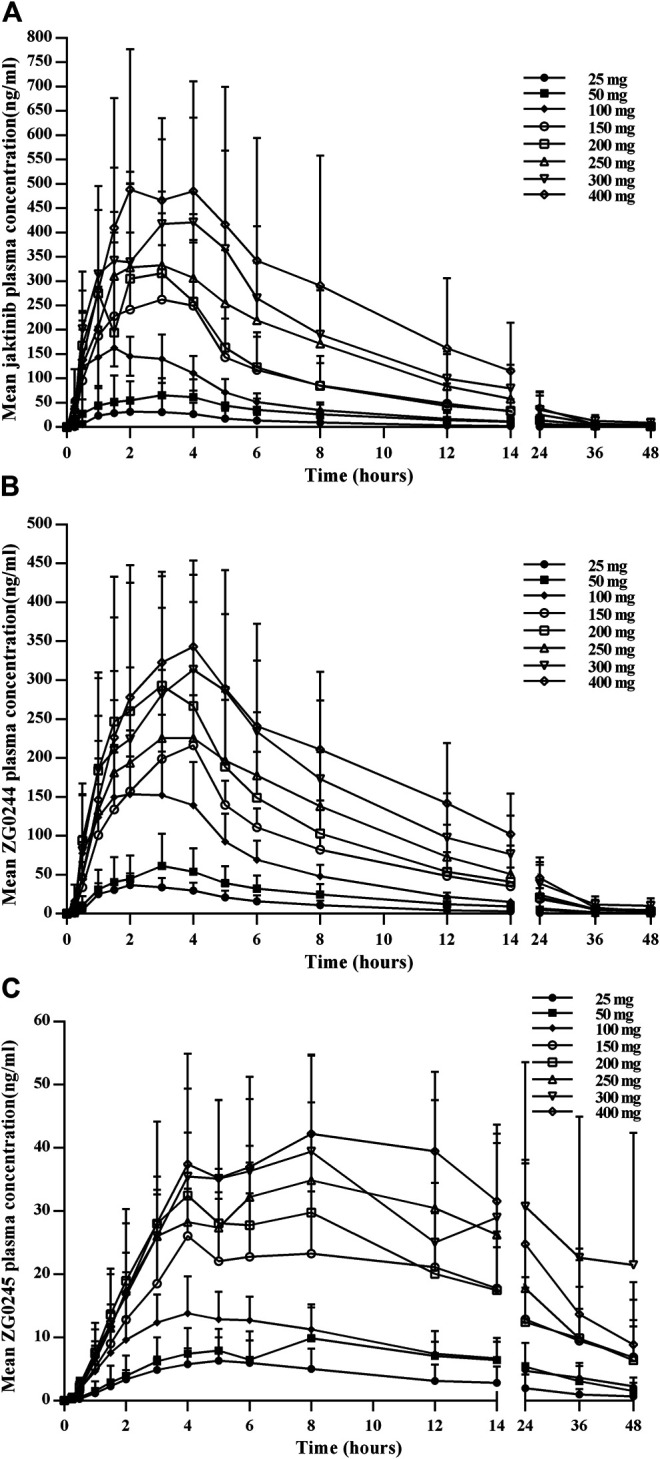
Mean plasma concentration-time profiles for jaktinib **(A)**, ZG0244 **(B)** and ZG0245 **(C)** after single ascending dose 25–400 mg jaktinib.

The mean jaktinib, metabolites ZG0244 and ZG0245 plasma concentration-time profiles after SAD are shown in Figure 1. After single dose of oral administration, jaktinib was rapidly absorbed with the median T_max_ 1.25–3.5 h, which is independent of dose. The t_1/2_ were 2.952–9.040 h and didn't show a proportional increase with increasing dosages of administration. The jaktinib AUC_0-∞_ and C_max_ increased dependent on dose, the increase trend was basically in line with the dose proportionality. The correlative analysis of dosages and pharmacokinetic properties were analyzed using WinNonlin linear mixed effects model and the slopes (90% CI) were 1.15 (0.96–1.34) and 1.00 (0.86–1.15) for ln AUC_0-∞_ and ln C_max_, close to 1, suggesting a clear linear pharmacokinetic characteristic within the dose range of jaktinib 25–400 mg. The CL/F was 76.212–150.190 L/h with a large V_z_/F 533.561–1033.554 L, suggesting a wide distribution in the body. For metabolites ZG0244 and ZG0245, the T_max_ were 2–4 and 4–8 h with the t_1/2_ 3.042–8.511 and 8.08–24.549 h, respectively. The metabolic rate were 0.789–1.325 and 0.279–0.584 (calculating by AUC_0-∞_), decreased as the dose increasing. Table 2 summarizes the plasma PK parameters of jaktinib and metabolites ZG0244, ZG0245 after single dose of jaktinib 25–400 mg.

**TABLE 2:** Pharmacokinetic (PK) properties of jaktinib, ZG0244 and ZG0245 after single ascending dose of 25, 50, 100, 150, 200, 250, 300 and 400 mg jaktinib.

PK parameter	Dosage							
	25 mg (n = 6)	50 mg (n = 6)	100 mg (n = 6)	150 mg (n = 6)	200 mg (n = 6)	250 mg (n = 6)	300 mg (n = 6)	400 mg (n = 6)
Jaktinib								
T_max_, median (min–max), h	2.00 (1.00–4.00)	3.00 (0.50–5.00)	1.25 (0.50–3.00)	2.50 (1.50–4.00)	2.50 (1.00–3.00)	2.00 (1.00–4.00)	3.00 (0.50–5.00)	3.50 (2.00–5.00)
C_max_, geometric mean (CV%), ng/ml	34.50 (51.6)	77.56 (72.8)	183.48 (33.3)	297.18 (36.6)	354.52 (44.6)	404.20 (36.9)	510.54 (35.4)	579.35 (42.6)
AUC_0-t_, geometric (CV%), h·ng/ml	154.157 (84.7)	461.588 (93.6)	958.347 (25.4)	1806.831 (52.8)	2060.864 (46.5)	2680.222 (69.4)	3759.837 (49.6)	4260.961 (66.4)
AUC_0-∞_, geometric (CV%), h·ng/ml	166.455 (86.5)	501.141 (83.8)	992.089 (23.9)	1892.862 (54.3)	2132.988 (44.0)	2750.269 (71.1)	3936.399 (50.4)	4314.433 (65.7)
T_1/2_, geometric (CV%), h	2.952 (79.2)	5.490 (61.1)	6.283 (92.2)	9.040 (68.3)	6.328 (76.8)	4.069 (67.5)	8.548 (72.4)	5.561 (18.5)
V_z_/F, geometric (CV%), L	639.741 (75.8)	790.229 (69.5)	913.730 (117.3)	1033.554 (104.6)	856.012 (96.5)	533.561 (30.0)	939.818 (78.4)	743.770 (75.1)
CL/F, geometric (CV%), L/h	150.190 (86.5)	99.772 (83.8)	100.797 (23.9)	79.245 (54.3)	93.765 (44.0)	90.900 (71.1)	76.212 (50.4)	92.712 (65.7)
ZG0244								
T_max_, median (min–max), h	2.00 (1.00–3.00)	3.00 (1.00–4.00)	2.50 (1.50–3.00)	3.50 (2.00–4.00)	3.00 (1.50–4.00)	2.50 (1.50–4.00)	4.00 (3.00–5.00)	4.00 (2.00–5.00)
C_max_, geometric mean (CV%), ng/ml	40.63 (32.9)	57.12 (81.5)	156.52 (36.6)	215.67 (31.7)	300.64 (40.9)	235.26 (49.2)	311.42 (52.8)	345.40 (36.5)
AUC_0-t_, geometric (CV%), h·ng/ml	214.964 (34.1)	446.142 (56.4)	1120.171 (23.8)	1813.743 (15.3)	2254.686 (34.6)	2153.077 (64.0)	3133.898 (51.3)	3652.851 (39.9)
AUC_0-∞_, geometric (CV%), h·ng/ml	226.862 (35.3)	459.053 (56.4)	1162.042 (20.4)	1901.628 (15.0)	2347.444 (33.1)	2232.624 (66.1)	3297.937 (51.4)	3681.322 (40.3)
T_1/2_, geometric (CV%), h	3.042 (63.2)	5.862 (79.4)	7.192 (52.2)	8.481 (63.3)	6.460 (69.9)	5.047 (64.2)	8.511 (52.2)	5.467 (28.9)
MR (AUC_0-∞_), geometric (CV%)	1.325 (46.8)	0.891 (74.1)	1.139 (30.7)	0.977 (62.6)	1.070 (29.9)	0.789 (50.7)	0.814 (54.9)	0.830 (32.0)
ZG0245								
T_max_, median (min–max), h	4.50 (4.00–6.00)	8.00 (3.00–24.00)	5.00 (3.00–6.00)	6.00 (4.00–12.00)	4.00 (3.00–8.00)	8.00 (4.00–24.00)	6.50 (4.00–24.00)	8.00 (4.00–12.00)
C_max_, geometric mean (CV%), ng/ml	6.113 (58.2)	9.933 (44.1)	13.946 (35.4)	24.749 (49.1)	33.174 (33.8)	36.861 (38.3)	43.117 (52.5)	44.167 (39.0)
AUC_0-t_, geometric (CV%), h·ng/ml	78.045 (102.9)	203.123 (64.5)	268.607 (21.8)	569.258 (68.9)	641.070 (49.7)	683.440 (77.3)	1119.091 (68.1)	1017.297 (43.4)
AUC_0-∞_, geometric (CV%), h·ng/ml	88.393 (102.8)	236.560 (79.7)	353.382 (32.2)	878.757 (94.9)	871.916 (95.3)	572.198 (56.5)	1519.338 (91.5)	1264.210 (59.6)
T_1/2_, geometric (CV%), h	9.520 (38.9)	14.167 (48.3)	18.249 (76.6)	24.549 (99.8)	19.125 (103.0)	8.080 (37.8)	24.321 (89.0)	15.876 (71.4)
MR (AUC_0-∞_), geometric (CV%)	0.584 (46.8)	0.475 (28.8)	0.392 (51.5)	0.511 (69.1)	0.450 (94.9)	0.279 (14.7)	0.428 (53.2)	0.322 (59.6)

T_max_, time to peak plasma concentration; C_max_, peak plasma concentration; AUC_0-t_, area under the plasma concentration-time curve from time zero to time t; AUC_0-∞_, area under the plasma concentration-time curve from time zero to infinity; T_1/2_, terminal elimination half-life; V_z_/F, apparent volume of distribution; CL/F, apparent clearance; MR, metabolic rate; CV%, percentage coefficient of variation.

**FIGURE 2:**
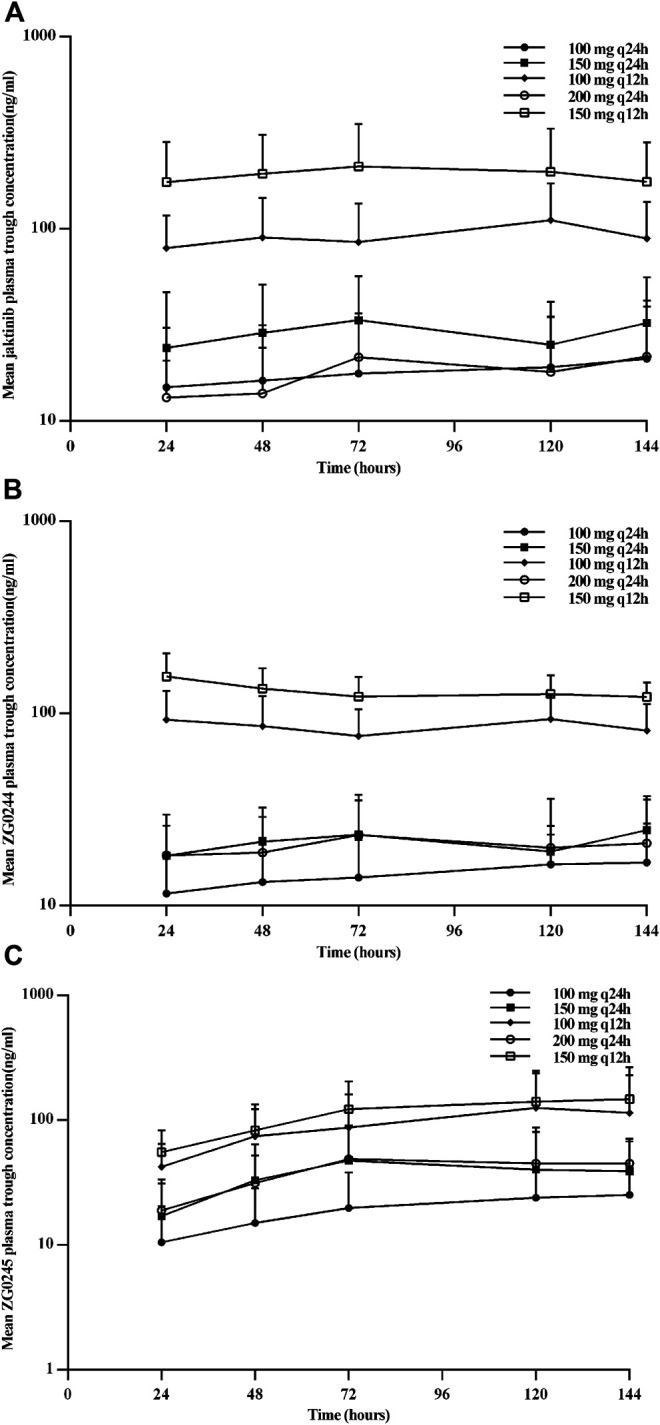
Mean plasma trough concentration-time profiles for jaktinib **(A)**, ZG0244 **(B)** and ZG0245 **(C)** after multiple ascending dose.

**FIGURE 3:**
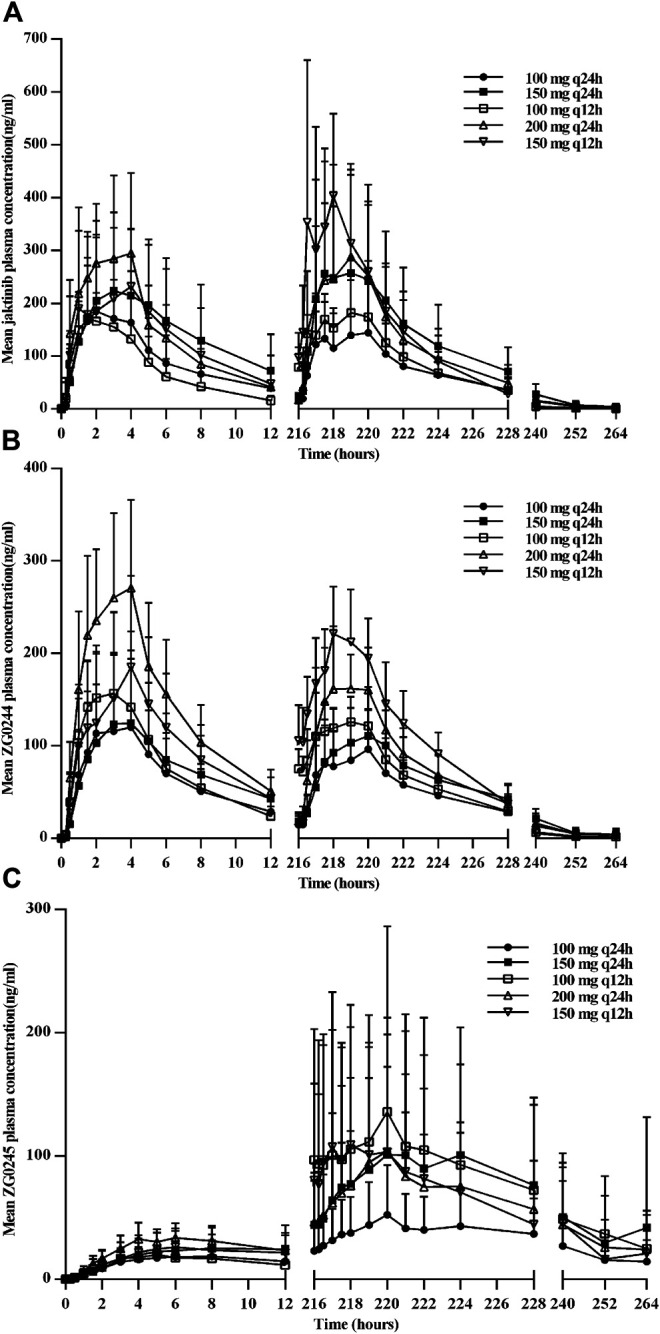
Mean plasma concentration-time profiles for jaktinib **(A)**, ZG0244 **(B)** and ZG0245 **(C)** after multiple ascending dose.

Figure 2 shows the mean jaktinib, metabolites ZG0244 and ZG0245plasma trough concentration-time profiles after MAD and Figure 3 shows the mean jaktinib, metabolites ZG0244 and ZG0245 plasma concentration-time profiles after MAD. Visual inspection of the jaktinib trough concentration data indicated that steady-state were reached after approximately 4 days continuous administration. The extent of jaktinib accumulation were analyzed using the mixed effect model with treatment and days as fixed effects and subjects as the random effect. For q24h treatment groups, the accumulation factor R_ac_ was calculated using AUC_0-24_ and AUC_0-24,ss_, and for q12h, it was AUC_0-12_ and AUC_0-12,ss_. The R_ac_ were 0.98 (90% CI:0.53, 1.81), 1.16 (90% CI:0.58, 2.31), 1.24 (90% CI:0.85, 1.80), 0.94 (90% CI:0.55, 1.62), 1.38 (90% CI:0.72, 2.65) for 100 mg q24h, 150 mg q24h, 100 mg q12h, 200 mg q24h and 150 mg q12h cohorts, respectively, showing no obvious accumulation. For metabolites ZG0244 and ZG0245, the R_ac_ was 0.668–1.150 and 2.252–5.286, respectively. No accumulation was observed for ZG0244 and there was moderate accumulation for ZG0245. Table 3 summarizes the plasma PK parameters of jaktinib and metabolites ZG0244, ZG0245 after multiple doses of jaktinib.

**TABLE 3:** Pharmacokinetic (PK) properties of jaktinib, ZG0244 and ZG0245 after multiple ascending dose of 100 mg q24h, 150 mg q24h, 100 mg q12h, 200 mg q24h, 150 mg q12h jaktinib.

PK parameter	Dosage									
	100 mg q24h (n = 8)		150 mg q24h (n = 8)		100 mg q12h (n = 7)		200 mg q24h (n = 8)		150 mg q12h (n = 4)	
	Day 1	Day 10	Day 1	Day 10	Day 1	Day 10	Day 1	Day 10	Day 1	Day 10
Jaktinib										
T_max_, median (min–max), h	2.00 (1.00–4.00)	1.50 (1.00–4.00)	3.00 (1.00–6.00)	2.50 (1.00–5.00)	1.50 (0.50–4.00)	3.00 (0.50–4.00)	2.50 (1.50–4.00)	3.00 (1.50–4.00)	2.00 (1.00–4.00)	1.25 (0.50–2.00)
C_max_, geometric mean (CV%), ng/ml	182.68 (73.4)	150.84 (76.7)	236.94 (88.6)	250.23 (70.3)	204.68 (36.2)	226.87 (33.2)	327.37 (36.6)	296.88 (58.3)	295.08 (52.6)	470.78 (38.2)
C_min_,ss, geometric mean (CV%), ng/ml	—	8.217 (169.4)	—	14.342 (71.1)	—	27.565 (96.3)	—	10.798 (125.4)	—	26.868 (39.2)
AUC_0–12h_, geometric (CV%), h·ng/ml	939.123 (81.3)	871.739 (66.7)	1368.274 (102.3)	1532.046 (78.1)	924.607 (37.1)	1143.528 (44.3)	1597.144 (49.3)	1494.683 (74.4)	1405.691 (57.5)	1940.162 (42.4)
AUC_0–24h_, geometric (CV%), h·ng/ml	1142.089 (88.1)	1122.248 (68.1)	1726.048 (107.0)	1995.775 (77.5)	—	1296.821 (49.2)	1848.272 (51.8)	1742.556 (83.1)	—	2072.558 (40.8)
R_ac_, geometric (CV%)	—	0.983 (31.1)	—	1.156 (58.2)	—	1.237 (30.4)	—	0.943 (51.8)	—	1.38 (17.8)
Fluctuation, geometric mean (CV%), %	—	290.146 (45.7)	—	281.461 (29.1)	—	204.463 (31.3)	—	390.098 (38.5)	—	274.265 (17.3)
ZG0244										
T_max_, median (min–max), h	3.00 (2.00–4.00)	2.00 (1.50–4.00)	3.00 (2.00–5.00)	3.50 (2.00–5.00)	2.00 (1.50–4.00)	3.00 (1.00–4.00)	3.50 (1.50–4.00)	3.00 (1.50–4.00)	3.00 (1.50–4.00)	2.00 (1.00–3.00)
C_max_, geometric mean (CV%), ng/ml	114.44 (58.5)	95.00 (45.4)	117.78 (64.5)	110.60 (39.7)	175.14 (36.8)	146.07 (17.5)	285.21 (34.6)	179.23 (32.4)	207.62 (48.0)	228.37 (20.9)
C_min_,ss, geometric mean (CV%), ng/ml	—	9.744 (70.2)	—	14.550 (41.7)	—	27.130 (42.3)	—	11.398 (125.9)	—	36.649 (26.0)
AUC_0–12h_, geometric (CV%), h·ng/ml	715.528 (53.7)	641.222 (37.7)	804.176 (60.4)	807.942 (36.2)	975.388 (33.9)	901.959 (10.9)	1643.934 (38.1)	1070.916 (39.5)	1277.760 (48.5)	1469.934 (22.5)
;AUC_0–24h_, geometric (CV%), h·ng/ml	928.944 (48.5)	877.104 (31.6)	1130.390 (51.0)	1163.018 (15.4)	—	1075.219 (15.4)	1994.285 (38.9)	1331.925 (48.1)	—	1661.926 (21.0)
R_ac_, geometric (CV%)	—	0.944 (26.7)	—	1.029 (30.3)	—	0.925 (30.3)	—	0.668 (32.3)	—	1.150 (29.8)
Fluctuation, geometric mean (CV%), %	—	220.955 (40.5)	—	195.447 (29.6)	—	154.060 (27.1)	—	294.474 (38.7)	—	156.227 (12.6)
ZG0245										
T_max_, median (min–max), h	5.50 (4.00–8.00)	4.00 (4.00–8.00)	10.00 (4.00–24.00)	5.00 (4.00–8.00)	5.00 (3.00–8.00)	3.00 (2.00–5.00)	4.50 (3.00–8.00)	4.00 (2.00–4.00)	4.50 (4.00–6.00)	3.00 (1.00–8.00)
C_max_, geometric mean (CV%), ng/ml	16.298 (80.3)	38.55 (120.4)	21.079 (110.0)	72.77 (129.4)	19.049 (31.4)	94.60 (118.8)	32.827 (43.7)	81.36 (103.9)	24.102 (62.1)	83.50 (112.1)
C_mi_n,ss, geometric mean (CV%), ng/ml	—	14.186 (153.7)	—	23.860 (135.3)	—	46.337 (136.9)	—	24.087 (230.6)	—	35.858 (98.9)
AUC_0–12h_, geometric (CV%), h·ng/ml	135.825 (88.0)	348.179 (118.9)	174.844 (110.4)	648.848 (140.3)	152.792 (31.1)	807.632 (118.7)	279.846 (44.7)	696.769 (102.0)	200.764 (70.2)	725.156 (103.5)
AUC_0–24h_, geometric (CV%), h·ng/ml	237.243 (103.6)	606.015 (127.9)	336.420 (129.4)	1117.500 (144.0)	—	1279.629 (125.0)	493.261 (57.2)	1111.008 (118.9)	—	1116.190 (112.1)
R_ac_, geometric (CV%)	—	2.544 (48.1)	—	3.322 (88.4)	—	5.286 (78.6)	—	2.252 (58.1)	—	3.612 (40.0)
Fluctuation, geometric mean (CV%), %	—	92.257 (32.5)	—	101.854 (33.4)	—	69.190 (31.9)	—	115.215 (32.2)	—	68.363 (74.0)

T_max_, time to peak plasma concentration; C_max_, peak plasma concentration; AUC_0–12h_, area under the plasma concentration-time curve from time zero to 12 h; AUC_0–24h_, area under the plasma concentration-time curve from time zero to 24 h; R_ac_, accumulation ratio; CV%, percentage coefficient of variation.

**FIGURE 4:**
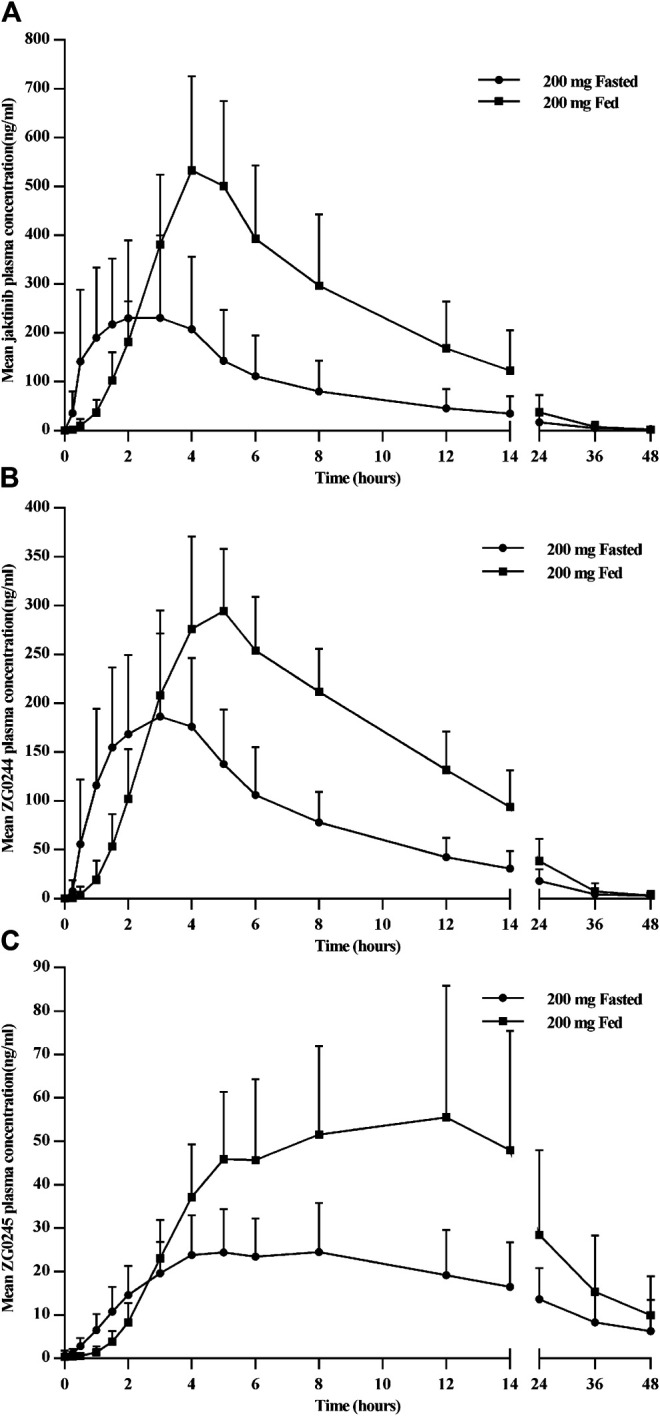
Mean plasma concentration-time profiles for jaktinib **(A)**, ZG0244 **(B)** and ZG0245 **(C)** for the fasted and fed state after single dose 200 mg jaktinib.

Figure 4 shows the mean jaktinib, ZG0244 and ZG0245 plasma concentration-time profiles in the fasted and fed state. Following a high-fat pre-dose meal, there was significant increase in jaktinib C_max_ of 2.6-fold and AUC_0-∞_ of 2.28-fold.The inter-individual variation of main PK parameters decreased in the fed state. The results of mixed effect model analysis showed no calculated 90% CI values of AUC_0-t_, AUC_0-∞_ and C_max_ fell entirely within the range of 0.80–1.25 (Table 4), it is indicative of significant food effect. The median T_max_ was significantly prolonged from 2 h in the fasted state to 4 h in the fed state (*p* < 0.05). The metabolites ZG0244 and ZG0245 showed a similar food effect PK characteristics to jaktinib. In the fed state, the C_max_ of 1.56-fold and AUC_0-∞_ of 1.93-fold was observed for ZG0244 and it was both 1.94-fold for ZG0245. The median T_max_ was extended by 2 and 1.5 h, respectively. Table 5 summarizes the plasma PK parameters of jaktinib and metabolites ZG0244, ZG0245 under the fasted and fed conditions.

**TABLE 4:** Geometric mean ratios of pharmacokinetic (PK) parameters in the fasted and fed state.

PK parameter	Geometric mean		Geometric mean ratio	
	Fasted (n = 12)	Fed (n = 12)	Ratio	90% Cl
AUC_0-t_ (h·ng/ml)	1569.48	4174.9	2.66	2.17–3.26
AUC_0-∞_ (h·ng/ml)	1616.35	4207.38	2.60	2.14–3.16
C_max_ (ng/ml)	237.98	542.35	2.28	1.91–2.72

C_max_, peak plasma concentration; AUC_0-t_, area under the plasma concentration-time curve from time zero to time t; AUC_0-∞_, area under the plasma concentration-time curve from time zero to infinity.

**TABLE 5:** Pharmacokinetic (PK) properties of jaktinib, ZG0244 and ZG0245 after dose of 200 mg jaktinib in the fasted and fed state.

PK parameter	Dosage	
	200 mg fasted (n = 12)	200 mg fed (n = 12)
Jaktinib		
T_max_, median (min–max), h	2.00 (0.50–5.00)	4.00 (3.00–5.00)
C_max_, geometric mean (CV%), ng/ml	237.98 (69.0)	542.35 (32.1)
AUC_0-t_, geometric (CV%), h·ng/ml	1569.476 (79.5)	4174.895 (53.5)
AUC_0-∞_, geometric (CV%), h·ng/ml	1616.349 (78.6)	4207.378 (53.7)
T_1/2_, geometric (CV%), h	6.695 (66.6)	4.567 (30.4)
V_z_/F, geometric (CV%), L	1195.144 (123.0)	313.193 (27.7)
CL/F, geometric (CV%), L/h	123.736 (78.6)	47.536 (53.7)
ZG0244		
T_max_, median (min–max), h	3.00 (1.50–4.00)	5.00 (4.00–8.00)
C_max_, geometric mean (CV%), ng/ml	191.98 (42.2)	299.22 (24.7)
AUC_0-t_, geometric (CV%), h·ng/ml	1621.780 (34.2)	3206.911 (21.7)
AUC_0-∞_, geometric (CV%), h·ng/ml	1676.718 (34.9)	3242.764 (22.4)
T_1/2_, geometric (CV%), h	6.286 (50.8)	5.338 (36.5)
MR (AUC_0-∞_), geometric (CV%)	1.008 (50.1)	0.749 (42.7)
ZG0245		
T_max_, median (min–max), h	6.50 (3.00–14.00)	8.00 (4.00–14.00)
C_max_, geometric mean (CV%), ng/ml	28.37 (39.9)	55.12 (43.9)
AUC_0-t_, geometric (CV%), h·ng/ml	559.227 (55.9)	1158.099 (57.9)
AUC_0-∞_, geometric (CV%), h·ng/ml	685.143 (86.4)	1332.287 (69.5)
T_1/2_, geometric (CV%), h	13.986 (89.3)	12.918 (46.1)
MR (AUC_0-∞_), geometric (CV%)	0.477 (69.7)	0.348 (48.4)

T_max_, time to peak plasma concentration; C_max_, peak plasma concentration; AUC_0-t_, area under the plasma concentration-time curve from time zero to time t; AUC_0-∞_, area under the plasma concentration-time curve from time zero to infinity; T_1/2_, terminal elimination half-life; MR, metabolic rate; CV%, percentage coefficient of variation.

For metabolic transformation study, jaktinb and ZG0244 were detected in urine samples and in feces samples, jaktinib, ZG0244, ZG0245, ZG0243 and ZG0262 were detected. The 0–120 h accumulated excretion recovery ratio was 19.478% in urine and feces (Table 6).

**TABLE 6:** Accumulated excretion recovery ratio of jaktinib and metabolites in urine and feces.

PK parameter	Jaktinib (n = 6)	ZG0244 (n = 6)	ZG0245 (n = 6)	ZG0243 (n = 6)	ZG0262 (n = 6)	Total
Urine-Fe_0–120h_, geometric mean (%)	0.132	3.996	—	—	—	4.128
Feces-Fe_0–120h_, geometric mean (%)	12.681	0.395	1.000	1.072	0.202	15.350
Total (%)	12.813	4.391	1.000	1.072	0.202	19.478

Fe_0–120h_, accumulated excretion recovery ratio from time zero to 120 h.

### Safety and Tolerability

Overall, jaktinib was well tolerated in all the SAD and MAD cohorts except for 150 mg q12 h cohort. Treatment-related AEs (TRAEs) were reported in 18 (28.1%) and 37 (74.0%) volunteers in SAD and MAD cohorts. In the food effect cohort, 3 (25.0%) and 5 (41.7%)volunteers reported treatment-related AEs in the fasted and fed state. The most frequently reported treatment-related AEs were neutropenia, diarrhea, dizziness and headache. No deaths, SAEs and Grade ≥4 AEs were reported in all the cohorts.

In the SAD part, most reported TRAEs were Grade 1 except one Grade 3 Varicella, which was reported five days after administration and the subject recovered after the treatment of second-generation cephalosporin cefuroxime axetil. Headache (10.4%), positive urine erythrocyte (8.3%) were the most frequently reported AEs (≥5%) for volunteers who received jaktinib, and 0%, 6.3% for volunteers who received placebo. No dose-dependent TRAEs were observed (Table 7).

**TABLE 7:** Summary of treatment-related AEs (NCI CTCAE Grades) in single ascending dose cohorts.

Cohorts	25 mg (N = 6), n (%)		50 mg (N = 6), n (%)		100 mg (N = 6), n (%)		150 mg (N = 6), n (%)		200 mg (N = 6), n (%)		250 mg (N = 6), n (%)		300 mg (N = 6), n (%)		400 mg (N = 6), n (%)		Placebo (N = 16), n (%)	
Item	Grade 1–2	Grade 3	Grade 1–2	Grade 3	Grade 1–2	Grade 3	Grade 1–2	Grade 3	Grade 1–2	Grade 3	Grade 1–2	Grade 3	Grade 1–2	Grade 3	Grade 1–2	Grade 3	Grade 1–2	Grade 3
Headache	0	0	0	0	0	0	0	0	0	0	1 (16.7)	0	4 (66.7)	0	0	0	0	0
Urine erythrocyte positive	0	0	0	0	0	0	3 (50.0)	0	1 (16.7)	0	0	0	0	0	0	0	1 (6.3)	0
Urine leukocyte positive	0	0	0	0	0	0	2 (33.3)	0	0	0	0	0	0	0	0	0	1 (6.3)	0
Fever	0	0	0	0	0	0	0	0	0	0	1 (16.7)	0	1 (16.7)	0	0	0	0	0
Elevated serum creatinine	1 (16.7)	0	0	0	0	0	0	0	0	0	0	0	0	0	0	0	0	0
Hematuria	0	0	0	0	1 (16.7)	0	0	0	0	0	0	0	0	0	0	0	0	0
Lymphocytopenia	0	0	0	0	0	0	0	0	0	0	0	0	1 (16.7)	0	0	0	0	0
Neutrophilic granulocytosis	0	0	0	0	0	0	0	0	0	0	0	0	1 (16.7)	0	0	0	0	0
Fecal leukocytosis	0	0	0	0	0	0	1 (16.7)	0	0	0	0	0	0	0	0	0	0	0
Urinary sediment positive	0	0	0	0	0	0	1 (16.7)	0	0	0	0	0	0	0	0	0	1 (6.3)	0
Positive fecal occult blood	0	0	0	0	0	0	1 (16.7)	0	0	0	0	0	0	0	0	0	0	0
Dizziness	0	0	0	0	0	0	0	0	0	0	0	0	1 (16.7)	0	0	0	0	0
Upper abdominal pain	0	0	0	0	0	0	1 (16.7)	0	0	0	0	0	0	0	0	0	0	0
Mucous stool	0	0	0	0	0	0	1 (16.7)	0	0	0	0	0	0	0	0	0	0	0
Nausea	0	0	0	0	0	0	0	0	1 (16.7)	0	0	0	0	0	0	0	0	0
Anemia	0	0	0	0	0	0	0	0	0	0	0	0	0	0	1 (16.7)	0	0	0
Varicella	0	0	0	0	0	0	0	0	0	0	0	1 (16.7)	0	0	0	0	0	0
Hyperglycemia	0	0	0	0	0	0	1 (16.7)	0	0	0	0	0	0	0	0	0	0	0
Supraventricular arrhythmia	0	0	0	0	0	0	0	0	0	0	0	0	0	0	0	0	2 (12.5)	0

Note: No treatment-related Grade 4–5 AEs observed in all single ascending dose cohorts.

AE, adverse event; NCI CTCAE, National Cancer Institute Common Terminology Criteria for Adverse Events.

In the MAD part, most reported TRAEs were Grade 1 or 2. All the Grade 3 AEs were neutropenia with 1 (12.5%) in the 100 mg q12h cohort and 5 (62.5%) in the 150 mg q12h cohort. The volunteers suffered Grade 3 neutropenia stopped jaktinib administration immediately and recovered to normal without additional treatment. Neutropenia (27.5%), diarrhea (22.5%), dizziness (17.5%) and leukopenia (12.5%) were the most frequently reported TRAEs (≥10%) in jaktinib group showed a greater proportion than placebo group (10%, 0%, 20% and 0%, respectively). No obvious dose-dependent TRAEs were observed (Table 8).

**TABLE 8:** Summary of treatment-related AEs (NCI CTCAE Grades) in multiple ascending dose cohorts.

Cohorts	100 mg q24h (N = 8), n (%)		150 mg q24h (N = 8), n (%)		100 mg q12h (N = 8), n (%)		200 mg q24h (N = 8), n (%)		150 mg q12h (N = 8), n (%)		Placebo (N = 10), n (%)	
Item	Grade 1–2	Grade 3	Grade 1–2	Grade 3	Grade 1–2	Grade 3	Grade 1–2	Grade 3	Grade 1–2	Grade 3	Grade 1–2	Grade 3
Neutropenia	0	0	0	0	4 (50.0)	1 (12.5)	1 (12.5)	0	0	5 (62.5)	1 (10.0)	0
Diarrhea	0	0	5 (62.5)	0	3 (37.5)	0	1 (12.5)	0	0	0	0	0
Dizziness	1 (12.5)	0	0	0	6 (75.5)	0	0	0	0	0	2 (20.0)	0
Leukopenia	0	0	0	0	1 (12.5)	0	1 (12.5)	0	3 (37.5)	0	0	0
Elevated alanine aminotransferase	1 (12.5)	0	0	0	1 (12.5)	0	1 (12.5)	0	0	0	0	0
Urine erythrocyte positive	2 (25.0)	0	1 (12.5)	0	0	0	0	0	0	0	0	0
Upper abdominal pain	0	0	0	0	1 (12.5)	0	2 (25.0)	0	0	0	1 (10.0)	0
Fatigue	3 (37.5)	0	0	0	0	0	0	0	0	0	0	0
Elevated aspartate aminotransferase	1 (12.5)	0	0	0	1 (12.5)	0	0	0	0	0	0	0
Hematuresis	0	0	1 (12.5)	0	0	0	0	0	1 (12.5)	0	0	0
Supraventricular arrhythmia	0	0	1 (12.5)	0	1 (12.5)	0	0	0	0	0	0	0
Urinary tract infection	1 (12.5)	0	1 (12.5)	0	0	0	0	0	0	0	2 (20.0)	0
Elevated amylase	0	0	0	0	0	0	0	0	1 (12.5)	0	0	0
Elevated serum creatinine	0	0	0	0	0	0	1 (12.5)	0	0	0	0	0
Elevated serum urea	0	0	0	0	0	0	1 (12.5)	0	0	0	0	0
Neutrophilic granulocytosis	1 (12.5)	0	0	0	0	0	0	0	0	0	0	0
Decreased plasma protein	0	0	0	0	1 (12.5)	0	0	0	0	0	0	0
Leukocytosis	1 (12.5)	0	0	0	0	0	0	0	0	0	0	0
Urine leukocyte positive	1 (12.5)	0	0	0	0	0	0	0	0	0	0	0
Increased bile acid	0	0	0	0	0	0	1 (12.5)	0	0	0	0	0
Abdominal discomfort	0	0	0	0	0	0	0	0	1 (12.5)	0	0	0
Constipation	0	0	0	0	0	0	0	0	1 (12.5)	0	0	0
Emesis	0	0	0	0	0	0	0	0	1 (12.5)	0	0	0
Nodal arrhythmia	0	0	0	0	1 (12.5)	0	0	0	0	0	0	0
Sinus bradycardia	1 (12.5)	0	0	0	0	0	0	0	0	0	0	0
Anemia	1 (12.5)	0	0	0	0	0	0	0	0	0	2 (20.0)	0
Headache	0	0	0	0	0	0	1 (12.5)	0	0	0	1 (10.0)	0
Stomatitis pain	0	0	0	0	1 (12.5)	0	0	0	0	0	0	0
Urticaria	0	0	0	0	0	0	0	0	1 (12.5)	0	0	0
Arthralgia	0	0	0	0	0	0	0	0	0	0	1 (10.0)	0

Note: No treatment-related Grade 4–5 AEs observed in all multiple ascending dose cohorts.

AE, adverse event; NCI CTCAE, National Cancer Institute Common Terminology Criteria for Adverse Events.

In the food effect part, all TRAEs were mild to moderate in severity. A higher incidence of TRAEs were observed in the fed state but the difference was not statistically significant (Table 9).

**TABLE 9:** Summary of treatment-related AEs (NCI CTCAE Grades) in food effect cohort.

State	200 mg fasted (N = 12), n (%)		200 mg fed (N = 12), n (%)	
Item	Grade 1–2	Grade 3	Grade 1–2	Grade 3
Diarrhea	1 (8.3)	0	1 (8.3)	0
Headache	0	0	2 (16.7)	0
Anemia	1 (8.3)	0	1 (8.3)	0
Upper abdominal pain	1 (8.3)	0	0	0
Dizziness	0	0	1 (8.3)	0
Elevated alanine aminotransferase	1 (8.3)	0	0	0
Urine leukocyte positive	0	0	1 (8.3)	0
Urinary tract infection	1 (8.3)	0	0	0

Note: No treatment-related Grade 4–5 AEs observed in food effect cohorts.

AE, adverse event; NCI CTCAE, National Cancer Institute Common Terminology Criteria for Adverse Events.

## Discussion

This phase 1 study evaluated the safety, tolerability, PK characteristics of SAD, MAD and food effect of jaktinib, in healthy Chinese volunteers. The baseline demographic characteristics were similar across all the cohorts.

After single administration, jaktinib was rapidly absorbed in plasma with high inter-individual variations in PK parameters. ZG0244 was the most abundant metabolite, followed by ZG0245, with the MR (AUC_0-∞_) 0.789–1.325 and 0.279–0.584 respectively after SAD of 25–400 mg jaktinib. ZG0244 appeared in plasma almost simultaneously with jaktinib and revealed a similar PK characteristics. For ZG0245, it appeared slowly in plasma and the T_max_ was about several hours longer than jaktinib and ZG0244. There was a trend of dose-proportional increase in exposure to jaktinib with no obviously differences observed for t_1/2_.

In MAD cohorts, visual inspection of trough concentration data, steady-state plasma concentration were reached at approximately 4 days for jaktinib. The T_max_ were comparable between the first and the last dose. No obvious accumulation were observed for jaktinib and ZG0244, while moderate accumulation appeared in ZG0245. A longer t_1/2_ with the accumulation gave ZG0245 a much higher initial concentration at pre-dose of day 10 in 100 mg q12h than 200 mg q24h. As a result, the day 10 0–12 h exposure of ZG0245 was higher in 100 mg q12h than 200 mg q24 h. Compared the day 10 0–12 h ZG0245 exposure between 100 mg q12h and 150 mg q12h, the AUC_0–12h_ was a little lower in 150 mg q12h (725.156 h·ng/ml) than in 100 mg q12h (807.632 h·ng/ml), this may attribute to a higher variable coefficient (CV) of ZG0245, 118.7% in 100 mg q12h and 103.5% in 150 mg q12h, respectively. High-fat meals increased the exposure of jaktinib and metabolites and delayed the time to peak concentration. Administration without food is recommended for the future studies based on the following considerations: firstly, the concentrations in plasma after administrations with high-fat food were very high and additional study for food impact with low-fat meal need to be conducted; And secondly, for agents of long-term use, it is difficult to administrate after high-fat meal, because some MF patients can't tolerated the high-fat meals, especially for patients of q12h administration. Considering these differences in diet and disease state of MF patients, administrations without food can achieve a relatively consistent exposure to obtain favorable effect. Feces was the main way of excretion and the unmetabolized jaktinib was the main detected substance in the excrements, which showed potential of further test in patients with renal insufficiency.

In the SAD part, most AEs were mild in severity. One volunteer at dose 250 mg experienced Grade 3 varicella, which was considered related to the pharmacological mechanism of JAK inhibitor. Virus infection (herpes zoster) was also observed in the RESPONSE trial of ruxolitinib with the incidence rates 4.7% and 0% in ruxolitinib and control groups, respectively (Kiladjian et al., 2020). Headache was the most common AEs in jaktinib SAD part, while no headache occurred in the single dose 400 mg cohort and only one in MAD cohorts. In general, jaktinib were well tolerated with no dose-dependent AEs observed in SAD cohorts.

In the MAD part, neutropenia, diarrhea and dizziness were the most common AEs. These AEs have been also observed in ruxolitinib and momelotinib clinical trials (Verstovsek et al., 2012; Pardanani et al., 2018; Kiladjian et al., 2020). The incidence of neutropenia was higher in 100 mg q12h cohort (four at Grade 1–2 and one at Grade 3) than in 200 mg q24h cohort (one at Grade 1), speculating that q24h dosing schedule may be safer than q12h. Meanwhile, the higher severity of neutropenia in 150 mg q12h cohort (five at Grade 3) than 100 mg q12h cohort suggested it might be occurred in a dose-dependent manner. Other AEs in MAD part were mild and moderate in severity. In the food effect part, no Grade ≥3 AEs were reported and the incidence of AEs in the fed state were a little higher than that in the fasted state, which may due to higher PK exposure in fed state.

In conclusion, the current study showed that single oral administration of jaktinib 25–400 mg and multiple doses up to 200 mg q24h were well tolerated with no DLTs observed. The accumulated safety and PK data in healthy subjects support further evaluation in patient clinical trials for the treatment of MF.

## Data Availability Statement

The original contributions presented in the study are included in the article/Supplementary Material, further inquiries can be directed to the corresponding author.

## Ethics Statement

The studies involving human participants were reviewed and approved by the Ethics Committee of the First Hospital of Jilin University, China. The patients/participants provided their written informed consent to participate in this study.

## Author Contributions

JL and YD wrote the article. YD, JL and BL designed the research. JL, BL, HY, XZ and HW performed the clinical trial. JL, XZ and YD analyzed the data.

## Funding

The work was funded by Suzhou Zelgen Biopharmaceuticals Co., Ltd. The work was also financially supported by the National Major Scientific and Technological Special Project for “Significant New Drug Development” during the Thirteenth Five-Year Plan Period of China (Project Nos. 2017ZX09304004 and 2017ZX09101001-002-004), the National Natural Science Foundation of China (Project No. 81602897).

## Conflict of Interest

BL and HY are employees of Suzhou Zelgen Biopharmaceuticals Co., Ltd. The remaining authors declare that the research was conducted in the absence of any commercial or financial relationships that could be construed as a potential conflict of interest.
