# Supplementary material for: A Phase I, Randomized, Double-Blind, Placebo-Controlled, Single Ascending Dose, Multiple Ascending Dose and Food Effect Study to Evaluate the Tolerance, Pharmacokinetics of Jaktinib, a New Selective Janus Kinase Inhibitor in Healthy Chinese Volunteers
Source: Front Pharmacol. 2020 Dec 14;11:604314. doi: 10.3389/fphar.2020.604314 (PMC7849180; doi:10.3389/fphar.2020.604314)
Supplement: Supplementary file 1 [file datasheet1.pdf]

CFDA Clinical Study Approval Number: 2016L10574, 2016L10575

Clinical Study Protocol

A Randomized, Double-blind, Escalating Single- and Multiple-dose Study on Tolerability, Pharmacokinetics, and Food Effect of Jaktinib Hydrochloride Tablet or Placebo in Healthy Volunteers

Protocol No.: ZGJAK001

Version No.: 1.0

Version Date: May 02, 2017

Clinical Trial Institution: Phase I Clinical Trial Laboratory of the First Hospital of Jilin University

Clinical Trial Sponsor: Suzhou Zelgen Biopharmaceuticals Co., Ltd.

Sample Bioanalysis Unit: Suzhou Haike Pharmaceutical Solutions Co., Ltd.

The information contained in this document is proprietary and confidential. The document must not be copied, circulated, or distributed by any other ways without the written consent of Suzhou Zelgen Biopharmaceuticals Co., Ltd.

A Randomized, Double-blind, Escalating Single- and Multiple-dose Study on Tolerability, Pharmacokinetics, and Food Effect of Jaktinib Hydrochloride Tablet or Placebo in Healthy Volunteers

Protocol/Amendment Number	Version Number	Version Date	Revision
ZGJAK001	1.0	May 02, 2017	Null

Signature Page

I have read this clinical trial protocol (protocol number: ZGJAK001, version 1.0, version date: May 02, 2017), and agree to perform my duties in accordance with Chinese law, Declaration of Helsinki, CFDA GCP, as well as the study protocol. This study will only be implemented after receiving the approval from the ethics committee.

During the study, I will strictly follow the protocol requirements. If it is necessary to modify the protocol, such modification request should be approved by the sponsor, and approved by the ethics committee again before implementation, unless measures must be immediately taken to protect the safety, rights and interests of the subjects.

I will keep this protocol and relevant information confidential.

Sponsor: Suzhou Zelgen Biopharmaceuticals Co., Ltd.

Project Leader (printed): Wu Liqing

(Signature):______________________Date:________ MM DD YYYY

Clinical Study Institution: Phase I Clinical Trial Laboratory of the First Hospital of Jilin University

Principal Investigator (printed): Ding Yanhua

(Signature):______________________Date:________ MM DD YYYY

Table of Contents

Table of Contents 2
Protocol Abstract........ 4
1 Background........ 18
2 Justification of Trial Design........ 46
Overall Design 46
Overview of Preclinical Study 46
Preclinical Pharmacodynamic Study........ 48
Preclinical Toxicology Test Results 52
Preclinical Pharmacokinetic Properties........ 61
Detailed Trial Design 68
Considerations for Basic Design........ 68
Study Procedures 69
Screening 69
Single Ascending Dose (SAD)........ 70
Multiple Ascending Dose Study (MAD)........ 72
Food Effects and Metabolism Studies........ 75
PK Blood Sample Collection 78
Nomenclature of Biological Samples........ 78
Biological Sample Collection and Processing 79
3 Biological Sample Transport and Cold Chain Logistics 80
4 Subject Selection........ 80
Inclusion Criteria (Must Meet All Criteria to be Eligible) 80
Exclusion Criteria (Excluded if any criteria is met)........ 80
Rejection Criteria 82
Managing Subject Dropouts........ 82
Criteria for Study Termination........ 84
5 Trial Procedures 84
Administration 84
Standard Meal (High-Fat, 800-1000 kcal) 85
Investigational Drug Information........ 85
Acceptance, Dispensing and Storage of Investigational Drugs........ 86
Administration Method........ 87
Dosage Determination 87
Administration Time 88
Blinding 88
Other Drugs Used Before and During the Trial 88
Drugs Not Permitted During the Trial........ 88
Appropriate Use of Drugs during the Trial 89
Contraindications and Restrictions 89
Medication Compliance 90
6 PK Parameters and Safety Assessment Indexes 90
PK Parameters for Evaluation and Safety Assessment Indexes 90
PK Parameters for Evaluation........ 90
Safety Assessment Indexes 90
Assessment of Adverse Events and Serious Adverse Event........ 90
Trial Procedure Form........ 96
Sample Collection and Storage........ 100
Primary Pharmacokinetic Parameters 100
7 Quality Assurance 101
Quality Assurance on the Clinical Trial 101
Quality Assurance on Sample Testing........ 101

Quality Assurance on Data Transfer, Calculation and Report 102
8 Statistical Method and Sample Size Determination 103
Statistical Analysis 103
Study Population 103
Statistical Analysis of Pharmacokinetic Parameters 103
Analysis of Safety Variables 105
Sample Size Determination 105
9 Management of Abnormalities during the Trial 105
Criteria for Study Termination 105
Risk Assessment and Risk Management 106
Protocol Deviation or Violation 106
10 Protocol Revision History 106
11 Data Processing and Record Retention 106
Requirements of Filling Data 106
Database Establishment and Data Entry 107
12 Ethical Standards and Informed Consent 107
Ethical Standards 107
Informed Consent 108
13 Study Report 108
14 Data Retention 109
15 Study Site 109
References 110
Appendix 1: Deviation of Blood Collection Time Points 111
Appendix 2: Clinical Laboratory and Imaging Tests 112
Appendix 3: Creatinine Clearance Formula 114
Appendix 4: Anaphylactic Shock Treatment Plan 115
Appendix 5: Risk Management Plan 117
Appendix 6: Contraceptive Measures, Definition of Women of Childbearing Age, and Contraception Requirements 121
Appendix 7:Standard Western Meal(800-1000 kcal)Ingredients, Preparation Methodsand Calories 123
Appendix 8: Known CYP Liver Enzyme Activity Inhibitorsand Inducers 125

Protocol Abstract

**Study Title**	**A randomized, double-blind, escalating single- and multiple-dose study on tolerability, pharmacokinetics, and food effect of Jaktinib hydrochloride tablet or placebo in healthy volunteers**
**Name of Investigational Drug**	**Jaktinib Hydrochloride Tablet**
**Study Objectives**	**Primary Objective**<br>✧ To evaluate the safety and tolerability after single and multiple oral administration of Jaktinib hydrochloride tablet at different doses in healthy subjects, and to find any potential dose-limiting toxicity (DLT) and the maximum tolerated dose (MTD).<br>**Secondary Objectives**<br>✧ To evaluate the PK parameters in humans after single and multiple oral administration of Jaktinib hydrochloride tablets;<br>✧ To observe the effects of high-fat diet on the metabolism of Jaktinib hydrochloride tablets;<br>✧ Analysis of drug metabolites and metabolism study.
**Clinical Study Protocol No.**	ZGJAK001
**Sponsor**	Suzhou Zelgen Biopharmaceuticals Co., Ltd.
**Clinical Study Approval No.**	2016L10574, 2016L10575
**Registration Category**	Chemical Drug Class 1 New Drug
**Trial Site**	Phase I Clinical Trial Laboratory of the First Hospital of Jilin University
**Trial Phase**	Phase Ia
**Overall Design**	A single-center, randomized, double-blind, placebo-controlled, escalating single- and multiple-dose study evaluating the effects of food on the PK and metabolism of Jaktinib Hydrochloride

**Dosage Regimen**	(1) Fasting condition - based on the randomization table, subjects will take either the investigational drug or placebo with 240 ml of warm water after overnight fast of at least 10 hours.<br>(2) After the standard meal (high-fat, 800-1000 kcal) - after overnight fast of at least 10 hours, subjects should be provided with standard meal 30 minutes prior to the drug administration. The eating speed should be controlled and the meal must be finished before dosing (based on the randomization list) with 240 ml of warm water.<br>(3) Multiple-Dose - Administered with 240 ml of warm water under the fasting condition on the morning of D1 and D10. On D2-D9, take breakfast 0.5 h after administration, take lunch 4 hours after administration, and take dinner 10 hours after administration. q12h dosing means drug is given 2 hours after supper, at 12 hours intervals from the last dose.<br>**Investigational Drug**<br>Jaktinib hydrochloride tablets, manufactured by WuXi AppTec Co., Ltd., and provided by Suzhou Zelgen Biopharmaceuticals Co., Ltd. Lot number: 1703FP2157-01, specification: 50 mg/tablet (25 mg/tablet is 50 mg/tablet divided into halves, lot number: 1703ZG2157-01);<br>Manufacture Date: see product packaging;<br>Expiration Date: see product packaging.<br>**Control Drug**<br>Jaktinib hydrochloride placebo tablet, manufactured by WuXi AppTec Co., Ltd., and provided by Suzhou Zelgen Biopharmaceuticals Co., Ltd. Lot number: 1703FP2158-01, specification: 50 mg/tablet (25 mg/tablet is 50 mg/tablet divided into halves, lot number: 1703ZG2158-01);<br>Manufacture Date: see product packaging;<br>Expiration Date: see product packaging.
**Number of Subjects**	Single Ascending Dose (SAD): 64 subjects;<br>Multiple Ascending Dose (MAD): 50 subjects;<br>Effects of food on PK and drug metabolism: 12 subjects;<br>Male to female ratio close to 1:1;
**Study Duration**	April 2017 - June 2018
**Dose**	25 mg, 50 mg, 100 mg, 150 mg, 200 mg, 250 mg, 300 mg, and 400 mg
**Trial Design**	This is a single-center, randomized, double-blind, placebo-controlled, escalating single- and multiple-dose study evaluating the effects of food on

	PK and drug metabolism. The aim is to evaluate the effects of tolerability, PK characteristics, drug metabolism, and food of single- and multiple-dose Jaktinib hydrochloride tablets on PK in healthy subjects.<br>**Single Ascending Dose (SAD)**<br>The subjects are divided into **8** dose groups: 25 mg (group 1), 50 mg (group 2), 100 mg (group 3), 150 mg (group 4), 200 mg (group 5), 250 mg (group 6), 300 mg (group 7), and 400 mg (group 8).<br>Group 1 to 8 will enroll 8 subjects each, **6** receiving the investigational drug, and **2** receiving placebo. The male to female ratio should be close to 1:1. Subjects in each group receive a single dose under the fasting condition, and undergo tolerability assessment on **D3**. Subjects of different dose groups are enrolled sequentially. The next dose group is tested only if the previous group is well tolerated.<br>**Multiple Ascending Dose (MAD)**<br>A total of 5 dosage groups: 100 mg q24h (group 9), 150 mg q24h (group 10), 100 mg q12h (group 11), 200 mg q24h (group 12), 150 mg q12h (group 13). The dose selection of each group may be adjusted based on the results of SAD study.<br>Each group will enroll 10 subjects, 8 receiving the investigational drug, and 2 receiving placebo. The male to female ratio should be close to 1:1. Each group will receive 10 or 19 doses under the fasting condition, and undergo tolerability assessment on D4, D7, and D12. Subjects of different dose groups are enrolled sequentially. The next dose group is tested only if the previous group is well tolerated.<br>**Effects of food on PK and Drug Metabolism**<br>This is a randomized, two-period, crossover study evaluating the effects of food on PK and drug metabolism. Totally 12 healthy subjects are randomized into group A and group B, with 6 subjects in each group. The male to female ratio should be close to 1:1. Subjects will receive 200 mg Jaktinib hydrochloride tablets or the MTD of Jaktinib hydrochloride tablets observed during the MAD study. The specific dose is selected together by the investigator and the sponsor. Group A will receive Jaktinib hydrochloride tablets under the fasting condition (Period 1), and after washout, the subject will receive Jaktinib hydrochloride tablets after the meal (Period 2). Group B will receive Jaktinib hydrochloride tablets after the meal (Period 1), and after washout, the subject will receive Jaktinib hydrochloride tablets under the fasting condition (Period 2). The washout period is 5 days between the two periods. PK urine and feces will be collected for Group A in Period 1 under

	the fasting conditions. Subjects would withdraw from the trial after the tolerability assessment on D8.<br>Subjects may withdraw from the trial if there are no clinically significant abnormalities in laboratory tests and clinical observation. If the abnormality has clinical significance, the investigator may determine to continue follow-up until the subject recovers to normal or stable, or is lost to follow-up.<br>During the trial, blood samples for PK analysis are collected at scheduled time points according to the protocol. Since Jaktinib has never been used in humans, no human PK parameters can be used as reference. Therefore, sampling time points may be finely adjusted based on the PK results of first set.
**Inclusion Criteria**	(1) Sign the informed consent form, and fully understand the content, procedure and possible adverse events before the trial starts;<br>(2) Capable of completing the trial according to trial protocol;<br>(3) Subjects (including male subjects) do not have birth plans in the next 6 months, and agree to adopt effective contraceptive measures. Refer to Appendix 6 for specific contraceptive measures;<br>(4) Male and female subjects of 18-45 years of age (inclusive);<br>(5) Male subjects must not weigh less than 50 kg, and female subjects must not weight less than 45 kg. Body Mass Index (BMI) = weight (kg)/ height^2 (m^2), BMI between 18-28 (inclusive);<br>(6) Health status: subjects do not have a clinically significant medical history related to the heart, liver, kidneys, digestive tract, nervous system, respiratory system, mental disorders or metabolic disorders;<br>(7) Normal physical examination and vital signs, or abnormal physical examination and vital signs with no clinical significance.
**Exclusion Criteria**	1) Smoke more than 5 cigarettes per day within 3 months before the trial starts;<br>2) Allergic to the investigational drug or its excipients, or allergies to various drugs and food;<br>3) Having a history of drug abuse and/or alcohol abuse (14 units of alcohol consumed per week: 1 unit = 285 mL of beer, 25 mL of liquor, or 100 mL of wine);<br>4) Blood donation or massive blood loss (> 450 mL) within 3 months prior to the first dose of investigational drug;<br>5) Having swallowing difficulty or a history of gastrointestinal disorders that may affect drug absorption;

	6) Suffering from any disease that increases the risk of bleeding, such as hemorrhoids, acute gastritis, or gastric and duodenal ulcers;<br>7) Have taken any drugs that may affect liver enzyme activities 28 days prior to the first dose of investigational drug;<br>8) Have taken any prescription drugs, over-the-counter drugs, any vitamin products or herbal medicines within 14 days prior to the first dose of investigational drug;<br>9) Having a special diet (including dragon fruit, mango, grapefruit, and/or diet rich in xanthine) within 2 weeks prior to the trial, or intense physical activity, or other factors that may affect drug absorption, distribution, metabolism, and excretion;<br>10) Having concomitantly used the following inhibitors or inducers of CYP3A4, P-gp or Bcrp, such as itraconazole, ketoconazole, or dronedarone;<br>11) Have undergone major changes in diet or physical activity habits recently;<br>12) Have taken the investigational drug or participated in a clinical trial within 3 months prior to the first dose of investigational drug;<br>13) Subjects who are unable to tolerate the high-fat meal (2 hard-boiled eggs of 100 g, 20 g of bacon, a pieces buttered toast of 50 g, 115 g of fries, 240 ml of whole milk) - only applicable to subjects participating in the postprandial study;<br>14) Abnormal ECG result with clinical significance;<br>15) Female subjects who are lactating, or who test positive for pregnancy during the screening or during the trial;<br>16) Clinical laboratory abnormalities with clinical significance, or other clinical findings indicating the following diseases with clinically significance (including but not limited to gastrointestinal, renal, hepatic, neural, hematological, endocrine, tumors, pulmonary, immune, psychiatric, or cerebrovascular diseases);<br>17) Positive hepatitis (including Hep B and Hep C), AIDS, and syphilis during screening;<br>18) Acute disease during the screening or before the administration of investigational drug;<br>19) Have taken chocolate, any food or drink containing caffeine or xanthine 48 hours prior to the first dose of investigational drug;<br>20) Have taken any alcoholic products within 24 hours before dosing;<br>21) Subjects with positive alcohol and drug screening results, or subjects

	with a history of drug abuse in the past 5 years, or a history of narcotics within 3 months prior to the trial.
**Withdrawal Criteria**	Determined by the investigator<br>If the enrolled subjects is unsuitable to continue the trial, the investigator will determine to ask the subject to withdraw from the study.<br>• The investigator believes there is a need to discontinue the study from an ethical standpoint.<br>• A serious adverse event (SAE) occurs, making the subject unsuitable to continue the trial.<br>• The investigator determines that withdrawal from the study is in the best interest of subject.<br>• Subject has poor compliance, including the following:<br>a. Subject does not take the medication or receive examinations as required;<br>b. Subject takes medications or food that will affect the safety assessment and the PK analysis results;<br>c. Subject is smoking or drinking (the investigator will determine whether subject needs to withdraw from the study);<br>d. Other behaviors of the subjects that may affect the results of the study.<br>Subject voluntarily withdraws from the study<br>According to the informed consent form, subject reserves the right to withdraw from the study; or the subject does not withdraw the informed consent, but no longer accepts medication and examinations, and is lost to follow-up (also considered as withdrawal, or dropout). The reason for the withdrawal should be recorded, and a safety assessment should be conducted. For subjects participating in the MAD study, PK blood samples should be collected at the scheduled time point for the last dose.
**Rejection Criteria**	The principal investigator, the sponsor, and the statistician should decide whether individual cases should be rejected from the statistical analysis of trial data. When one of the following situations occurs, the principal investigator should decide whether the subject should be rejected based on the factors such as the degree of completion of the trial and the reason for withdrawal, and provide a relevant explanation.<br>1） Subjects, who violate the inclusion/exclusion criteria, should not be included into the trial.<br>2） An adverse event occurs during the trial that results in the subject being unable to continue or complete the trial as scheduled.

	3) Subject has poor compliance during the trial, such as subject does not receive the study drug, or samples cannot be collected according to the trial protocol for PK and safety evaluations.<br>4) During the trial, the investigator determines that the subject cannot continue to participate in the trial and actually terminates subject's participation in the trial.
**Criteria for Study Termination**	1) According to NCI CTCAE version 4.03, if more than 1/3 of the subjects experience drug-related grade 3 non-hematologic adverse events or grade 4 hematologic adverse events, or 1 case experiences drug-related serious adverse event (SAE), then that suggests subjects cannot tolerate the drug.<br>2) During the trial, a major error is found in the clinical trial protocol that makes it difficult to evaluate the study drug.<br>3) The sponsor will request the premature termination of the study to fully ensure the rights and safety of the subjects.<br>4) The CFDA or ethics committee orders the premature termination of the study due to some reasons.
**Tolerability Criteria and Dose Ascending Method:**	**Maximum Tolerated Dose (MTD)**<br>According to NCI-CTCAE v4.03 evaluation criteria, dose-limiting toxicity (DLT) is defined as drug-related grade 3 non-hematologic or grade 4 hematologic adverse events experienced by more than 1/3 of subjects in a dose group or experience drug-related serious adverse events occurring in 1 subject of a dose group, that suggests an evident intolerance. The dose of the previous group is defined as the maximum tolerated dose (MTD).<br>The tolerability assessment is done 48 hours (D3) after subjects receive the medication at 25 mg (50 mg/tablet, 1/2 tablet/day, qd, group 1), 50 mg (50 mg/tablet, 1 tablet/day, qd, group 2), 100 mg (50 mg/tablet, 2 tablets/day, qd, group 3), 150 mg (50 mg/tablet, 3 tablets/day, qd, group 4), 200 mg (50 mg/tablet, 4 tablets/day, qd, group 5), 250 mg (50 mg/tablet, 5 tablets/day, qd, group 6), 300 mg (50 mg/tablet, 6 tablets/day, qd, group 7), and 400 mg (50 mg/tablet, 8 tablets/day, qd, group 8). If tolerated, the study moves on to the next dose group.

	After completing the tolerability assessment for the single-dose 100 mg group, if tolerated, the tolerability assessment for the multiple-dose 100 mg q24h group can be started.<br>100 mg q24h (50 mg/tablet, 2 tablets/day, q24h, group 9), 150 mg q24h (50 mg/tablet, 3 tablets/day, q24h, group 10), 100 mg q12h (50 mg/tablet, 4 tablets/day, q12h, group 11), 200 mg q24h (50 mg/tablet, 4 tablets/day, q24h, group 12), 150 mg q12h (50 mg/tablet, 6 tablets/day, q12h, group 13) are administered for 10 consecutive days, a total of 10 times for the q24h groups, and a total of 19 times for the q12h groups (the dose is only given on the morning of D10). The tolerability assessment is done 264 h (D12) after hospitalization and observation, and if tolerated, the study moves on to the next dose group.<br>The study on the effects of food on PK and drug metabolism is carried out after the single-dose group of the similar dosage demonstrates tolerability.<br>If the highest dose group still shows tolerability, but have reached the maximum dose designed for the trial, then the trial can be stopped.
**Tolerability Assessment Indexes**	Observe any adverse events that occur during the trial, record the clinical manifestation, severity, start date, stop date, duration, measures taken, and outcome, and determine the relevance to the investigational drug.<br>The tolerability assessment include adverse events (AE), clinical laboratory tests (routine blood test, blood biochemistry, coagulation tests, routine urinalysis, routine stool test (include fecal occult blood)), vital signs (blood pressure, pulse, body temperature, respiratory rate), 12-lead ECG and physical examinations. Creatinine clearance is calculated using CKD-EPI equation.<br>**The date of examinations:**<br>**Single Ascending Dose (SAD)**<br>Laboratory tests (routine blood, blood biochemistry, coagulation tests, routine urinalysis, and stool analysis (include fecal occult blood)): screening period, D3;<br>Symptoms and physical examinations: screening period, D3;<br>12-Lead ECG: screening period, D3;<br>Vital signs: screening period, hospital admission, within 1 hour before first dose, 2 h, 6 h, 24 h, and 48 h after dosing.<br>**Multiple Ascending Dose (MAD)**<br>Laboratory tests: routine blood, blood biochemistry, coagulation, routine

	urinalysis, stool routine (include fecal occult blood) are tested during screening period, D4, D7, and D12; routine blood is tested on D10;<br>Symptoms and physical examinations: screening period, D4, D7, and D12;<br>12-Lead ECG: screening period, D4, D7, and D12;<br>Vital signs: screening period, hospital admission, within 1 hour before the first dose, 2 h, and 6 h after dosing; within 1 hour before dose administration on D2, D3, D4, and D7; within 1 hour before the last dose and 2 h, 6 h, 24 h, and 48 h after starting the last dose.<br>**Effects of food on PK and drug metabolism**<br>Laboratory tests (routine blood, blood biochemistry, coagulation tests, routine urinalysis, and stool routine (include fecal occult blood)): screening period, D8;<br>Symptoms and physical examinations: screening period, D8;<br>12-Lead ECG: screening period, D8;<br>Vital signs: screening period, hospital admission; in Period 1 - within 1 hour before dose administration and 2 h, 6 h, 24 h, 48 h, 72 h, 120 h after starting the dose; in Period 2 - 2 h, 6 h, 24 h, and 48 h after starting the dose.
**Pharmacokinetic Assessment Indicators**	**Plasma Samples:**<br>**Single Ascending Dose (SAD):**<br>Samples are collected within 30 minutes before dosing, 0.25 h, 0.5 h, 1 h, 1.5 h, 2 h, 3 h, 4 h, 5 h, 6 h, 8 h, 12 h, 14 h, 24 h, 36 h, and 48 h after dosing. 4 mL of samples are collected at each sampling time point.<br>**Multiple Ascending Dose (MAD):**<br>Samples are collected as following: within 30 minutes before dosing, and 0.25 h, 0.5 h, 1 h, 1.5 h, 2 h, 3 h, 4 h, 5 h, 6 h, 8 h, 12 h after dosing on D1; 30 minutes before dosing on the morning of D2, D3, D4, D6 and D7; 30 minutes before dosing, 0.25 h, 0.5 h, 1 h, 1.5 h, 2 h, 3 h, 4 h, 5 h, 6 h, 8 h, 12 h, 24 h, 36 h, and 48 h after dosing on D10. 4 mL of samples are collected at each sampling time point.<br>**Effects of food on PK and drug metabolism:**<br>**Group A in Period 1 under the fasting conditions (D1):**<br>PK blood samples: collected within 30 minutes before dosing, 0.25 h, 0.5 h, 1 h, 1.5 h, 2 h, 3 h, 4 h, 5 h, 6 h, 8 h, 12 h, 14 h, 24 h, 36 h, 48 h, 72 h, 96 h, and 120 h after dosing;<br>**Group A in Period 2 after standard meal (D6) and Group B in two Periods (D1, D6)**:

	PK blood samples: collected within 30 minutes before dosing, 0.25 h, 0.5 h, 1 h, 1.5 h, 2 h, 3 h, 4 h, 5 h, 6 h, 8 h, 12 h, 14 h, 24 h, 36 h, and 48 h after dosing<br>**Urine**:<br>The study on the effects of food is carried out at the dose of 200 mg or MTD observed during the multiple-dose study. Urine samples of Group A are collected under the fasting conditions before the first dose, 0-6 h, 6-12 h, 12-24 h, 24-48 h 48-72 h 72-96 h, 96-120 h after dosing.<br>**Feces**:<br>The study on the effects of food is carried out at the dose of 200 mg or MTD observed during the multiple-dose study. Feces of Group A are collected under the fasting conditions during 0-120 h after the first dose if subject has bowel movement.<br>**Single-dose PK parameters include:**<br>• Time to peak (Tmax)<br>• Peak concentration (C_{max})<br>• Elimination half-life ($t_{1/2}$)<br>• Area under the plasma concentration-time curve at an interval between time 0 (first dose) and infinitely great ($AUC_{0-\infty}$)<br>• Area under the plasma concentration-time curve at an interval between time 0 (first dose) and 12 h (AUC_{0-12h})<br>• Area under the plasma concentration-time curve at an interval between time 0 (first dose) and t (AUC_{0-t})<br>• Amount of drug excreted in urine from time 0 to 120 h after administration (Ae_{0-120h})<br>• Amount of drug excreted in feces from time 0 to 120 h after administration (Ae_{0-120h})<br>• Cumulative excretion rate of the drug in feces and urine<br>• Oral clearance (CL/f)<br>• Renal clearance (CLr/f)<br>**Steady-state pharmacokinetic parameters include:**<br>• Time to peak ($T_{max,ss}$)<br>• Peak concentration ($C_{max,ss}$)<br>• Elimination half-life ($t_{1/2,ss}$)<br>• Area under the plasma concentration-time curve at an interval between time 0 (last dose) and t (AUC0-t)

	• Area under the plasma concentration-time curve at an interval between time 0 (last dose) and infinitely great ($AUC0-\infty$)<br>• Oral clearance ($CL/f_{,ss}$)<br>• Renal clearance ($CLr/f_{,ss}$)<br>• Cumulative index: $AUC_{0-24,ss}$ at day 10 / AUC_{0-24} at day 1<br>• Fluctuation index: Fluctuation percentage at steady state = 100 • ($C_{max,ss} – C_{min,ss}$) / $C_{avg,ss}$<br>• Minimum plasma concentration
**Statistical Analysis**	The observed indexes of all subjects are included in the statistical analysis. The data set is divided into full analysis set (FAS) and safety analysis set (SAS).<br>The FAS refers to the set including qualified cases and drop-out cases, but excluding rejected cases.<br>The SAS refers to a set of subjects who are included in the trial and had taken the drug at least once.<br>Given the small number of subjects in this study, the individual case should be examined by professional analysis.<br>For the rejected cases, their records are kept for future reference; those having taken the drug are included in SAS, and the remaining cases would not undergo statistical analysis.<br>Descriptive statistical analysis is mainly adopted for the results of the trial. The mean, standard deviation, median, maximum and minimum values are listed in the measurement data. Frequency (proportion), ratio and 95% confidence interval (CI) are listed in the enumeration data and ranked data. The included, drop-out and rejected cases of each dose group are described in this document. Descriptive statistical analysis is conducted to baseline characteristics of the included cases.<br>**Tolerance assessment:** Descriptive statistical analysis is mainly adopted for tolerance assessment. Adverse events (AEs) and adverse reactions (ARs) occurred in each dose group of the trial are summarized in tables. (ARs are defined as AEs “which are definitely, probably or possibly related to the investigational drug”.) Laboratory findings describe the parameter which is normal before the trial but abnormal after administration, and its relationship with the investigational drug.<br>Vital signs (blood pressure, pulse rate, body temperature and respiration) as well as the mean, standard deviation, median, minimum/maximum values of laboratory indexes of each dose group before

	and after administration will be calculated respectively. The paired *t* test (measurement data) or the nonparametric test (enumeration data and ranked data) is adopted for the comparison before and after administration. Descriptive analysis will be conducted to the change of vital signs and laboratory indexes among dose groups.<br>**PK analysis:**<br>Subjects, who neither materially violate the protocol, nor have a protocol deviation that may influence the PK endpoint variables (AUC, Cmax, etc.), are included in the PK analysis set.<br>The concentration-time curve (CT curve) is plotted based on the plasma concentration of each subject at each time point. Based on the measured plasma concentrations, WinNonlin 6.4 (or other commercial softwares) is used to conduct fitting analysis and calculate PK parameters. The main PK parameters include $t_{1/2}$, Tmax, C_{max}, $AUC_{0-\infty}$, AUC_{0-t}, MRT, cumulative rate R, minimum plasma concentration, cumulative amount excreted (Ae) in urine, cumulative amount excreted (Ae) in feces, cumulative fecal excretion (Fe) and renal clearance (CLr), etc. Analysis of variance (ANOVA) the nonparametric test is conducted to PK parameters of different dose groups. Regression analysis is conducted to validate the linear relationship of PK parameters.<br>**The influence of food on PK**<br>The bioequivalence evaluation is conducted to find out whether food influences the bioavailability of Jaktinib Hydrochloride Tablets. The geometric mean and the 90% CI of C_{max} and AUC_{0-t} of Jaktinib Hydrochloride Tablets after the meal are compared with those under the fasting condition. If the 90% CI falls in 80.00%-125.00% of corresponding PK parameters under the fasting condition, it can be considered that there is no significant difference in main PK parameters between two methods of administration, i.e., food has no influence on PK of Jaktinib Hydrochloride Tablets.<br>After logarithmic transformation of plasma concentrations at each sampling point, ANOVA with 2 x 2 cross-over design is conducted. The nonparametric test is used to calculate Tmax. The significance level of all tests is 5%.<br>The statistical report will be submitted after statistics.

List of Abbreviations

µg	Micrograms
AE	Adverse events
ALT	Alanine transaminase
AST	Aspartate aminotransferase
AUC	Area under the curve
$AUC_{0-\infty}$	Area under the time-concentration curve at an interval between time 0 (after administration) and infinitely great
AUC_{0-t}	Area under the time-concentration curve at an interval between time 0 (after administration) and t
C_{max}	Maximum plasma concentration
CRF	Case report form
CRO	Contract research organization
ECG	Electrocardiography
CFDA	China Food and Drug Administration
GCP	Good Clinical Practice
GLP	Good Laboratory Practice
HBsAg	Hepatitis B surface antigen
HIV	Human immunodeficiency virus
HPLC	High Performance Liquid Chromatography
IB	Investigator’s brochure
ICH	The International Conference on Harmonization of Technical Requirements for Registration of Pharmaceuticals for Human Use
ID_{50}	Half maximal inhibitory concentration
IEC	Independent Ethics Committee
IFN	Interferon
IP	Investigational drug
kg	Kilogram
L	Liter
LC-MS	Liquid chromatography / mass spectrometry
Max	Maximum
MedDRA	Medical Dictionary for Regulatory Activities
mg	Milligram

Min	Minimum
ml	Milliliter
ms	Millisecond
n	Number
NOAEL	No Observed Adverse Effect Level
NAP	Not applicable
NAV	Not available
ND	Not detectable
p.o	per os
RBC	Red blood cells
s	Second
SAE	Serious adverse event
SD	Standard deviation
SDV	Source data verification
SOP	Standard operating procedure
$T_{½}$	Drug's plasma half-life
Tmax	Time of peak concentration
WBC	White blood cells

1 Background

Disease Introduction

Myeloproliferative neoplasms (MPNs) are composed of a group of malignant tumors which mainly include primary myelofibrosis (PMF), chronic myeloid leukemia (CML), essential thrombocythemia (ET), polycythemia vera (PV), chronic neutrophilic leukemia (CNL), chronic eosinophilic leukemia (CEL), NOS (CEL-NOS) and systemic mastocytosis (SM). CML can be diagnosed in the presence of Philadelphia chromosome t(9:22) or BCR/ABL fusion protein produced by the chromosome. The pathogenesis of PMF, ET and PV is related to the mutation of the Janus kinase (referred to as JAK).

Myelofibrosis (MF) is one of MPNs. When MF occurs on its own (*de novo*), it is known as primary myelofibrosis (PMF). It can also develop in individuals who have had other MPNs (known as secondary MF), including post-PV MF (PPV-MF) and post-ET MF (PET-MF).

PMF is a BCR-ABL-negative myeloproliferative disease characterized by MF and extramedullary hematopoiesis, but the degree of MF is not related to the range of extramedullary hematopoiesis. The age of onset is mostly between 50-70 years old. The onset of the disease is slow. In the early stage, the patients have atypical clinical manifestation, and most of them even have no symptom. The middle stage symptom of the disease may include fatigue, weight loss or feeling of abdominal distension caused by splenomegaly. Almost all patients have splenomegaly. The laboratory test results indicate that most patients have different degrees of anemia (generally normocytic normochromic anemias) upon visit, and teardrop-shaped erythrocytes and polychromatic erythrocytes can be seen on mature red blood cells. Reticulocytes may increase slightly between 2%-5%. About 70% of patients present with myelocytes and erythroblasts, as some of characteristic features of the disease. The diagnostic basis of this disease is that marrow cannot be aspirated, and lots of reticular fiber can be observed in the biopsy sample. The chromosome examination shows that half of

patients have chromosome abnormality, but the Philadelphia chromosome is not involved. The overall prognosis is poor with the average survival of 1-5 years. This disease will finally progress to the bone marrow failure or turn to AML.

MF is a rare disease in which pluripotent stem cells undergo malignant clone and proliferation. Among MPNs, patients with MF have the shortest survival and poorest quality of life, with the median survival of about 5 years, and even around 1-2 years for the high-risk patients. MF will finally progress to bone marrow failure or turn to AML. The majority causes of death are hemorrhage, serious infections and heart failure.

Since the role that JAK2 genes play in the onset of BCR-ABL-negative MPNs was reported in 2005 for the first time, JAK2 V617F mutation and MPNs have become important search points. The so-called JAK2 V617F mutation refers to a G to T transverse at nucleotide 1849 in exon 12 of the JAK2 gene. The mutation occurs in the JH2 domain, which causes the valine of JAK2 protein kinase at position 617 in the JH2 domain is miscoded to phenylalanine. WHO has introduced the JAK2 V617F mutation as the main diagnostic criteria for PMF.

The JAK family has 4 members: JAK-1, JAK-2, Tyk2 and JAK-3. The former three are widely distributed in histocyte, while JAK3 can only be found in the marrow and the lymphatic system. The JAK family mediates the signaling of cytokines and growth factors, and plays an important role in hematopoiesis and immunity of human bodies. JAK-1 mainly mediates the signaling of pro-inflammatory cytokines; JAK-2 mainly mediates the signaling of hematopoietic growth factors; JAK-3 mainly mediates the immune response of cytokines; and Tyk2 is involving in signaling of cytokines (e.g., IL-12) mediated by JAK-2 or JAK-3.

Current Treatment Protocols and Clinical Trials

The current treatments for MF include conventional medication, radiotherapy, investigational drugs, and hematopoietic stem cell transplantation (HSCT). The therapeutic strategy can be selected based on the prognosis grouping. The only means that may cure PMF is allogeneic HSCT (allo-HSCT). However, the failure rate of allo-

HSCT is high, and the incidence rate of postoperative complications (chronic graft versus host disease, infection, etc.) and the death rate are high as well. In addition, there is no conclusive evidence confirming that allo-HSCT is related to the prolonged of survival.

Ruxolitinib (Jakavi, INCB018424) is an oral JAK1 and JAK2 inhibitor with high selectivity and oral bioavailability. It has been approved by FDA and EMA to treat adult patients with MF, including PMF, PPV-MF or PET-MF, and to treat PV patients showing an inadequate response to or intolerance to hydroxycarbamide.

The efficacy and safety of Ruxolitinib have been confirmed by Phase I/II clinical trials and two key Phase III clinical trials (COMFORT-I and COMFORT-II). In the Phase I/II trials, 153 patients with MF (including PMF, PPV-MF and PET-MF) were treated by Ruxolitinib, and 65.4% of them were at high risk. Within 3 months after treatment, a ＞50% reduction in spleen volume was achieved in 44% of patients. There was no significant difference in efficacy between patients with and without the JAK2 mutation. In 2011, Incyte published the results of COMFORT-I and COMFORT-II trials in the treatment of MF patients receiving Ruxolitinib. A total of 528 patients participated in the safety and effecacy evaluation of Ruxolitinib. The results showed that in COMFORT-I, a ≥35% reduction in spleen volume was achieved in 41.9% of patients with Ruxolitinib, compared to only 0.7% patients in the placebo group (P<0.001); In COMFORT-II, a ≥35% reduction in spleen volume was achieved in 28% patients with Ruxolitinib at Week 48, compared to 0% patients in the BAT group. The spleen volume of almost all patients treated with Ruxolitinib was reduced at varying degrees, while almost all patients treated with placebo and more than 50% patients treated with BAT experienced an increase in spleen volume. The patient records indicated that Ruxolitinib can effectively reduce spleen volume, regardless of JAK2 mutation, gender, age, IPSS risk category or spleen volume baseline. Compared with the placebo group, MF-related symptoms (including abdominal discomfort, night sweat, pruritus, muscle aches and bone pains) were alleviated more than 50% in most patients

receiving Ruxolitinib, and their quality of life was improved as well. A total of 32.4% of patients treated with Ruxolitinib felt their conditions improved greatly, while most patients in the placebo group felt their condition was either unchanged or worsened. In COMFORT-I, Ruxolitinib significantly reduced death rate compared to the placebo. Ruxolitinib has proven to reduce systemic symptoms and splenomegaly. Ruxolitinib was approved by the US FDA for the treatment of MF in Nov. 2011, by Health Canada in Jul. 2012, and by the EMA in Aug. 2012.

In addition to treating MF, an international, multi-center, randomized, open-label, phase III clinical trial was conducted comparing Ruxolitinib with standard therapy in patients with polycythaemia vera (the RESPONSE trial). The objective of this trial is to investigate the efficacy of Ruxolitinib in preventing thrombosis, reducing the spleen volume, and reducing the risk of transformation from PV to PPV-MF and AML. A total of 232 patients meeting the inclusion criteria were randomly divided to the Ruxolitinib treatment group (110 cases) and the standard treatment group (112 cases). The primary endpoint was the percentage of patients achieving hematocrit control and ≥35% decrease in spleen volume after 32 weeks of treatment as indicated by imaging examination.

Results from the RESPONSE trial showed that 21% of patients in the Ruxolitinib group reached the primary endpoint, compared to only 1% of patients in the standard treatment group (P＜0.001). The percentages of patients achieving hematocrit control in the Ruxolitinib group and the standard treatment group were 60% and 20%, respectively; and 38% and 1% of patients achieved the ≥35% decrease in spleen volume in the above two groups. A complete hematologic remission was achieved in 24% of patients in the Ruxolitinib group and 9% of those in the standard treatment group (P=0.003). 49% (Ruxolitinib) versus 5% (standard treatment) of patients had at least a 50% reduction in the total symptom score at Week 32. The main side effects observed in the Ruxolitinib group were that 2% of patients presented with anemia (Grade 3 or 4) and 5% presented with thrombocytopenia (Grade 3 or 4), while the percentages in the

control group were 0% and 4%, respectively. Herpes zoster infection was reported in 6% of patients in the Ruxolitinib group and 0% of those in the control group (Grade 1 or 2 in all cases). Thrombus was occurred in 1 patient of the Ruxolitinib group, while in 6 patients of the standard treatment group.

Based on the RESPONSE study, in Dec., 2014, the US FDA approved Ruxolitinib for the treatment of patients with polycythemia vera who have had an inadequate response to or are intolerant to hydroxyurea, making Ruxolitinib the first drug approved for the treatment of polycythemia vera.

Momelotinib (CYT387) is a potent JAK1/JAK2 inhibitor developed by Gilead Sciences which is currently undergoing the Phase III clinical trial. The results of the Phase I/II clinical trials for 166 patients have been published. The 166 patients with MF received different doses (100 mg qd - 400 mg qd) of Momelotinib for 9 consecutive months. Three dose groups included majority of patients: 150 mg qd (n=52), 150 mg bid (n=42) and 300 mg qd (n=60), while the other dose groups only had 3 or 6 patients each. Most drug-related AEs were Grade 1 or Grade 2. The most common Grade 3 AE was thrombocytopenia. Thrombocytopenia is common for MF patients due to the inhibited hemopoietic function of bone marrow. The drug-related AEs occurred in the trials are summarized in Table 1.

Table 1. Drug-related AEs of Momelotinib in patients with myelofibrosis at different doses for 9 months.

Adverse Effect (AE) (n=166)	Grade 1	Grade 2	Grade 3	Grade 4
Hematology-Related AE				
Anemia	2%	2%	2%	0%
Leukopenia	2%	< 1%	0%	< 1%
Neutropenia	< 1%	2%	1%	2%
Thrombocytopenia<br>Baseline Platelet > 150 x 10^9/L (n=97)	14%<br>18%	8%<br>1%	17%<br>9%	7%<br>1%

Non-Hematologic AE (≥ 10%)				
Diarrhea	18%	4%	0%	0%
Vertigo	22%	1%	0%	0%
Headache	14%	0%	1%	0%
Nausea	20%	< 1%	0%	0%
Peripheral Neuropathy	25%	2%	0%	0%
Non-Hematologic Lab Abnormality (≥ 5%)				
ALP Elevation	5%	1%	1%	0%
ALT Elevation	9%	3%	2%	0%
Amylase Elevation	7%	2%	0%	0%
AST Elevation	9%	2%	1%	0%
Bilirubin Elevation	7%	2%	0%	0%
Creatinine Elevation	5%	3%	0%	0%
Lipase Elevation	8%	< 1%	4%	0%

Momelotinib showed good preliminary efficacy in patients with myelofibrosis. Responses to Momelotinib in spleen are shown in Table 2. Some disease symptoms were significantly controlled, including fever (100 %), night sweat (57 %), pruritus (52 %), bone pain (33 %), and cough (29 %).

Table 2. Spleen response to Momelotinib in Phase I/II study

Response to Dosage	150 mg qd (n=52)	300 mg qd (n=60)	150 mg bid (n=42)	Number of Cases (n=166)
Evaluable Spleen	47	51	37	145
Spleen Response	32%	39%	38%	37%
Average Reduction in Spleen Volume at Month 6	-36%	-38%	-46%	-38%

Of JAK inhibitors currently under study, Momelotinib is characterized by improving anemia. 59% of patients have experienced improvement of anemia, and

70% of blood transfusion dependent patients (n=33) have experienced a non-transfusion dependent duration of at least 12 weeks (Table 3).

Table 3. Transfusion independence in Phase I/II clinical study of Momelotinib.

Response to Dosage	150 mg qd (n=52)	300 mg qd (n=60)	150 mg bid (n=42)	Total (n=166)
Evaluable Transfusion-Dependent Baseline	24	28	14	68
Transfusion Independence Rate (12 Weeks)	63%	75%	57%	68%
Hemoglobin Elevation of 2 g/dL or Above (8 Weeks)	11%	8%	14%	13%
IWG-MRT Anemia Response Rate	48%	55%	36%	48%

In the extension study of Momelotinib, 58 (39%) patients achieved spleen response meeting IWG-MRT criterion with the median response duration of 785 days. 59 patients (53 %) achieved anemia response with the median maintenance duration of 1,042 days. Grade 3/4 thrombocytopenia was reported in 30% of patients, and "First Dose Effect" of transient dizziness (16 cases [10%]) and hypotension (8 cases [5%]) were also found.

In accordance with the literature published by RA Abdelrahman et. al. from Mayo Clinic, the efficacy were compared and analyzed between 51 myelofibrosis patients enrolled in Ruxolitinib Phase III clinical trial and 60 patients enrolled in Momelotinib Phase I/II clinical trial (Table 4). From the study results, neither group showed complete remission (CR); The Momelotinib group had one case of partial remission (PR), while the Ruxolitinib group had no case of partial remission. According to the 2013 IWG-MRT-ELN efficacy criteria, 57% of patients in Momelotinib group had symptom improvement, while 18% of patients in Ruxolitinib group had symptom improvement. The spleen volume reduction response rates were 42% for Momelotinib group and 15%

for Ruxolitinib group. The anemia response rates were 45% for Momelotinib group and 11% for Ruxolitinib group. The comparative results of efficacy between Ruxolitinib and Momelotinib are listed in Table 4 as follows. One in particular is that although all patient data in this study were from Mayo Clinic, the comparative study was a retrospective analysis rather than a randomized controlled trial of Ruxolitinib and Momelotinib. Thus the study only suggest that Momelotinib may possibly have a better efficacy and a greater symptom improvement rate. Based on results of this study and other Phase I/II clinical trials, Momelotinib is currently undergoing two phase III trials. One is a randomized, open-label trial comparing Momelotinib with best available therapy, and the other is a double-blind, double dummy, head-to-head comparative study of Momelotinib and Ruxolitinib. The two Phase III trials have no released results yet.

Table 4. Efficacy comparison of Momelotinib and Ruxolitinib (as per 2013 IWG-MRT criteria).

Efficacy Endpoints	Momelotinib Group (n=60)	Ruxolitinib Group (n=51)
CR, n (%)	0 (0%)	0 (0%)
PR, n (%)	1 (1.7%)	0 (0%)
Clinical Improvement (CI), n (%)	34 (57%)	9 (18%)
Anemia Response [n (responses; %)]	42 (19; 45%)	19 (2; 11%)
Anemia Response in Transfusion-Dependent Patients [n (responses; %)]	32 (17; 53%)	12 (2; 17%)
Spleen Response [Evaluables (responses; %)]	57 (24; 42%)	46 (7; 15%)
Median Duration of Treatment, Month (Range)	26 (3–47+)	12 (1–68)

Development of Jaktinib Hydrochloride Tablet

Background

Jaktinib hydrochloride tablet, with its substance being Jaktinib dihydrochloride monohydrate (Code ZG0128) and its active substance being dihydrochloride monohydrate of Jaktinib (Code ZG0163), is a JAK inhibitor-based, small molecule, oral, Category 1 new drug developed by Suzhou Zelgen Biopharmaceuticals Co., Ltd.. The pharmacological mechanism of Jaktinib hydrochloride has been clarified by in vivo and in vitro pharmacodynamic studies: Jaktinib is a kinase inhibitor that inhibits JAK1 and JAK2 in non-receptor tyrosine kinase family Janus, and inhibits FLT3 (FMS-like tyrosinase 3) and c-Kit in receptor tyrosine kinase Class III (RTK III). JAKV617F transgenic mice and JAK2V617F bone marrow transplanted mice with pathological phenotype of myeloproliferative neoplasms were orally/intragastrically administered ZG0128. The pathological characteristics of bone marrow proliferative tumors were significantly improved by Jaktinib, including spleen volume reduction, inhibiting abnormal proliferation and blocking myelofibrosis. Jaktinib also presented good pre-clinical pharmacokinetics. Pre-clinical toxicological studies indicated that apart from the toxic and side effects associated with pharmacological effects, Jaktinib has clear toxic target organs and controllable safety.

General Information

Product Name

Generic Name: Jaktinib Dihydrochloride Monohydrate

Table 5. General physicochemical properties of Jaktinib hydrochloride tablet

Items	Description
Appearance	Yellow to orange crystalline powder; Odorless; Bitter taste
Solubility	This product is freely soluble in dimethyl sulfoxide or *N*-methylpyrrolidone; soluble in *N*, *N*-dimethyl formamide;<br>slightly soluble in methanol or 0.1 mol/L hydrochloric acid; very slightly soluble in ethanol;<br>practically insoluble in acetone, isopropanol, glacial acetic acid, acetonitrile, dioxane, tert-butyl methyl ether or water
Hygroscopicity	Slightly hygroscopic
Crystal Form	Jaktinib dihydrochloride monohydrate is a crystalline solid containing two molecules of hydrogen chloride and one molecule of water of crystallization, with its crystal form being polymorph II

Basis

Existing pre-clinical data, including toxicology, pharmacodynamics and pharmacokinetics of Jaktinib hydrochloride, suggest that the Jaktinib hydrochloride tablet is ready for Phase I trials. The clinical study intended to carry out following trials in healthy subjects: 1) Single ascending dose study of Jaktinib hydrochloride tablet by for oral administration, to investigate the safety and tolerability, and to find any potential dose-limiting toxicity (DLT) and the maximum tolerated dose (MTD); 2) Multiple ascending dose study of Jaktinib hydrochloride tablet by for oral administration on the basis of single ascending dose study, to investigate the safety and tolerability, and to find any potential DLT and the MTD; 3) Food effect and metabolism study based on the multiple ascending dose study and the observed MTD; 4) Pharmacokinetics of single and multiple oral administration of Jaktinib hydrochloride tablet in human body.

Selection of Control

Placebo is selected as the control to evaluate the tolerability of Jaktinib hydrochloride tablet in healthy subjects.

Specification

The specification of Jaktinib hydrochloride tablet produced by WuXi AppTec and provided by Suzhou Zelgen Biopharmaceuticals Co., Ltd. is 50 mg/tablet.

Content of Trial

Objectives

Primary Objective

To evaluate the safety and tolerability after single and multiple oral administration of Jaktinib hydrochloride tablet at different doses in healthy subjects, and to find any potential DLT and the MTD.

Secondary Objectives

To evaluate the PK parameters in humans after single and multiple oral administration of Jaktinib hydrochloride tablets;

To observe the effects of high-fat diet on the metabolism of Jaktinib hydrochloride tablets;

Analysis of drug metabolites and metabolism study.

Trial Phase

Phase Ia.

Trial Design

Study Design

This is a single center, randomized, double-blind, placebo-controlled, multiple-dose, single and multiple ascending dose study, as well as food effect study regarding pharmacokinetics and drug metabolism. The aim is to evaluate the effects of tolerability, PK characteristics, drug metabolism, and food of single- and multiple-dose Jaktinib hydrochloride tablets on PK in healthy subjects.

Single Ascending Dose (SAD)

The subjects are randomized into **8** dose groups: 25 mg (group 1), 50 mg (group 2), 100 mg (group 3), 150 mg (group 4), 200 mg (group 5), 250 mg (group 6), 300 mg (group 7), and 400 mg (group 8). 8 subjects was enrolled in each group, of which **6**receive the investigational drug and **2** receive the placebo, with a male:female ratio close to 1:1. Each group will receive a single dose under the fasting condition, and the tolerability will be assessed on D3 by lab test. Subjects of different dose groups are enrolled sequentially. The next dose group is tested only if the previous group is well tolerated.

Multiple Ascending Dose (MAD)

The subjects are randomized into 5 dose groups: 100 mg q24h (group 9), 150 mg q24h (group 10), 100 mg q12h (group 11), 200 mg q24h (group 12), 150 mg q12h

(group 13). The dose selection of each group may be adjusted based on the results of SAD study.

After completing the tolerability assessment for the single-dose 100 mg group, if tolerated, the tolerability assessment for the multiple-dose 100 mg q24h group can be started.

Each group will enroll 10 subjects, 8 receiving the investigational drug and 2 receiving placebo. The male to female ratio should be close to 1:1. Each group will receive 10 or 19 doses under the fasting condition, and undergo tolerability assessment on D4, D7, and D12. Subjects of different dose groups are enrolled sequentially. The next dose group is tested only if the previous group is well tolerated.

Effects of Food on PK and Drug Metabolism

This is a randomized, two-period, crossover study evaluating the food effects on PK and drug metabolism. Totally 12 healthy subjects are randomized into group A and group B, with 6 subjects in each group and a male to female ratio close to 1:1. Subjects will receive 200 mg Jaktinib hydrochloride tablets or the MTD of Jaktinib hydrochloride tablets observed during the MAD study. The specific dose is selected together by the investigators and the sponsor.

Group A will receive Jaktinib hydrochloride tablets under the fasting condition (Period 1), and after washout, the subject will receive Jaktinib hydrochloride tablets after the meal (Period 2). Group B will receive Jaktinib hydrochloride tablets after the meal (Period 1), and after washout, the subject will receive Jaktinib hydrochloride tablets under the fasting condition (Period 2). The washout period is 5 days between the two periods. PK urine and feces will be collected for Group A in Period 1 under the fasting conditions. The study is completed after the tolerability assessment on D8.

Studies of food effect on pharmacokinetics and drug metabolism is performed based on the tolerated dose in the single administration study.

Subjects may withdraw from the trial if there are no clinically significant abnormalities in laboratory tests and clinical observation. If the abnormality has clinical

significance, the investigator may determine to continue follow-up until the subject recovers to normal or stable, or is lost to follow-up.

During the trial, blood samples for PK analysis are collected at scheduled time points according to the protocol. Since Jaktinib has never been used in humans, no human PK parameters can be used as reference. Therefore, sampling time points may be finely adjusted based on the PK results of first set.

Tolerability Criteria

MTD: According to NCI-CTCAE v4.03 evaluation criteria, dose-limiting toxicity (DLT) is defined as drug-related grade 3 non-hematologic or grade 4 hematologic adverse events experienced by more than 1/3 of subjects in a dose group or experience drug-related serious adverse events occurring in 1 subject of a dose group, that suggests an evident intolerance. The dose of the previous group is defined as the maximum tolerated dose (MTD).

Dose Ascending Flow Chart

Part 1: SAD

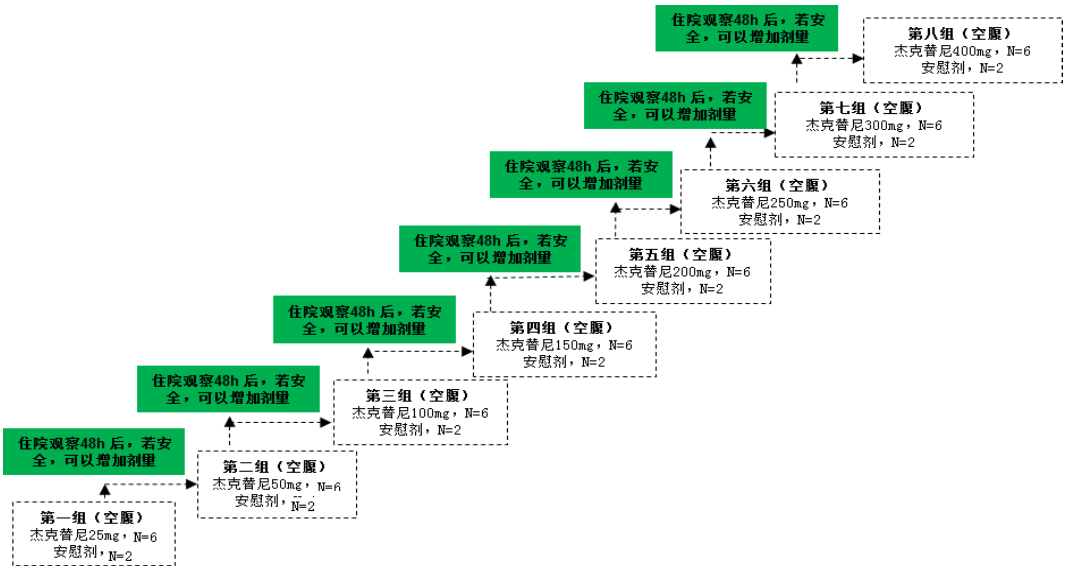

Part 2: MAD

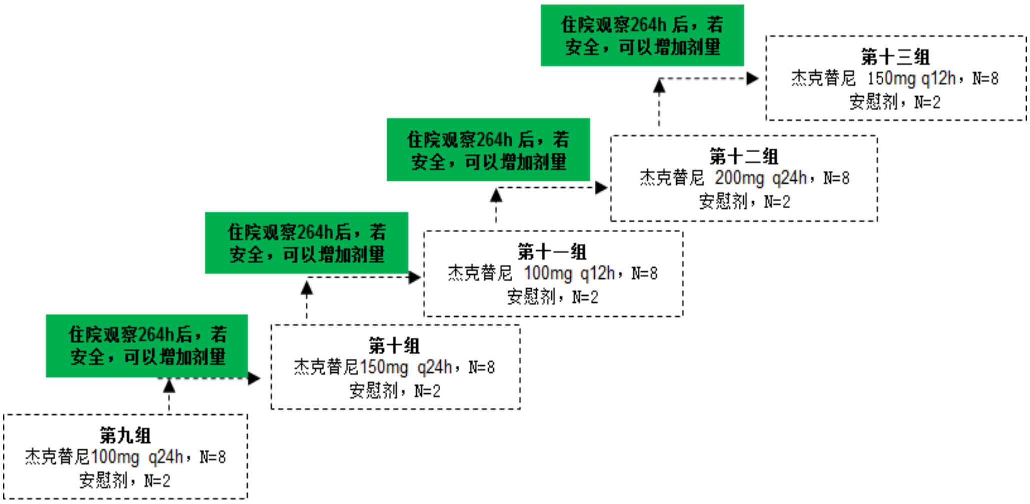

Dose Ascending

From Group 1 to Group 8, subjects are given drugs at 25 mg (50 mg/tablet, 1/2 tablet/day, qd), 50 mg (50 mg/tablet, 1 tablet/day, qd), 100 mg (50 mg/tablet, 2 tablets/day, qd), 150 mg (50 mg/tablet, 3 tablets/day, qd), 200 mg (50 mg/tablet, 4 tablets/day, qd), 250 mg (50 mg/tablet, 5 tablets/day, qd), 300 mg (50 mg/tablet, 6 tablets/day, qd) and 400 mg (50 mg/tablet, 8 tablets/day, qd). Among the 8 subjects in each group, 6 subjects are given the investigational drug and 2 subjects are given placebo. The drug should be orally administered once with 240 mL of warm water on under fasting condition in the morning. The subjects are hospitalized for observation, and the tolerability assessment is conducted 48 hours after administration (D3). In case of well tolerated dose, the trial would proceed to the next dose in sequence until the termination criterion is met. The trial may proceed to the next dosage group only if the previous tolerability assessment has been completed and a well tolerability is indicated. Refer to the SAD flow chart for details. If the highest dose group still shows tolerability, but the maximum dose designed for the trial have been reached, the trial can also be terminated.

From Group 9 to Group 13, subjects are given drugs at 100 mg q24h, 150 mg q24h, 100 mg q12h, 200 mg q24h and 150 mg q12h. Among the 10 subjects in each group, 8 subjects are given the investigational drug, and 2 are given placebo. The drugs are orally

administered with 240 mL of warm water under fasting condition in the morning or 2 h after supper with an interval of 12 h from previous dose. The drugs will be administered 10 times for q24h, and 19 times for q12h. The subjects are hospitalized for observation, and the tolerability assessment is conducted on D4, D7, and D12 during the hospital stay.

After the tolerability evaluation of the 100 mg single dose group was completed with tolerance indicated, the tolerability evaluation of the 100 mg q24h multiple administration may be carried out. In case of well tolerated dose, the trial would proceed to the next dose in sequence. The trial may proceed to the next dosage group only if the previous tolerability assessment has been completed and a well tolerability is indicated. The dose will escalate as shown in the MAD flow chart until the termination criterion is met. If the highest dose group still shows tolerability, but the maximum dose designed for the trial have been reached, the trial can also be terminated.

Studies of food effect on pharmacokinetics and drug metabolism is performed based on the tolerated dose in the single administration study.

Dosage Regimen

64 healthy subjects will be enrolled and randomized into 8 dosage groups (25 mg, 50 mg, 100 mg, 150 mg, 200 mg, 250 mg, 300 mg and 400 mg) for the single administration study.

50 healthy subjects will be enrolled and randomized into 5 dosage groups (100 mg q24h, 150 mg q24h, 100 mg q12h, 200 mg q24h and 150 mg q12h) for the multiple administration study.

12 healthy subjects will be enrolled for the study of food effects on PK and drug metabolism at 200 mg or the MTD observed in multiple administration study.

For each group in single administration study (group 1 to 8), 8 subjects will be enrolled, with 6 receiving the investigational drug and 2 receiving placebo. The male to female ratio should be close to 1:1. One dose will be given for each group with 240 mL of warm water under fasting condition in the morning. The lunch is served 4 hours

after administration, and the dinner is served 10 hours after administration. Refer to Table 6 for the dose ascending in single administration study.

For each group in multiple administration study (group 9 to 13), 10 subjects will be enrolled, with **8** receiving the investigational drug and **2** receiving placebo. The male to female ratio should be close to 1:1. Drugs will be given 10 to 19 times for each dosage group, as detailed in Table 7. Drugs are given under fasting condition with 240 mL of warm water on D1 and D10 morning, and the breakfasts are served 0.5 h after the administration on D2-D9. The lunches are served 4 hours postdose, and the dinners are served 10 hours after administration. q12h dosing means drug is given 2 hours after supper, at 12 hours intervals from the last dose.

Table 6. Dose escalation protocol for the single administration study (64 cases).

Group	Group 1	Group 2	Group 3	Group 4	Group 5	Group 6	Group 7	Group 8
Dose (mg)	25	50	100	150	200	250	300	400
Dose (Number of Doses)	1	1	1	1	1	1	1	1
Dosing Time	D1	D1	D1	D1	D1	D1	D1	D1
Number of Cases in Each Test Group	6	6	6	6	6	6	6	6
Number of Cases in Placebo Group	2	2	2	2	2	2	2	2
Total Number of Cases	8	8	8	8	8	8	8	8

Table 7. Dose escalation protocol for the multiple administration study (50 cases).

Group	Group 9	Group 10	Group 11	Group 12	Group 13
Dose (mg)	100 mg q24h	150 mg q24h	100 mg q12h	200 mg q24h	150 mg q12h
Dose (Number of Doses)	10	10	19	10	19

Dosing Time	D1-D10	D1-D10	D1-D10	D1-D10	D1-D10
Number of Cases in Each Test Group	8	8	8	8	8
Number of Cases in each Placebo Group	2	2	2	2	2
Total Number of Cases	10	10	10	10	10

Under the fasting condition or after the meal, 12 subjects, who should participate in the study of food effects on PK and drug metabolism, received 200 mg Jaktinib hydrochloride tablets or the MTD of Jaktinib hydrochloride tablets observed during the multiple administration study, which was determined by both the investigators and the sponsor according to the results of the multiple administration study and the intended dose for clinical application. The drug should be orally administered twice, as shown in Table 8. After an overnight fast of at least 10 hours, subjects, who should receive the drug after the meal, was served a standard meal 30 min before the administration. The eating speed should be controlled and the meal was finished before dosing. The investigational drug was given according to the randomization table. After an overnight fast of at least 10 hours, subjects, who should receive the drug under fasting conditions, was also administered the investigational drug according to the randomization table.

Table 8. Dosage regimen for crossover study of food effects on pharmacokinetics.

Dosing Period	Group A (n=6)	Group B (n=6)
Period 1 (D1-D6)	Fasting Condition	Postprandial Condition
Period 2 (D6-D8)	Postprandial Condition	Fasting Condition

Dose Selection

Animal studies showed that, in a long-term toxicity study of 28-day continuous drug administration, the no-observed-adverse-effect-level (NOAEL) in Beagle dogs was 18.3 mg/kg. However, in a long-term toxicity study of 26-week continuous drug

administration in rats, the NOAEL was 30 mg/kg. The human equivalent doses (HED) for the above tests were 9 mg/kg and 5 mg/kg, respectively (Table 9). According to the "Guidance for Estimating the Maximum Recommended Dose in Initial Clinical Trials for Therapeutics in Adult Healthy Volunteers" issued by CFDA and a safety factor of 10, the maximum safe starting doses for the first dose in human were calculated as about 50 mg and 30 mg, respectively.

The single starting dose of this study was set to 25 mg, while the highest dose was set to 400 mg. The 400 mg dose would not be further increased even it was tolerated.

Table 9.Estimate the MRST of Jaktinib using animal toxicity test.

Animal Test Type	Animal	Dosage (mg/kg/day) Route	HNSTD /NOAEL (mg/kg/day)	HED (mg/kg/day)	MRSD (mg/day) [a]
26-Week Toxicity Test in Beagle	Rats	4/Oral<br>12/Oral<br>30/Oral	NOAEL: 30	5	30
28-Day Toxicity Test in Beagle Dogs	Dogs	18.3/oral gavage<br>61.0/oral gavage<br>196.2/oral gavage	NOAEL: 18.3	9	54

a The safety factor was 10, while the human body weight was calculated as 60 kg.

Randomization and Double-blind

This is a randomized and double-blind study.

After the subjects signed the informed consent form, they participated in the screening and physical examination. The subjects who passed the physical examination could be admitted to the Phase I clinical center within the prescribed time. For subjects showing no abnormal results in the admission examination, they were assigned a "randomization number" according to the requirements of randomization in the protocol. The investigators distributed investigational drugs to subjects based on their randomization numbers.

In the single ascending dose trial, 8 subjects were allocated into each group. All subjects were randomly assigned into the group of Jaktinib hydrochloride tablets or the placebo group in a 3:1 ratio. For the multiple ascending dose trial, each dose group contained 10 subjects, who were randomly assigned into the group of Jaktinib hydrochloride tablets or the placebo group in a 4:1 ratio. For the study on food effects, 12 subjects were randomly assigned into a fasting-standard meal group and a standard meal-fasting group, with 6 subjects in each group.

The final version of the randomization table in each trial was generated by an unblinded statistician, who was unrelated to this trial, using the program of block randomization in SAS 9.4 software. The unblinded statistician sent the final randomization table to the sponsor-designated drug packaging personnel, who did not participate in this trial, to package and prepare the drugs according to the randomization table. In addition, the unblinded statistician would prepare two sealed envelopes, with each envelope containing the enrollment number of the subjects and the corresponding investigational drugs. One envelope was kept by the personnel of the sponsor who were not related to the study, and the other envelope was kept by the study site.

Single ascending dose: The randomization numbers for a total of 8 dose groups were generated. For subjects who met the inclusion criteria, male subjects were enrolled into the first half of each group and female subjects were enrolled into the second half of each group, so that the male to female ratio in each group was close to 1:1. The investigators would distribute the investigational drugs to the subjects based on the corresponding randomization numbers. The randomization numbers of the subjects in each dose group were expressed in three digits as shown below:

25 mg: 101, 102, 103, 104, 105, 106, 107, 108;

50 mg: 201, 202, 203, 204, 205, 206, 207, 208;

……;

300 mg: 701, 702, 703, 704, 705, 706, 707, 708;

400 mg: 801, 802, 803, 804, 805, 806, 807, 808.

Multiple ascending dose: The randomization numbers for a total of 5 dose groups were generated. For subjects who met the inclusion criteria, male subjects were assigned to the first half of each group and female subjects were assigned to the second half of each group, so that the male to female ratio in each group was close to 1:1. The investigators would distribute the investigational drugs to the subjects based on the corresponding randomization numbers. The randomization numbers of the subjects in each dose group were expressed in four digits as shown below:

100 mg q24h dose group: 1001, 1002, 1003 ... 1010;

150 mg q24h dose group: 2001, 2002, 2003 ... 2010;

100 mg q12h dose group: 3001, 3002, 3003 ... 3010;

200 mg q24h dose group: 4001, 4002, 4003 ... 4010;

150 mg q12h dose group: 5001, 5002, 5003 ... 5010.

Food effects study: A total of 12 randomization numbers were generated for one group. For subjects who met the inclusion criteria, they were assigned into two groups at an equal ratio: the subjects in one group received the drug under fasting conditions, while the subjects in the other group received the drug after the standard meal. Among the subjects, half were males and half were female, so that the male to female ratio in the group was close to 1:1. The investigators would distribute the investigational drugs to the subjects based on the corresponding randomization numbers. The randomization numbers were expressed in four digits starting with number 9, as shown below:

9001, 9002, 9003 ... 9011, 9012.

Blinding: The double-blind means that none of the subjects, investigators, and monitors know the distribution information of the investigational drugs. The investigational drug and placebo were provided by the sponsor or the sponsor-designated organization to ensure that the investigational drug and placebo were similar in appearance and weight. The investigational drug and placebo of each test group were blinded by a statistician, while the statistical analysts randomly assigned the subjects into the test group and the placebo group. After the completion of blinding, the blind

code should be prepared in duplicate and sealed in two separate envelopes, which were kept by the responsible unit of the clinical study and the applicant of drug registration, respectively. In the event of SAE or other emergencies, the research physicians could open the envelope to learn about the drug used by the patient for rescue. This process is called emergency unblinding. After the dose ascending in each group, if the tolerability assessment considered that the subject was intolerant, emergency unblinding was also performed to determine whether to continue the dose ascending.

Level of blinding: This trial used a double-blind technique, i.e., both the research physicians and the subjects were unable to determine the drug received by the subjects.

The details are as follows:

1) According to the GCP regulations on the administration of investigational drugs, the investigational drugs were uniformly packaged and labeled, and indicated for clinical trial use. The packages in both test group and placebo group were completely the same. The blinding and packaging of the drugs were carried out by relevant personnel. The drugs used by each subject were individually packaged. The whole process should be checked by a designated person and recorded in detail.

2) After the investigational drugs were distributed to the study site, they shall be kept by the designated personnel of the study site.

Emergency envelopes and blind code: An emergency envelope was prepared for each subject before the start of the trial. The drug number of the subject should be marked on the envelope. The letter enclosed in the envelope should indicate the group of the subject for emergency unblinding if needed. All investigational drugs would be distributed to the study site along with the emergency envelopes labeled with corresponding drug numbers.

The blinding process should have a written record, which should be signed by all participating personnel. The blind code must be sealed on the spot after the drugs were aliquoted. The blind code should be kept in two places by the designated staff at the study site and the sponsor, respectively. The emergency unblinding envelopes should

be sent to the study site along with the investigational drugs and be kept by the designated staff at the study site until the end of the trial.

Emergency unblinding: When emergency unblinding was necessary, the investigators should ask the director of the study site. After obtaining the consent from the director, the emergency unblinding envelope could be opened to learn about the drug used. Notify the relevant personnel of the sponsor within 24 h after the unblinding and explain the reasons for unblinding. Emergency unblinding can be considered in the following situations, including but not limited to:

(1) A patient experiences a serious adverse event, which is considered to be possibly related to the investigational drug or control drug;

(2) A patient experiences serious complications.

Regulations for unblinding: This trial adopted one-time unblinding. After the database was locked, the unblinding was carried out by the study site and the sponsor. The relevant personnel responsible for keeping the blind code would give the grouping information of the trial to the statistical department for statistical analysis.

Study Indicators and Detection Time Points

Tolerability Study

Single Ascending Dose (SAD)

Laboratory tests: screening period, and D3;

Including routine blood test, routine urinalysis, coagulation tests, blood biochemistry, and stool routine (including fecal occult blood). The specific indicators of the examinations were shown in Appendix 2, while the calculation formula of creatinine clearance was shown in Appendix 3.

Note: If the urinary red blood cell count was abnormal and clinically significant, the investigators should decide whether a three-glass test was needed. If the urine protein was positive and clinically significant, the investigators should decide whether a quantitative test of 24 h urine protein was needed.

Symptoms and physical examinations: screening period, D3;

Physical examinations performed by physicians included the examinations of skin, mucous membranes, lymph nodes, head, neck, breast, abdomen, and spine/extremities. Additional physical examinations could be performed if deemed necessary by the investigators.

12-lead ECG: screening period, and D3;

Subjects should rest for at least 5 min before ECG assessment.

Vital signs: screening period, hospital admission, within 1 h before the first dose, and 2 h, 6 h, 24 h, and 48 h after the start of dosing;

The examinations of vital signs included blood pressure, pulse, respiration and body temperature. Subjects should rest for at least 5 min before vital sign assessment.

Chest X-ray and abdominal ultrasound: screening period;

Multiple Ascending Dose (MAD)

Laboratory tests: routine blood test, routine urinalysis, coagulation tests, blood biochemistry, and stool routine (including fecal occult blood) were carried out during the screening period and on D4, D7, and D12; only routine blood test was carried out on D10. The specific indicators of the examinations were shown in Appendix 2, while the calculation formula of creatinine clearance was shown in Appendix 3.

Note: If the urinary red blood cell count was abnormal and clinically significant, the investigators should decide whether a three-glass test was needed. If the urine protein was positive and clinically significant, the investigators should decide whether a quantitative test of 24 h urine protein was needed.

Symptoms and physical examination: screening period, D4, D7, and D12;

Physical examinations performed by physicians included the examinations of skin, mucous membranes, lymph nodes, head, neck, breast, abdomen, and spine/extremities. Additional physical examinations could be performed if deemed necessary by the investigators.

12-lead ECG: screening period, D4, D7, and D12;

Subjects should rest for at least 5 min before ECG assessment.

Vital signs: screening period, hospital admission, within 1 h before the first dose, and

2 h and 6 h after the start of dosing; within 1 h before dosing on D2, D3, D4 and D7; within 1 h before the last dose, and 2 h, 6 h, 24 h, and 48 h after the start of dosing; The examinations of vital signs included blood pressure, pulse, respiration and body temperature. Subjects should rest for at least 5 min before vital sign assessment.

Chest X-ray and abdominal ultrasound: screening period;

Effects of Food on PK and Drug Metabolism

Laboratory tests: screening period, and D8;

Including routine blood test, routine urinalysis, coagulation tests, blood biochemistry, and stool routine (including fecal occult blood). The specific indicators of the examinations were shown in Appendix 2, while the calculation formula of creatinine clearance was shown in Appendix 3.

Note: If the urinary red blood cell count was abnormal and clinically significant, the investigators should decide whether a three-glass test was needed. If the urine protein was positive and clinically significant, the investigators should decide whether a quantitative test of 24 h urine protein was needed.

Symptoms and physical examinations: screening period, D8;

Physical examinations performed by physicians included the examinations of skin, mucous membranes, lymph nodes, head, neck, breast, abdomen, and spine/extremities. Additional physical examinations could be performed if deemed necessary by the investigators.

12-lead ECG: screening period, and D8;

Subjects should rest for at least 5 min before ECG assessment.

Vital signs: screening period, hospital admission. In period 1: within 1 h before dosing, and 2 h, 6 h, 24 h, 48 h, 72 h and 120 h after the start of dosing. In period 2: 2 h, 6 h, 24 h, and 48 h after dosing.

The examinations of vital signs included blood pressure, pulse, respiration and body temperature. Subjects should rest for at least 5 min before vital sign assessment.

Chest X-ray and abdominal ultrasound: screening period;

Pharmacokinetics Study

PK Blood Sampling

Single Ascending Dose (SAD):

Samples were collected within 30 min before dosing, and 0.25 h, 0.5 h, 1 h, 1.5 h, 2 h, 3 h, 4 h, 5 h, 6 h, 8 h, 12 h, 14 h, 24 h, 36 h and 48 h after dosing. 4 mL of samples were collected at each time point.

Multiple Ascending Dose (MAD):

Samples were collected as following: on D1, within 30 min before dosing, and 0.25 h, 0.5 h, 1 h, 1.5 h, 2 h, 3 h, 4 h, 5 h, 6 h, 8 h, and 12 h after dosing; On day 2, 3, 4, 6 and 7, within 30 min before dosing in the morning. On day 10, within 30 min before dosing, and 0.25 h, 0.5 h, 1 h, 1.5 h, 2 h, 3 h, 4 h, 5 h, 6 h, 8 h, and 12 h 24 h, 36 h and 48 h after dosing. 4 mL of samples were collected at each time point.

Effects of food on PK and drug metabolism:

Group A in Period 1 under the fasting conditions (D1):

PK blood samples: collected within 30 minutes before dosing, 0.25 h, 0.5 h, 1 h, 1.5 h, 2 h, 3 h, 4 h, 5 h, 6 h, 8 h, 12 h, 14 h, 24 h, 36 h, 48 h, 72 h, 96 h, and 120 h after dosing;

Urine: Collected before dosing and 0-6, 6-12, 12-24, 24-48, 48-72, 72-96, and 96-120 h after dosing;

Feces: collected if the subjects had bowel movement after dosing (0-120 h)

Group A in Period 2 after standard meal (D6) and Group B in two periods (D1, D6):

PK blood samples: collected within 30 minutes before dosing, 0.25 h, 0.5 h, 1 h, 1.5 h, 2 h, 3 h, 4 h, 5 h, 6 h, 8 h, 12 h, 14 h, 24 h, 36 h, and 48 h after dosing

For Group 1 subjects participating in the single-dose clinical study, after the results of plasma concentration then became available, the sampling points might be finely adjusted.

Blood Collection and Processing:

Subjects were inserted an indwelling needle in the forearm vein. Subsequently, 4.0 mL of PK blood sample were collected from each subject using a K2-EDTA vacutainer, which was then inverted and mixed for 5 times. The vacutainers were placed in an ice bath box, and within 30 min, centrifuged at 3000 rpm and 2-8 ℃ for 10 min. After the centrifugation was completed, the upper plasma was transferred into two labeled cryogenic vials (1.0 mL of the plasma sample was stored in one of the vials for PK test, while the other vial was used as a backup). After capping the vials tightly, the vials were placed into a cryogenic box and stored at -70 ℃ to -90 ℃ in a vertical position. The time used from sample collection to storage should not be more than 1 h.

The label included the protocol number, the time point of sample collection, and the randomization number, which should be longitudinally affixed to the room temperature vials and the cryogenic vial. The exact date and time of sampling should be recorded in the original record.

In this study, pre-dose blood samples were collected within 30 min prior to dosing, and the post-dose blood samples should be collected within the allowed time frame. Allowable time deviation during blood collection: When the time interval of sampling points was less than or equal to 1 h, the time deviation should be within ± 5%; when the time interval of sampling points was greater than 1 h, the time deviation should be within ± 2.5%. See Appendix 1 for specific requirements for the sampling time.

Blood collection tube: 4 mL vacuum anticoagulation tube (K_2-EDTA).

Inspection agency: Suzhou Haike Pharmaceutical Technology Co., Ltd.

Inspection method: Jaktinib hydrochloride and its metabolites (ZG0244 and ZG0245) in plasma were quantified by the LC-MS/MS method.

Collection of PK Urine

Collection time period:

Effects of Food on PK and Drug Metabolism

The urine from the subjects in Group A was collected in period 1 under the fasting conditions in the following time: prior to dosing, 0-6 h, 6-12 h, 12-24 h, 24-48 h, 48-72 h, 72-96 h, and 96-120 h.

Urine collection and processing: After the urine was collected during each time period, the total volume of urine was recorded. Subsequently, the urine was transferred into two urine tubes, respectively, with a total volume of about 5 mL/tube. After aliquoting, the urine tubes were quickly moved to -70 ℃ to -90 ℃ for cryopreservation. At the end of the study, one sample was shipped to the testing center for inspection, while the other one was retained in the Phase I clinical trial laboratory.

Inspection unit: Suzhou Haike Pharmaceutical Solutions Co., Ltd.

Inspection method: Jaktinib hydrochloride and its metabolites (ZG0244, ZG0243, ZG0245, and ZG0262) in urine were quantified by LC-MS/MS.

Note: The deviation in the urine collection time should be within ±15 min for each collection period. For example, if the drug was given at 7:00, the collection time for the 0-6 h urine could be within 13:00 ± 15 min;

Collection of PK Feces

Collection time period: For the study on drug metabolism and the food effects on the pharmacokinetics, the subjects in Group A were given the first dose under fasting conditions. Subsequently, the feces was collected (0-120 h) if the subjects had bowel movement.

Feces Collection and Processing: For the method of feces collection and processing, please refer to the SOP for feces processing provided by the sponsor.

Inspection agency: Suzhou Haike Pharmaceutical Technology Co., Ltd.

Inspection method: Jaktinib hydrochloride and its metabolites (ZG0244, ZG0243, ZG0245, and ZG0262) in feces were quantified by LC-MS/MS.

2 Justification of Trial Design

Overall Design

The overall design of this clinical study was a single-center, randomized, double-blind, placebo-controlled, and escalating single- and multiple-dose study evaluating the effect of food on the pharmacokinetic and drug metabolism.

Overview of Preclinical Study

A comprehensive and extensive preclinical study of ZG0128 has been conducted to elucidate the preclinical pharmacology, pharmacokinetics and toxicological characteristics of ZG0128. The following are several compound codes used in preclinical studies.

ZG0128	ZG0163 dihydrochloride monohydrate	ZG0163 dihydrochloride monohydrate
ZG0163	Jaktinib	Jaktinib
ZG0192	ZG0164 dihydrochloride monohydrate or momelotinib dihydrochloride monohydrate	ZG0164 dihydrochloride monohydrate or momelotinib dihydrochloride monohydrate
ZG0164	Momelotinib	Momelotinib
ZG0178	Ruxolitinib	Ruxolitinib

In the in vitro pharmacology tests, the following cell models which were closest to the myelodysplastic syndrome were used: Ba/F3-EpoR-JAK2V617F cells, wild-type Ba/F3-EpoR-JAK2 cells, Ba/F3-EpoR mother cells, J53Z1 cells harboring the JAK2V617F mutation, 8 HCD-57 cell lines stably transfected with oncogenic mutations JAK2V617F, MPLW515L, KITD816V, NPM-ALK, FLT3-ITD, FLT3D835H, FLT3D835Y and KRasG12V, respectively, and the primary blood cells in polycythemia patients with positive JAK2V617F. The in vitro inhibition and the extent of JAK kinase inhibition, as well as the inhibition of abnormal activity of the JAK-STAT signaling pathway in above cells by ZG0128 or its active ingredient ZG0163 were evaluated at the biochemical and cellular levels. In the in vivo pharmacology tests, two animal models with the pathological phenotypes of

myeloproliferative neoplasms, i.e., JAK2V617F bone marrow transplanted mice and JAKV617F transgenic mice, were used to evaluate the effects of ZG0128 in the reduction of blood cell (red blood cells, platelets and white blood cells) counts, reduction in spleen volume, and myelofibrosis blocking. In addition, the in vitro and in vivo pharmacodynamic mechanisms and degree of effects of ZG0128 (or its active ingredient ZG0163) and its positive control drugs Momelotinib and Ruxolitinib were also compared at the cellular level and in animal models.

In the pharmacokinetic study, according to the relevant requirements for the non-clinical pharmacokinetic study of national Class 1 new drug, the absorption, distribution, metabolism and excretion processes and characteristics of ZG0128 in ICR mice, rats and Beagle dogs were studied to obtain the basic pharmacokinetic parameters and to evaluate the species differences during the ZG0128 metabolisms in liver microsomes. In addition, the enzyme phenotypes of main metabolic pathways were identified, while the inhibition and induction of major CYP450 enzymes by ZG0128 were evaluated. The permeability of ZG0128 in the Caco-2 cell model was also studied. Furthermore, in vitro and in vivo pharmacokinetic properties between ZG0128 and control drugs were compared in terms of in vivo pharmacokinetics and in vitro metabolic stability, so as to provide a basis for the clinical development of ZG0128.

In safety pharmacology study, the effects of ZG0128 on the respiratory system, status and behavior of the central nervous system, cardiovascular system, and heart were evaluated according to the GLP standard.

In toxicology study, according to the relevant requirements for the non-clinical toxicology study of national Class 1 new drug and the GLP standard, a toxicity test by single oral administration, a toxicity test by multiple oral administration (28 days), toxicity tests on fertility and early embryonic development, and genotoxicity tests were carried out. In addition, in order to more comprehensively evaluate the toxicological characteristics of ZG0128, a 26-week toxicity test in Wistar rats byoral administration

has been completed. The ongoing toxicity tests include a long-term 39-week toxicity test in Beagle dogs by oral administration, a reproductive toxicity test in rats during the teratogenic sensitive period, and a reproductive toxicity test in rabbits during the teratogenic sensitive period.

Preclinical Pharmacodynamic Study

Study on the in vitro Pharmacodynamic Effects of ZG0163 at the Biochemical Level

ZG0163 significantly inhibited the phosphorylation of STAT3 by JAK1, JAK2, and the IC50 of ZG0163 for JAK1, JAK2 was about 0.1 μM. In this study, the effects of ZG0163 was similar to those of positive control compounds, ZG0164 and ZG0178.

ZG0163 also significantly inhibited the phosphorylation of Elk-1 by ERK in the JAK signaling pathway. However, ZG0163 showed no significant effects on the phosphorylation of IκBα by IKKβ and c-Jun by JNK, indicating that ZG0163 was a selective JAK inhibitor.

Study on the in vitro Pharmacodynamic Effects of ZG0163 at the Cellular Level (Ba/F3-EpoR-JAK2V617F cells)

The results of in vitro pharmacodynamic studies showed that in Ba/F3-EpoR-JAK2V617F cell line, the compound ZG0163 was able to induce apoptosis of pathogenic cells and eliminate abnormal cell proliferation by inhibiting the JAK-STAT signaling pathway, thus achieving treatment efficacy in myeloproliferative neoplasms. ZG0163 could significantly inhibit JAK-STAT signaling in Ba/F3-EpoR- JAK2V617F cell line, manifested as significant inhibition of phosphorylation of JAK2 and its downstream signaling proteins ERK and STAT5. The effects of ZG0163 was similar to those of positive control compounds, ZG0164 and ZG0178.

ZG0163 showed significant inhibitory effect on the cell activity of mutant Ba/F3-EpoR-JAK2V617F, which was similar to those of positive control compounds, ZG0164

and ZG0178. However, the inhibitory effect on the activity of Ba/F3-EpoR cell line was relatively weak. The IC50 values of the inhibitory effects were 0.5 μM and 1.5 μM, respectively. Obviously, this selective inhibition is associated with the aberrant expression of JAK2 in Ba/F3-EpoR-JAK2V617F mutant cells, and the degree of ZG0163 inhibition on JAK-STAT signaling pathway in this cell line.

ZG0163 could significantly induce the apoptosis of Ba/F3-EpoR-JAK2V617F cells, and the effects of ZG0163 was similar to those of positive control compounds, ZG0164 and ZG0178.

In Vitro Inhibitory Effect of ZG0128 on Cell Lines Transfected with Oncogenes and Primary Blood Cells in Patients with Polycythemia Vera

In vitro cell culture assay was used to determine whether compound ZG0128 significantly inhibited the growth of cells transfected with oncogenes, including J53Z1 cell line harboring a JAK2V617F mutation, and 8 HCD-57 cell lines stably transfected with oncogenic mutations JAK2V617F, MPLW515L, KITD816V, NPM-ALK, FLT3-ITD, FLT3D835H, FLT3D835Y and KRasG12V, respectively. In addition, study the inhibitory effects of compound ZG0128 on the growth of hematopoietic progenitor cells in patients with polycythemia vera.

The results showed that the inhibitory effects of compound ZG0128 on the above 9 cell lines were similar to those of positive control drug ZG0192. At the sub-micromolar concentration, both compounds ZG0128 and ZG0192 were able to effectively inhibit the growth of J53Z1 cells harboring the JAK2V617F mutation and the growth of HCD-57 cells transfected with oncogenic mutations JAK2V617F, MPLW515L, KITD816V, FLT3-ITD, FLT3D835H and FLT3D835Y, respectively.
The IC50 of these two compounds was 0.31-0.71 μM. ZG0128 showed a broad-spectrum inhibitory effect on tyrosine kinases.

95% of patients with polycythemia vera carried JAK2V617F mutations, while the analysis result of peripheral blood samples of all patients showed positive JAKV617F. The results obtained from the 9 patients with polycythemia vera showed that compound

ZG0128 almost completely inhibited the colony forming of erythroid hematopoietic cells at a concentration of 1 μM. ZG0128 not only reduced the number of hematopoietic colony, but also reduced the size of these colonies. The IC50 of inhibition was 0.18 μM, which was lower than the IC50 value (0.61 μM) of inhibitory effect on transfected JAK2V617F cell lines. These data demonstrated at the ex-vivo level that ZG0128 could effectively affect the primary cells harboring the JAK2V617F mutation in patients with polycythemia vera.

In Vivo Pharmacodynamic Study of ZG0128 in Mouse Model of Myeloproliferative Neoplasm

Primary myelofibrosis (PMF) is characterized by the formation of bone marrow fibrous tissues (scar-like) caused by the abnormal proliferation of three types of cells (platelets, white blood cells, and red blood cells). At present, there is still no particularly effective treatment for these diseases. JAKV617F transgenic mice and JAK2V617F bone marrow transplanted mice can develop the pathological characteristics of myeloproliferative neoplasm, which can be used to effectively evaluate the efficacy of drugs used in the treatment of myeloproliferative neoplasms.

HCD-57-JAK2V617F cells were intravenously injected into NSG mice. Three weeks later, the mice were injected intraperitoneally with a single dose of 200 mg/kg of compound ZG0128, and euthanized 4 h after drug administration. The results of histochemical staining and flow cytometry analysis showed that more than 90% of the nucleated cells in spleen were HCD-57-JAK2V617F transplanted cells. Western blot analysis of splenocyte protein revealed that compound ZG0128 significantly inhibited the phosphorylation of downstream major signaling proteins of JAK2, i.e., ERK1/2, Akt and STAT5. These results indicated that the compound ZG0128 could effectively inhibit the JAK2 signaling pathway in vivo.

9 weeks old JAK2V617F transgenic mice were given a daily dose of 25 mg/kg or 100 mg/kg ZG0128 by oral gavage. After 4 weeks of treatment, the high-dose group showed a significant decrease in the counts of three types of blood cells (red blood cells,

platelets, and white blood cells), and the spleen volume was also significantly reduced compared with that before the treatment. The low-dose group showed a similar trend, i.e., the counts of three types of blood cells (red blood cells, platelets, and white blood cells) and the spleen volume were both reduced compared with those before the treatment, with no significant statistical differences. The above results showed that in JAK2V617F transgenic mouse model, ZG0128 significantly inhibited the pathological characteristics of myeloproliferative neoplasms (MPNs) by reducing the blood cell counts and the spleen volume.

The ability of compound ZG0128 and its positive control drug ZG0192 to inhibit the pathological characteristics of myeloproliferative tumors (MPNs) was evaluated in a JAK2V617F bone marrow transplanted mouse model. JAK2V617F bone marrow transplanted mice were given a daily dose of 25 mg/kg or 100 mg/kg ZG0128 or positive control drug ZG0192 for four weeks by oral gavage. The results showed that, compared to the control group given a blank vehicle, the intragastric administration of 100 mg/kg of compound ZG0128 could significantly reduce blood cell counts and spleen volume. The low-dose group showed a similar trend, i.e., the counts of three types of blood cells (red blood cells, platelets, and white blood cells) and the spleen volume were both reduced compared with those before the treatment. Some data showed no significant statistical differences. Compared to the blank vehicle control group, ZG0128 was able to significantly slow the further increase in the percentage of CD45.2+ JAK2V617F cells during treatment. ZG0128 and the positive control compound ZG0192 showed similar therapeutic effects and strength.

Myelofibrosis is a characteristic of disease progression in patients with intermediate or high-risk myeloproliferative neoplasms (MPNs), with the worst prognosis. Therefore, we transplanted the bone marrow of JAK2V617F transgenic mice into mice treated with lethal radiation exposure. 8 weeks later, after developing obvious pathological characteristics of myelofibrosis, the mice were orally/intragastrically administered 100 mg/kg of ZG0128 or positive control drug ZG0192 for 5 weeks. The

results showed that ZG0128 not only significantly reduced blood cell counts and spleen volume, but also significantly and effectively inhibited the progression of myelofibrosis, as shown in reticular fiber staining of bone tissue and quantitative detection of hydroxyproline in bone marrow. ZG0128 and the positive control compound ZG0192 showed similar blocking effect on the progression of myelofibrosis.

Preclinical Toxicology Test Results

According to the relevant requirements for the non-clinical toxicology study of national Class 1 new drug as well as the GLP standard, the completed toxicological tests include acute toxicity tests: a toxicity test in Wistar rats administered a single-dose of ZG0128 by oral gavage, and a toxicity test in Beagle dogs orally administered a single-dose of ZG0128; long-term toxicity tests: a toxicity test in Wistar rats administered ZG0128 by oral gavage for 28 days, followed by 28 days of recovery, and a toxicity test in Beagle dogs orally administered ZG0128 for 28 days, followed by 28 days of recovery; genotoxicity tests: including Ames tests, chromosome aberration tests and mouse bone marrow micronucleus tests; tests for reproductive and developmental toxicity: a toxicity test on fertility and early embryonic development in Wistar rats administered ZG0128 by oral gavage, and a toxicity test in Wistar rats administered ZG0128 by oral gavage for 26 weeks, followed by 4 weeks of recovery.

Acute Toxicity Test

A Toxicity Test in Wistar Rats Administered a Single-Dose of ZG0128 By Oral Gavage

The study consisted of 4 groups, including the control group and dose groups of 244, 732, and 2440 mg/kg of ZG0128 (corresponding to 200, 600, and 2000 mg/kg of the active ingredient ZG0163). Each group contained 10 rats, with 5 male rats and 5 female rats. The rats in each group were given a single-dose of control substance (0.5% CMC-Na solution) or corresponding concentrations of ZG0128 in a 20 mL/kg volume by oral gavage. The rats were observed for 14 days after drug administration. The day

of dosing is defined as day 1 of the trial. During the trial, the general conditions of the rats in different groups were observed every day; the body weight of the rats was measured once on days 1, 3, 8, and 14, respectively, and the 24-h food intake was measured once on days 3, 7, and 13, respectively. After 14 days of observation, the surviving rats in each group were anesthetized to collect blood samples for hematology and blood biochemistry tests. All rats were euthanized for gross anatomy.

Results showed that Wistar rats were given a single-dose of 244, 732, and 2440 mg/kg of ZG0128 by oral gavage, and the maximum tolerated dose (MTD) was 732 mg/kg.

A Toxicity Test in Beagle Dogs Orally Administered a Single-Dose of ZG0128

The study consisted of 3 groups, including the control group and dose groups of 366 and 1220 mg/kg of ZG0128 (corresponding to 300 and 1000 mg/kg of active ingredient ZG0163). Each group contained 4 Beagle dogs, with 2 male dogs and 2 female dogs. The dogs in each group were orally administered a single-dose of control (empty capsule) or the corresponding doses of ZG0128, and were observed for 14 days after drug administration. The day of dosing is defined as day 1 of the trial. During the trial, the general conditions of the dogs in different groups were observed every day; the body weight of the dogs was measured on days 1, 4, 8, and 14, respectively, and the 24-h food intake was measured on days 3-4, 7-8, and 13-14, respectively. About 2-3 and 24-25 h after drug administration as well as on day 15 of the trial, lead II electrocardiogram and blood pressure were determined; the hematology and blood biochemistry tests were performed on days 2 and 15. After 14 days of observation, all dogs were anesthetized and euthanized for gross anatomy. Blood samples were collected from rats before drug administration and 0.5, 1, 2, 4, 8 and 24 h after drug administration in ZG0128 dose groups. LC-MS/MS was used to measure the concentration of active ingredient ZG0163 at various time points and to calculate AUClast, Cmax, Tmax, and other toxicokinetic parameters.

Results showed that after the Beagle dogs were orally administered a single-dose of 366 and 1220 mg/kg of ZG0128 (corresponding to 300 and 1000 mg/kg of active ingredient ZG0163), the AUC0-t was 10215 ± 10044 ng/mL and 19752 ± 9998 ng/mL, respectively, and the maximum tolerated dose (MTD) was 1220 mg/kg.

Long-Term Toxicity Test

A Toxicity Test in Wistar Rats Administered ZG0128 by Oral Gavage for 28 Days

The study consisted of 4 groups, including the control group and dose groups of 24.4, 73.2, and 219.6 mg/kg of ZG0128 (corresponding to 20, 60, and 180 mg/kg of active ingredient ZG0163). The control group contained 36 Wistar rats (6 rats were used for the toxicokinetic test) and each group of ZG0128 contained 54 rats (24 rats in each group were used for the toxicokinetic test), with half males and half females. The rats in each group were given the control substance (0.5% CMC-Na solution) or the corresponding concentrations of ZG0128 in a 10 mL/kg volume by oral gavage once a day for 28 consecutive days, followed by 28 days for recovery. The day of the first dose is defined as day 1 of the dosing period, while the day of anatomy after the last dose is defined as day 1 of the recovery period. During the dosing period and the recovery period, the general conditions of the rats in different groups were observed every day; the body weight and food intake of the rats were measured twice a week during the dosing period and once a week during the recovery period, respectively. One ophthalmic examination and routine urinalysis were arranged at the end of dosing and recovery periods, respectively. In the 219.6 mg/kg group, 12 (3 females, 9 males) and 2 (2 males) rats were dissected at the end of dosing and recovery periods, respectively. In all other groups, 20 and 10 rats (male:female=1:1) were dissected at the end of dosing and recovery periods, respectively. The rats in each group were anesthetized to collect blood samples for hematology and blood biochemistry tests. After the rats were euthanized, bone marrow smear, gross anatomy, organ weight measurement and histopathological examination of tissue samples were performed. For the rats in

ZG0128 groups used for toxicokinetic test, their blood samples were collected before the first dose and last dose as well as 0.5, 1, 2, 3, 4, 6, 10, and 24 h after the first dose and last dose. For the rats in the control group, their blood samples were collected before the first dose and last dose and 3 h after the first dose and last dose, respectively. LC-MS/MS was used to measure the concentrations of the original drug ZG0163 and its amide hydrolysis metabolite ZG0245 in the blood samples collected at various time points, and to calculate AUClast, Cmax, Tmax, and other toxicokinetic parameters.

The results showed that the dose of 219.6 mg/kg could cause dying or death in rats. The main toxic target organs were immune and hematopoietic systems (lymphoid tissues, spleen, thymus, and bone marrow), digestive system (gastrointestinal tract, pancreas, and submandibular gland), reproduction system, kidney, and bones. The drug caused abnormal indicators related to liver, renal, and pancreatic functions, as well as decreased lymphocytes and platelets. At the dose of 73.2 mg/kg, some of the above lesions could be observed, but the severity of the lesions was mild. After drug discontinuation, the lesions in the submandibular gland, testis, and epididymis were still visible in 73.2 and 219.6 mg/kg groups, and the severity of the lesions was not alleviated. The dose of 24.4 mg/kg only caused ovarian lesions, which were disappeared after drug discontinuation.

In the dose range of 24.4 to 219.6 mg/kg, the exposure to ZG0128 and its metabolite ZG0245 in female and male rats increased in proportion to the dose, and no significant gender difference was observed. After 28 days of consecutive administration, no significant accumulation of ZG0128 and its metabolite ZG0245 was observed in both female and male rats of each dose group.

After the Wistar rats were given 24.4, 73.2, and 219.6 mg/kg of ZG0128 by oral gavage for 28 consecutive days, the highest non-severely toxic dose (HNSTD) was 24.4 mg/kg. At this dose, the drug exposure dose in female rats on the first day of dosing was 60887 ± 21040 h•ng/mL (based on the active ingredient ZG0163), while 50799 ± 10124 h•ng/mL in male rats. On day 28 of dosing, the drug exposure dose in female

rats was 51893 ± 20625 h•ng/mL (based on the active ingredient ZG0163) while 46522 ± 17149 h•ng/mL in male rats.

A Toxicity Test in Beagle Dogs Administered ZG0128 by Oral Gavage for 28 Days

The study consisted of 4 groups, including the control group and dose groups of 18.3, 61, and 195.2 mg/kg of ZG0128 (corresponding to 15, 50, and 160 mg/kg of the active ingredient ZG0163). Each group contained 10 dogs, with 5 male dogs and 5 female dogs. The dogs in each group were orally administered with control (empty capsule) or the corresponding doses of ZG0128 once a day for 28 consecutive days, followed by 28 days for recovery after drug discontinuation. The day of the first dose is defined as day 1 of the dosing period. During the dosing period and the recovery period, the general conditions of the dogs in different groups were observed every day. The body weight and food intake of the dogs were measured twice a week during the dosing period and once a week during the recovery period, respectively. At 2-3 h after the dosing on day 14, and 2-3 h after the last dose as well as on the last day of the recovery period. The lead II electrocardiogram, blood pressure and body temperature (anal temperature) were measured. One ophthalmic examination and routine urinalysis were arranged at the end of dosing and recovery periods, respectively. Hematology and blood biochemistry tests were performed on day 15 and the end of the dosing and recovery periods, respectively. In the 195.2 mg/kg group, 3 dogs (2 females, 1 male) were dissected at the end of recovery period. In all other groups, 6 and 4 dogs (male:female=1:1) were dissected at the end of dosing and recovery periods, respectively. After the dogs were euthanized, bone marrow smear, gross anatomy, organ weight measurement and histopathological examination of tissue samples were performed. Blood samples were collected from dogs before the first/last dose and 0.5, 1, 2, 4, 6, 10, 24 h after drug administration in ZG0128 dose groups. Blood samples were collected before the first/last dose and 2 h after the first/last dose, respectively. LC-MS/MS was used to measure the concentration of the original drug ZG0163 and

the amide hydrolysis metabolite ZG0245 in blood samples at various time points and to calculate AUClast, Cmax, Tmax, and other toxicokinetic parameters.

The results showed that, after the Beagle dogs were orally administered with 18.3, 61, and 195.2 mg/kg of ZG0128 for 28 consecutive days and recovered for 28 days after drug discontinuation, the dose of 195.2 mg/kg could cause death in dogs. The main toxic target organs were immune and hematopoietic systems (lymphoid tissues, spleen, thymus, and bone marrow), liver, and male reproductive organs. In the 61 mg/kg group, only milder and less frequent lesions were observed in lymphoid tissues and liver, and such lesions could be alleviated to a certain extent after drug discontinuation. The 18.3 mg/kg group only showed a reduced number of thymic lymphocytes and this abnormality disappeared after drug discontinuation. No other abnormal changes were observed in various ZG0128 groups in terms of lead II ECG, body temperature, ophthalmic examination, blood biochemistry, and urinalysis parameters.

In the dose range of 18.3 to 195.2 mg/kg, the exposure to ZG0128 and its metabolite ZG0245 in Beagle dogs increased in proportion to the dose and no significant gender difference was observed, although plasma exposure dose showed large individual differences. After 28 days of consecutive administration, ZG0128 and its metabolite ZG0245 accumulated to a degree in both female and male dogs of each dose group of ZG0128.

After the Beagle dogs were orally administered with 18.3, 61, and 195.2 mg/kg of ZG0128 for 28 consecutive days, the highest non-severely toxic dose (HNSTD) was 61 mg/kg. At this dose, the drug exposure dose in female dogs on day 28 of the dosing period was 11315 ± 8890 h•ng/mL (based on the active ingredient ZG0163) while 5069 ± 3653 h•ng/mL in male dogs. The no observed adverse effect level (NOAEL) was 18.3 mg/kg. At this dose, the drug exposure dose in female dogs on day 28 of dosing was 2372 ± 1343 h•ng/mL (based on the active ingredient ZG0163) while 1301 ± 699 h•ng/mL in male dogs.

A Toxicity Test in Wistar Rats Administered ZG0128 by Oral Gavage for 26 Consecutive Weeks

Wistar rats were given 4, 12, or 30 mg/kg of ZG0128 via oral gavage once a day for 26 consecutive weeks, followed by 28 days for recovery after drug discontinuation. Only the 30 mg/kg dose caused a slight decrease in lymphocyte counts, which might be related to the pharmacological effects of ZG0128. The lymphocyte counts were back to normal by the end of recovery period. No other obvious abnormalities related to the investigational drug were observed in various groups of rats in terms of their general conditions, body weight, ophthalmologic examination, blood biochemistry, blood coagulation, routine urinalysis, gross anatomy and histopathological examination during the trial. The no observed adverse effect level (NOAEL) was 30 mg/kg. At this dose, the drug exposure was 52697 h•ng/mL (based on the active ingredient ZG0163) in female rats and 67133 h•ng/mL in male rats on the first day of dosing, and on day 182 of dosing the drug exposure was 75339 h•ng/mL (based on the active ingredient ZG0163) in female rats and 45024 h•ng/mL in male rats.

Genotoxicity Test

Ames Test

The results of the Ames test showed that, in the presence or absence of an S9 metabolic activation system, 9.76, 48.8, 244, 1220, and 6100 μg/mL of ZG0128 did not cause genetic mutations in any of the test strains. i.e. the results of the Ames test were negative.

In Vitro Chromosome Aberration Test in CHL Cells

An in vitro chromosomal aberration test of ZG0128 was performed in CHL cells. The results showed that after 4 h of exposure in the presence or absence of an S9 metabolic activation system, 3.05, 6.1, 12.2, and 24.4 μg/mL of ZG0128 did not significantly increased the rate of chromosomal structural aberrations in CHL cells. After 24 h of exposure in the absence of an S9 metabolic activation system, 0.7625, 1.525, 3.05 and 6.1 μg/mL of ZG0128 did not significantly increased the rate of

chromosomal structural aberrations in CHL cells. Therefore, the results of the chromosomal aberration test were negative.

Mouse Bone Marrow Micronucleus Test

A mouse bone marrow micronucleus test of ZG0128 has been performed. After multiple doses of ZG0128 are given to mice via oral gavage, the incidence of micronucleated polychromatic erythrocytes in the bone marrow is observed to estimate the damage to chromosomal integrity in bone marrow cells and subsequent abnormality of chromosome segregation, so as to determine the presence of genotoxicity. The results showed: After NIH mice were given 122, 244, or 488 mg/kg of ZG0128 by oral gavage once a day for 4 consecutive days, neither damage to the chromosomal integrity in bone marrow cells nor abnormal chromosome segregation was observed. Therefore, the results of the micronucleus test were negative.

A Toxicity Test on Fertility and Early Embryo Development in Wistar Rats Administered ZG0128 by Oral Gavage

A toxicity test on the fertility and early embryo development in Wistar rats given ZG0128 by oral gavage was completed. In this test, female and male rats were given a continuous dose of ZG0128 prior to mating until embryo implantation to observe the toxicity or interference of ZG0128 on the fertility and early embryo development in Wistar rats. The study consisted of 4 groups, including the control group and dose groups of 18, 36, and 72 mg/kg of ZG0128. 192 Wistar rats were randomly divided into these 4 groups, 48 rats in each (male:female=1:1). The rats in each group were given corresponding concentrations of ZG0128 or 0.5% CMC-Na solution at 10 mL/kg by oral gavage. The male rats in different groups were dissected in batches in week 10 after dosing. The rats were mated in week 4 after dosing. The female rats were given a continuous dose of ZG0128 once a day from 2 weeks before mating to day 7 of gestation. At the time of mating, male and female rats were placed in cages in a 1:1 ratio. The day when sperms were found in vaginal plugs or vaginal smears was defined as day 0 of gestation. During the trial, the general conditions of the rats in each group

were observed every day. The body weight of male rats was weighed twice a week. The body weight of female rats was measured twice a week before pregnancy and on days 0, 2, 4, 6, 9, 12, and 15 of gestation after successful mating. The food intake of male and female rats was measured once a week before mating. After successful mating, the food intake of female rats was measured on days 0, 6, and 12 of gestation. The estrous cycle in female rats was examined daily from the first dose to the end of mating. After the dosing, male rats were euthanized to collect the left epididymis for the tests of sperm motility, sperm count and sperm abnormality. In addition, the weights of testes, epididymides, prostate, and seminal vesicles were measured to calculate the organ coefficients. The female rats that have successfully mated were euthanized on day 15 of pregnancy and dissected to record the following numbers: corpus luteum, deciduoma, stillbirths, live fetuses, and absorbed fetuses. In addition, the weights of the pregnant uterus, placenta and uterus, placenta, ovaries, brain, and the total weight of live fetuses were measured. The weight of the uterus (the weight of the uterus = the weight of the placenta and uterus – the weight of the placenta) and the organ coefficient of ovaries were calculated. The mating rate, the rate of pre-implantation loss, the rate of post-implantation loss, and the rate of total loss were calculated.

The results showed that a certain level of parental, reproductive and embryo toxicity was noticed in the 36 and 72 mg/kg groups. Parental toxicity was mainly manifested as decreased body weight and reduced food intake. Reproductive toxicity was mainly manifested as decreased sperm counts, an increased rate of sperm abnormality, abnormal changes in indicators related to sperm motility, and decreased organ weight/coefficient of the testis and epididymis in male rats. In pregnant rats, reproductive toxicity was mainly manifested as reduction in the corpus luteum count, the number of deciduoma, the number of live fetuses and pregnancy rate, increase in the number of absorbed fetuses, the rate of post-implantation loss and the rate of total loss, and reduced organ weight/coefficient of the uterus and ovaries. Embryo toxicity was mainly manifested as decreased average/total weight of pregnant uterus, placenta

+ uterus, placenta, and live fetuses. Histopathological examination also showed decreased sperm counts as well as the presence of cell debris in the duct of the epididymis in male rats. In pregnant rats, histopathological examination showed the atrophy of uterus glands, hyperplasia/inflammation in the decidua of uterus wall, and the atrophy of corpus luteum. The severity and incidence of the aforementioned parental, reproductive and embryo toxicities were lower in the 36 mg/kg group than in the 72 mg/kg group. No interference or toxic effects of ZG0128 on reproductive functions and embryonic development in parental and male/female rats were observed in the 18 mg/kg group. The no observed adverse effect level (NOAEL) of ZG0128 for reproductive functions and embryonic development in parental and male/female rats was 18 mg/kg.

Preclinical Pharmacokinetic Properties

Pharmacokinetic Study of Single Dose Intravenous Administration in Rats

ZG0163 administered via intravenous injection had low plasma clearance in rats, with a CL of 4.07 mL/min/kg and Vss of 0.514 L/kg. The Tmax of the metabolite ZG0245 in rats was 1.0 h on average, and the plasma exposure (AUC0-t) was 15.0% of the original drug. The pharmacokinetic parameters obtained after the intravenous injection of the drug into rats are shown in Tables 10 and 11.

Table 10. Pharmacokinetic parameters of ZG0163 in rats administered with 6.1 mg/kg of ZG0128 via intravenous injection.

Gender	AUC_{0-t} (μg·h/mL)	$AUC_{0-\infty}$ (μg·h/mL)	MRT (h)	$t_{1/2}$ (h)	CL (mL/min/kg)	V_{ss} (L/kg)
Male	17.7 ± 4.4	17.7 ± 4.4	2.16 ± 0.48	2.01 ± 0.67	4.92 ± 1.34	0.620 ± 0.120
Female	26.0 ± 2.9	26.0 ± 2.9	2.11 ± 0.37	2.01 ± 0.57	3.23 ± 0.38	0.407 ± 0.063
Overall	21.8 ± 5.6	21.9 ± 5.6	2.14 ± 0.38	2.01 ± 0.55	4.07 ± 1.28	0.514 ± 0.145

Table 11. Pharmacokinetic parameters of metabolite ZG0245 in rats administered with 6.1 mg/kg of ZG0128 via intravenous injection.

Gender	T_{max} (h)	C_{max} (μg/mL)	AUC_{0-t} (μg·h/mL)	$AUC_{0-\infty}$ (μg·h/mL)	MRT (h)	$t_{1/2}$ (h)
Male	1.0 (1.0-2.0)	0.578 ± 0.074	2.51 ± 0.77	2.60 ± 0.75	4.71 ± 1.79	4.93 ± 2.78
Female	1.0 (1.0)	0.837 ± 0.102	4.05 ± 0.54	4.18 ± 0.65	6.78 ± 2.41	7.17 ± 1.06
Overall	1.0 (1.0-2.0)	0.707 ± 0.162	3.28 ± 1.03	3.39 ± 1.07	5.75 ± 2.21	6.05 ± 2.24

Pharmacokinetic Study of Single-Dose Intragastric Administration in Rats

After a single intragastric administration of 15.3, 30.5 and 61 mg/kg of ZG0128 in rats, the T_{max} of ZG0163 in rats was 0.75-3.0 h, and the peak concentrations of the drug in three dose groups were 5.74, 8.33 and 12.4 μg/mL, respectively. The systemic exposure (AUC_{0-t}) was 21.9, 40.8 and 103 μg•h/mL, respectively, and the plasma elimination half-life $t_{1/2}$ was 0.89-1.85 h. The systemic exposure (AUC0-t) in male rats was 1.04-1.50 times that of female rats, but no significant gender difference was observed. The Tmax of the metabolite ZG0245 in rats was 2.0-5.0 h, which was slightly longer than that of the original drug. The exposure (AUC0-t) of ZG0245 was 14.9% of that of the original drug. This value was consistent with the ratio obtained after intravenous drug administration, indicating that the metabolite ZG0245 was not generated by first-pass metabolism after intragastric administration of the drug. In the dose range of 15.3-61 mg/kg, the increase in the Cmax of ZG0163 and the metabolite ZG0245 was lower than the proportional increase in dose, the increase in the AUC0-t of ZG0163 was directly proportional to the increase in dose, and the increase in the AUC0-t of the metabolite ZG0245 was slightly higher than the proportional increase in dose. Based on the average value of AUC0-t, the absolute bioavailability of the drug in rats receiving a single intragastric administration of 15.3 mg/kg of ZG0163 was 40.0%.

Pharmacokinetic Study of Multiple-Dose Intragastric Administration in Rats

After 7 consecutive days of continuous intragastric administration (30.5 mg/kg once a day) of ZG0128 in rats, the plasma concentration reached a steady state on day 5. The Cmax and AUC0-t of ZG0163 obtained on day 7 were 1.04 and 1.27 times the values obtained after the single-dose administration, respectively. The Cmax and AUC0-t of the metabolite ZG0245 were 1.19 and 1.52 times the values obtained after the single-dose administration, respectively. These results indicated that after 7 consecutive days of administration, there was no significant accumulation of ZG0163 and the metabolite ZG0245 in rats. The pharmacokinetic parameters obtained after single and multiple intragastric administration of the drug in rats are shown in Tables 12 and 13.

Table 12. Pharmacokinetic parameters of ZG0163 in rats given different doses of ZG0128 via oral gavage (n = 6, male:female=1:1).

ZG0128 Dosage (mg/kg)	Gender	T_{max} (h)	C_{max} (μg/mL)	AUC_{0-t} (μg·h/mL)	$AUC_{0-\infty}$ (μg·h/mL)	MRT (h)	$t_{1/2}$ (h)
15.3	Male	1.0 (0.5-2.0)	6.66 ± 2.13	24.5 ± 11.3	24.6 ± 11.3	2.45 ± 0.29	0.84 ± 0.20
	Female	0.5 (0.5-1.0)	4.82 ± 1.25	19.4 ± 7.5	19.4 ± 7.5	2.82 ± 0.60	0.93 ± 0.26
	Overall	0.75 (0.5-2.0)	5.74 ± 1.86	21.9 ± 9.0	22.0 ± 9.0	2.63 ± 0.47	0.89 ± 0.21
30.5	Male	1.0 (1.0-2.0)	6.97 ± 1.15	41.6 ± 19.7	43.2 ± 21.9	3.95 ± 1.68	1.58 ± 0.75
	Female	2.0 (2.0)	9.68 ± 3.40	39.9 ± 13.6	40.2 ± 13.2	3.46 ± 0.75	2.07 ± 2.24
	Overall	2.0 (1.0-2.0)	8.33 ± 2.71	40.8 ± 15.2	41.7 ± 16.2	3.70 ± 1.19	1.82 ± 1.52
61	Male	2.0 (2.0-8.0)	12.8 ± 1.7	124 ± 58	124 ± 58	6.20 ± 2.52	1.56 ± 0.59
	Female	4.0 (1.0-6.0)	12.1 ± 2.6	82.7 ± 31.0	87.3 ± 31.0	4.87 ± 1.22	2.14 ± 0.31
	Overall	3.0 (1.0-8.0)	12.4 ± 2.0	103 ± 47	106 ± 46	5.54 ± 1.92	1.85 ± 0.53
30.5 (multiple dose)	Male	1.0 (1.0-2.0)	6.34 ± 1.23	35.7 ± 13.5	36.0 ± 13.8	/	2.15 ± 0.62
	Female	1.0 (1.0-6.0)	11.0 ± 4.22	67.6 ± 27.6	67.8 ± 27.8	/	1.81 ± 1.55
	Overall	1.0 (1.0-6.0)	8.67 ± 3.77	51.7 ± 26.1	51.9 ± 26.2	/	1.98 ± 1.07

Table 13. PK parameters of metabolite ZG0245 in rats after intragastric administration of different dosages of ZG0128 (n = 6, half males and half females).

ZG0128 Dosage (mg/kg)	Gender	T_{max} (h)	C_{max} (μg/mL)	AUC_{0-t} (μg·h/mL)	$AUC_{0-\infty}$ (μg·h/mL)	MRT (h)	$t_{1/2}$ (h)

15.3	Male	2.0 (1.0-2.0)	0.635 ± 0.260	2.98 ± 1.49	3.02 ± 1.50	3.88 ± 0.50	2.46 ± 1.33
	Female	2.0 (1.0-2.0)	0.551 ± 0.145	2.95 ± 0.82	3.04 ± 0.86	4.10 ± 0.35	2.01 ± 0.12
	Overall	2.0 (1.0-2.0)	0.593 ± 0.194	2.96 ± 1.07	3.03 ± 1.09	3.99 ± 0.40	2.24 ± 0.88
30.5	Male	2.0 (2.0-8.0)	0.765 ± 0.105	5.76 ± 3.51	5.82 ± 3.48	5.28 ± 2.11	2.31 ± 0.89
	Female	2.0 (2.0)	1.04 ± 0.33	6.32 ± 1.50	6.39 ± 1.44	6.38 ± 2.08	4.72 ± 1.92
	Overall	2.0 (2.0-8.0)	0.902 ± 0.265	6.04 ± 2.43	6.11 ± 2.40	5.83 ± 1.96	3.51 ± 1.88
61	Male	8.0 (4.0-12.0)	1.49 ± 0.33	16.6 ± 8.2	16.7 ± 8.2	7.73 ± 2.48	3.72 ± 0.79
	Female	2.0 (2.0-6.0)	1.73 ± 0.75	17.4 ± 8.9	17.4 ± 8.9	6.33 ± 1.62	2.54 ± 1.06
	Overall	5.0 (2.0-12.0)	1.61 ± 0.53	17.0 ± 7.7	17.1 ± 7.7	7.03 ± 2.03	3.13 ± 1.06
30.5 (multiple dose)	Male	2.0 (2.0)	0.673 ± 0.130	4.97 ± 2.21	5.06 ± 2.14	/	4.30 ± 1.68
	Female	4.0 (2.0-8.0)	1.48 ± 0.61	13.4 ± 5.5	13.6 ± 5.6	/	5.47 ± 2.27
	Overall	2.0 (2.0-8.0)	1.07 ± 0.59	9.18 ± 5.9 3	9.35 ± 6.04	/	4.88 ± 1.90

Pharmacokinetics of single dose intravenous administration in beagles.

After intravenous administration of 12.2 mg ZG0128 (the average weight of the beagles was about 10 kg, so the dose given equals to 1.22 mg/kg ZG0128), ZG0163 had a high plasma clearance CL of 49.3 mL/min/kg and volume of distribution Vss of 3.49 L/kg. The average Tmax of metabolite ZG0245 was 1.0 h, and the plasma exposure AUC0-t was 70.1% of that of the original drug.

Pharmacokinetics of single-dose intragastric administration.

The average Tmax of ZG0163 was 2.0 h after a single-dose intragastric administration of 1, 2, and 4 ZG0128 tablets (corresponding to 61, 122, and 244 mg/ZG0128). The Cmax was 156, 266, and 444 ng/mL, respectively. The plasma exposure AUC0-t was 459, 948, and 1728 ng•h/mL (mean) and the average plasma elimination half-life t1/2 was 1.31-2.49 h. There were large individual differences in plasma exposure AUC0-t in dogs. The pharmacokinetic differences between genders could not be determined. Within the range of 61-244 mg, the AUC0-t of ZG0163 increased proportionally with the increasing dosage. The average Tmax of metabolite ZG0245 was 2.0 h. The plasma exposure of metabolite ZG0245 was roughly 2.16 times

higher than ZG0163, which was higher than the ratio of intravenous administration. This suggested that after intragastric administration, part of the metabolite ZG0245 was produced through first-pass metabolism. Within the dosage range of 61-244 mg, the AUC0-t of ZG0245 increased proportionally with the increasing dosage.

Pharmacokinetics of 7 consecutive days of multi-dose intragastric administration.

After 7 consecutive days of intragastric administration (2 tablets once daily, equal to 122 mg/animal), plasma concentrations reached steady state on day 5. On day 7, the Cmax and AUC0-t of ZG0163 in male beagles were 1.26 and 1.07 times higher, respectively, when compared to single-dose administration. The Cmax and AUC0-t of metabolite ZG0245 were 1.33 and 0.95 times compared to single-dose administration. Thus, there was no significant accumulation of ZG0163 and metabolite ZG0245 after 7 days of consecutive administration.

Calculated with AUC0-t, the absolute bioavailability in beagles was 27.2% after intragastric administration of 61 mg ZG0163.

The Tmax of ZG0163 was 1.50 h after intragastric administration of 122 mg ZG0128, and the exposure was 93.0% of that of the medium dosage group (122 mg/animal). The Tmax of metabolite ZG0245 was 2.0 h, and the exposure was 91.7% of that of the medium dosage group (122 mg/animal).

Identification of Metabolites in Rat Samples

Using UPLC-UV/Q-TOF MS, potential metabolites were identified in rat plasma, urine, feces and bile, after single intragastric administration of 30.5 mg/kg ZG0128, as well as metabolites in mice plasma after single intragastric administration of 61 mg/kg ZG0128. Judging from the UV chromatogram peaks areas, the metabolites in rat plasma were mainly the original drug, followed by amide hydrolysis metabolite M2. Due to matrix interference, ZG0128 metabolites in feces of rats cannot be clearly distinguished in UV chromatograms. Judging from the relative LC-MS peaks areas, the metabolites

detected in the urine samples of male rats were mainly the original drug, followed by amide hydrolysis metabolite M2 and morpholine ring-opening metabolites (M3 and M4); metabolites detected in the urine samples of female rates were mainly M2, followed by M4, M3, and M0. Metabolites detected in feces samples of rats were mainly the original drug M0 and amide hydrolysis metabolite M2, followed by morpholine ring-opening metabolites (M3 and M4) and morpholine ring double oxidative dehydrogenation metabolite M8. Metabolites detected in bile samples of male rats were mainly morpholine ring-opening metabolites (M3 and M4), morpholine ring double oxidative dehydrogenation metabolite M8, glutathione conjugate metabolite M13-1, and amide hydrolysis metabolite M2; metabolited detected in bile samples of female rats were mainly M2, followed by M13-1, M8, M3 and M4.

After intragastric administration of ZG0128, metabolites detected in mice plasma were primarily the original drug M0 and amide hydrolysis metabolite M2.

The metabolic stability of ZG0128 (the dihydrochloride monohydrate of ZG0163) was evaluated using human, monkey, dog, rat, and mouse liver microsomes as the in vitro models. The percentages of residues for ZG0128 after incubating in human, monkey, dog, rat, and mouse liver microsomes for 60 minutes was 55.5%, 63.1%, 67.1%, 80.3%, and 79.0%, respectively. The CLint values of in vivo clearance were calculated to be 24.1, 21.6, 33.4, 10.7, 26.0 mL/min/kg, respectively. Thus its was speculated that ZG0128 was a medium clearance drug in humans, monkeys, and mice, a high clearance drug in dogs, and a low clearance drug in rats.

Using UPLC-UV/Q-TOF MS, the potential metabolites of ZG0128 in various liver microsomes (human, monkey, dog, rat and mouse liver microsomes) incubation systems were identified and the metabolic pathways of ZG0128 were speculated. The main metabolite of ZG0128 in above five types of liver microsomes were primary alcohol metabolite formed by morpholine ring-opening (M3). Other than small amounts of n-dealkylation metabolites M1 detected in monkey liver microsomes, there weren't any significant difference among the other types of liver microsomes.

Results from the liver microsome comparative studies showed that the primary metabolite formed in human liver microsomes was the morpholine ring-opening metabolite M3. The enzyme phenotype involved in the oxidation of ZG0128 to form M3 was identified with reaction phenotyping assay using chemical inhibition and human recombinant CYP450 enzymes.

Using UPLC/Q-TOF MS, the amount of ZG0128 and primary alcohol metabolite formed by morpholine ring-opening (M3) in human liver microsomes and different incubation systems for human recombinant enzymes, including CYP1A2, CYP2A6, CYP2B6, CYP2C8, CYP2C9, CYP2C19, CYP2D6, CYP2E1, CYP3A4, and CYP3A5, were determined. Results from the chemical inhibition assays showed that general P450 inhibitor 1-ABT, CYP3A inhibitor ketoconazole, and CYP2C8 inhibitor quercetin can significantly increase the amount of remaining parent compound in incubation system for human liver microsomes and inhibit the formation of M3.

Results from the human recombinant enzyme assay showed that CYP3A4 was the primary enzyme that produced M3 while CYP1A2, 2D6 and 2C9 were also partly involved. Results from the reaction phenotyping assay using chemical inhibition and recombinant CYP450 enzymes showed that the primary ZG0128 metabolite in human microsomes was a morpholine ring-opening metabolite catalyzed mainly by CYP3A4 and CYP1A2.

Inhibition and Induction of ZG0128 on CYP-P450s

The inhibition and induction potentials of ZG0128 on CYP-P450s were evaluated using mixed human liver microsomes and human primary hepatocytes as the in vitro models. Results showed that ZG0128 essentially had no inhibition on CYP1A2, CYP2B6, CYP2C8, CYP2C9, CYP2C19, CYP2D6, CYP3A4 (midazolam 1'-hydroxylation), and CYP3A4 (testosterone 6β-hydroxylation), with an IC50 greater than 100 mM. ZG0128 at concentrations of 0.10, 1.00, and 10.0 μM was incubated with human primary hepatocytes, and the changes in CYP1A2, CYP2B6, and CYP3A4 enzyme activities were measured to evaluate the induction potential for ZG0128 on

CYP enzymes. Results showed than ZG0128 had no in vitro induction on CYP1A2 and CYP3A4. The CYP2B6 enzyme activity was greater than 40% of the active control, but only for 1.00 μM ZG0128 with human primary hepatocytes batched HC4-15. Thus, the induction ability of ZG0128 on CYP2B6 was not fully clear.

Results from the evaluation of the inhibition and induction potential for ZG0128 on cytochrome P450s showed that ZG0128 had no inhibition on major CYP P450s enzymes. ZG0128 also had no in vitro induction on CYP1A2 and CYP3A4. The clinical induction potential on CYP2B6 required further investigation with plasma exposure.

We had fully studied the mechanism of action, preclinical efficacy, PK characteristics and safety characteristics of ZG0128. We intended to conduct a phase I dose escalation study evaluating the tolerability in healthy subjects. The following clinical trial protocol was designed based on requirements and guidelines of the CFDA, as well as other phase I clinical trials of similar products.

This trial was conducted in accordance with relevant principles found in the "Declaration of Helsinki", "The Drug Administration Law of the People's Republic of China", "Drug Registration Regulations", "Good Clinical Practice", "Guiding Principle for Administration on Phase I Clinical Trials on Drugs (For Trial Implementation) ", and "Guidelines for Laboratory Management of Biological Sample Analysis in Clinical Drug Trials (For Trial Implementation)". Under the premise of fully respecting subject rights, this trial was implemented by the Phase I Clinical Trial Laboratory of the First Hospital of Jilin University and Suzhou Haike Pharmaceutical Solutions Co., Ltd.

The clinical trial objectives, methodology, organization, data analysis, and trial management are described below.

Detailed Trial Design

Considerations for Basic Design

Based on the CFDA trial approval for jaktinib hydrochloride tablet, this trial aimed to evaluate the tolerability, pharmacokinetics, effects of food on pharmacokinetics, and metabolic transformation of oral, single- and multi-dose jaktinib in healthy subjects.

Based on the information above, the trial was designed as follows:

- Trial design: a single-center, randomized, double-blind, placebo-controlled, single- and multi-dose, dose-escalation study evaluating the effects of food on pharmacokinetics and metabolic transformation.
- Subjects: healthy males and females. The inclusion/exclusion criteria and criteria for exclusion from analysis were set according to guideline requirements issued by the CFDA.
- Key points of the trial: ensured subjects' compliance with medication, diet, and drinking water, conducted blood sampling according to a preset schedule, sample processing, and storage.
- Randomization: based on randomization number and gender, the statistician randomly allocated subjects in a 3:1 or 4:1 ratio into the treatment group or placebo group. Twelve subjects were randomly allocated to receive the administration either on an empty stomach or after a meal.
- Dosages: 25 mg, 50 mg, 100 mg, 150 mg, 200 mg, 250 mg, 300 mg, and 400 mg.
- Administration Method: orally, empty stomach or after a meal.
- Sampling method: collection of whole blood, anticoagulation with K2-EDTA, and separation of plasma.
- Analyte: original drug and metabolites (ZG0244 and ZG0245).
- Safety Evaluation: NCI CTCAE version 4.03 general evaluation criteria.

Study Procedures

Screening

Healthy subjects were screened for enrollment after the trial protocol was approved by the ethics committee of the study site. First, they were allowed to read the informed consent form for the study and were given sufficient time to consider. If they decided to participate in the study, the informed consent form had to be signed and screening procedures would be done before the study. After the informed consent form

was signed, the investigator would photocopy the identification card and fill out the demographic information of subjects.

Subjects would receive a series of examinations, including: medical history, medication history, surgical history, blood donation history, blood transfusion history, smoking and alcohol consumption history, physical examinations, vital signs, 12-Lead ECG, abdominal B-ultrasound, laboratory examinations (routine blood, routine urinalysis, stool analysis, biochemistry, and coagulation tests) and chest x-rays; infectious disease related tests include Hep B and C tests, syphilis and HIV tests, blood pregnancy tests (for women of childbearing age only), urine tests for recreational drugs (morphine, marijuana).

Subjects met all the inclusion criteria and none of the exclusion criteria could be enrolled in this study. Fourteen days prior to the start of the trial, the investigator would begin enrolling subjects based on the inclusion and exclusion criteria of the protocol. The investigator should keep all the screening records, record reasons for being ineligible, and detail any recent medication history.

Eligible subjects were admitted into the phase I hospital ward on the afternoon one day before the trial. Vital signs, alcohol and drug use, and pregnancy test (for woman of childbearing age) were reexamined to further verify the eligibility of subjects. Eligible subjects were randomly allocated and assigned a randomization number. Subjects then fasted for at least 10 hours before receiving the study drug. On the morning of the trial, subjects received the appropriate dose of jaktinib or placebo corresponding to their randomization numbers. Blood samples were collected before and after administration at the pre-scheduled times. Adverse events that occurred during the trial were recorded.

Single Ascending Dose (SAD)

Screening was done within 14 days prior to administration. After all examination results came out, a review was done to verify the eligibility of subjects. Eligible subjects were admitted to the phase I clinical trial laboratory of the First Hospital of Jilin

University on the afternoon of the day -1. After an overnight fast of at least 10 hours, subjects were given the study drug along with 240 mL of warm water on the first day (D1). PK blood samples were collected thereafter. Subjects must be hospitalized for 3 consecutive days. Subject must remain in a seated position with limited movement for 2 hours after administration. Subjects would receive a uniform lunch and dinner about 4 and 10 hours after the administration on D1, respectively (no breakfast). Water was not permitted within 1 hour before and 2 hours after administration. Medical staff would closely monitor and report adverse events through the entire trial.

SAD Trial Design

**Screening Period**	**Hospital Admission**	**Day of Administration**	**Observation Period**	**Trial Completion**
Screening	Admitted into Trial Site	Receive Dose	In Hospital	Tolerability Assessment, Discharge
D-14 - D-1	D-1	D1	D2 - D3	D3

SAD Day -1

Subjects were required to arrive at the study site to take following examinations on Day -1 (the day before administration):

Vital signs

Blood pregnancy test (women with childbearing age only)

Urine tests for recreational drugs (morphine, marijuana)

Breath alcohol test

Record of adverse events

The investigator would determine the subject's eligibility based on examination results from the screening period and available results on Day -1. Eligible subjects were randomized on the same day and assigned a randomization number.

SAD Day 1

After an overnight fast of at least 10 hours, subject would receive the study drug

along with 240 mL of warm water at about 8:00 in the morning (± 1 hour). Subjects should continue to ban water for 2 hours and fast for 4 hours after administration. Lunch and dinner were served about 4 and 10 hours after administration, respectively. Specific items that need to be completed are as follows:

PK blood sampling: 30 minutes before administration, and 0.25 h, 0.5 h, 1 h, 1.5 h, 2 h, 3 h, 4 h 5 h, 6 h, 8 h, 12 h, and 14 h after administration.

Vital Signs: within 1 h before the first dose, 2 h and 6 h after starting the dose.

Monitor and record adverse events.

SAD Day 2

No dose given. The following needs to be completed:

PK blood sample collection: 24 h and 36 h after the first dose

Vital sign measurements: 24 h after the first dose

Record adverse events.

SAD Day 3

The following needs to be completed before withdrawal:

PK blood sample collection: 48 h after the first dose

Laboratory tests: routine blood, blood biochemistry, coagulation tests, routine urinalysis, routine stool tests (include fecal occult blood);

12-Lead ECG;

Vital sign measurements: 48 h after the first dose

Physical examination;

Record adverse events.

Complete and withdraw from the trial

Multiple Ascending Dose Study (MAD)

MAD Study Design

SCREENING PERIOD	HOSPITAL ADMISSION	DOSE ADMINISTRATION (D1-D10)	OBSERVATION PERIOD	TRIAL COMPLETION

Screening	Admitted into Trial Site	Tolerance Assessment	Tolerance Assessment	In Hospital	Tolerability Assessment, Discharge
D-14 - D-1	D-1	D4	D7	D11	D12

MAD Day -1

Subjects were required to arrive at the study site to take following examinations on Day -1 (the day before administration):

Vital signs

Blood pregnancy test (women with childbearing age only)

Urine tests for recreational drugs (morphine, marijuana)

Breath alcohol test

Record of adverse events

The investigator would determine the subject's eligibility based on examination results from the screening period and available results on Day -1. Eligible subjects are randomized on the day and will each receive a randomization number.

MAD Day 1

After an overnight fast of at least 10 hours, subjects will receive the investigational drug with 240 mL of warm water. Subjects must not drink water for 2 hours and fast for 4 hours after dosing. Lunch and dinner were served 4 and 10 hours after administration, respectively. For q12h dosing, the dose is given approximately 2 hours after supper, at an interval of 12 hours from the previous dose. Subjects must not eat or drink for 2 hours after dosing.

Specific items that need to be completed are as follows:

PK blood sample collection: 30 minutes before dosing, and 0.25 h, 0.5 h, 1 h, 1.5 h, 2 h, 3 h, 4 h 5 h, 6 h, 8 h, 12 h after dosing

Vital sign measurements: within 1 h before dosing, 2 h and 6 h after dosing

Monitor and record adverse events.

MAD Days 2-9

The dose is administered once or twice daily.

After an overnight fast of at least 10 hours, subjects will receive the investigational drug with 240 mL of warm water. Take breakfast 0.5 h after dosing, take lunch 4 h after dosing, and take dinner 10 h after dosing; for q12h dosing, the dose is given approximately 2 hours after supper, at an interval of 12 hours from the previous dose. Subjects must not eat or drink for 2 hours after dosing.

The following needs to be completed:

PK blood sample collection: samples are collected at minimum plasma concentration on the morning of D2, D3, D4, D6, and D7 before dosing;

Laboratory tests (D4 and D7): routine blood test, blood biochemistry test, coagulation tests, routine urinalysis, and routine stool tests;

Symptoms and physical examinations: D4 and D7;

12-Lead ECG: D4 and D7;

Vital sign measurements: 1 hour before dosing on D2, D3, D4, and D7;

Record adverse events.

MAD Day 10

After a 10-hour fast, the final dose is given in the morning under the fasting condition.

The following must be completed:

PK blood sample collection: 30 minutes before dosing, 0.25 h, 0.5 h, 1 h, 1.5 h, 2 h, 3 h, 4 h 5 h, 6 h, 8 h, and 12 h after the final dose;

Clinical laboratory tests: routine blood test;

Vital sign measurements: within 1 h before the final dose, 2 h and 6 h after dosing

Record adverse events.

MAD Day 11

No dose given;

PK blood sample collection: 24 h and 36 h after the final dose;

Vital sign measurements: 24 h after the last dose;

Record adverse events.

MAD Day 12

The following must be completed before withdrawal:

PK blood sample collection: 48 h after the last dose;

Clinical laboratory tests;

Symptoms and physical examination;

12-Lead ECG;

Vital sign measurements: 48 h after the last dose;

Record adverse events.

Complete and withdraw from the trial

Food Effects and Metabolism Studies

The subjects are screened within 14 days prior to dose administration to determine whether subjects meet the inclusion criteria. Eligible subjects are admitted to the phase I clinical trial laboratory of the First Hospital of Jilin University on day -1 before the trial. After an overnight fast of at least 10 hours, subjects are given the investigational drug with 240 mL of warm water, either under the fasting condition or after a standard meal on D1 and D6. PK blood samples are collected thereafter.All subjects should be hospitalised for 8 days. Subject must remain in a sitting position allowing limited movement for 2 hours after receiving each dose. Subjects need to have lunch and have dinner together approximately 4 and 10 hours after dosing on D1 and D6. Water was not permitted within 1 hour before and 2 hours after administration. Medical staff would closely monitor and report adverse events through the entire trial.

Study Design for Effects of Food on Pharmacokinetics

SCREENING PERIOD	HOSPITAL ADMISSION	PERIOD 1 DAY OF ADMINISTRATION	PERIOD 1 OBSERVATION PERIOD	PERIOD 2 DAY OF ADMINISTRATION	PERIOD 2 OBSERVATION PERIOD	TRIAL COMPLETION ON DISCHARGE

D-14 - D-1	D-1	D1	D2-D6	D6	D7	D8

Day -1 (Admitted into Study Site)

Period 1 of evaluating the food effects on PK

Subjects were required to arrive at the study site to take following examinations on Day -1 (the day before administration):

Vital signs

Blood pregnancy test (women with childbearing age only)

Drug test (morphine and marijuana)

Breath alcohol test

Record of adverse events

The investigator would determine the subject's eligibility based on examination results from the screening period and available results on Day -1. Eligible subjects are randomized on the day and will each receive a randomization number.

Day 1 (Day 1 of Period 1)

After an overnight fast of at least 10 hours, subjects have to receive the investigational drug with 240 mL of warm water under the fasting conditions (Group A) or after a meal (Group B). Subjects should remain fasted for 4 hours after dosing. Lunch and dinner were served 4 and 10 hours after administration, respectively. Specific items that need to be completed are as follows:

PK blood sample collection: within 30 minutes prior to the first dose, 0.25 h, 0.5 h, 1 h, 1.5 h, 2 h, 3 h, 4 h, 5 h, 6 h, 8 h, 12 h, and 14 h after dosing

Urine PK collection (Group A, under the fasting conditions): prior to the first dose, 0-6 h, 6-12 h, 12-24 h

Feces PK collection (Group A, under the fasting conditions): collected during 0-24 h after dosing if subject has bowel movement

Vital sign measurements: within 1 h before dosing, 2 h and 6 h after dosing

Record of adverse events

Days 2-6 (Day 2-6 of Period 1)

The following must be completed on days 2-6 before entering Period 2:

PK blood sample collection (Group A in Period 1 under the fasting conditions): 24 h, 36 h, 48 h, 72 h, 96 h, and 120 h after the first dose

PK blood sample collection (Group B in Period 1 after standard meals): 24 h, 36 h, and 48 h after the first dose

Urine PK collection (Group A in Period 1 under the fasting conditions): 24-48 h, 48-72 h, 72-96 h, and 96-120 h

Feces PK collection (Group A in Period 1 under the fasting conditions): collected during 24-120 h after the first dose if subject has bowel movement

Vital sign measurements: 24 h, 48 h, 72 h, and 120 h after the first dose

Record adverse events.

Day 6 (Day 1 of Period 2)

After an overnight fast of at least 10 hours, subjects who have completed PK sample collections have to receive the investigational drug with 240 mL of warm water under the fasting conditions (Group B) or after a meal (Group A). Subjects should remain fasted for 4 hours after dosing. Lunch and dinner were served 4 and 10 hours after administration, respectively. Specific items that need to be completed are as follows:

PK blood sample collection: within 30 minutes prior to the second dose, and 0.25 h, 0.5 h, 1 h, 1.5 h, 2 h, 3 h, 4 h, 5 h, 6 h, 8 h, 12 h, and 14 h after dosing;

Vital sign measurements: within 1 h before the second dose, and 2 h and 6 h after dosing;

Record adverse events.

Day 7 (Day 2 of Period 2)

No dose given. The following must be completed:

PK blood sample collection: 24 h and 36 h after dosing in Period 2;

Vital sign measurements: 24 h and 48 h after dosing in Period 2;

Record adverse events;

Day 8 (Day 3 of Period 2)

No dose given. The following must be completed:

PK blood sample collection: 48 h after dosing in Period 2

Clinical laboratory tests (routine blood, blood biochemistry, coagulation tests, routine urinalysis, routine stool tests);

12-Lead ECG;

Vital sign measurements;

Symptoms and physical examination;

Record adverse events.

Complete and withdraw from the trial

PK Blood Sample Collection

Nomenclature of Biological Samples

Blood samples for determining the plasma concentration of jaktinib and its metabolites are collected at scheduled time points during the trial. Each sample is approximately 4 ml. Blood collection tubes and 2 cryogenic vials are serially numbered and affixed with dedicated labels before collecting blood samples. The label samples are as follows:

PK Blood Sample Label

SubjectID 01<br>PKTime dx-yh<br>Session 1A<br>ZGJAK001	SubjectID 01<br>PKTime dx-yh<br>Session 1B<br>ZGJAK001	SubjectID 01<br>PKTime dx-yh<br>Session 1<br>ZGJAK001

Urine PK Label

SubjectID 01<br>PKTime y-zh<br>Session 1UA<br>ZGJAK001	SubjectID 01<br>PKTime y-zh<br>Session 1UB<br>ZGJAK001

Feces PK Label

SubjectID 01<br>PKTime<br>ZGJAK001

For PK blood sample label, the first row is the subject's randomization number; the second row is the sampling time point, in which dx represents the corresponding test day, and yh represents the time points of PK sampling after dose administration; the third row is the type of sample tube: A is the blood sample inspection tube, B is the blood sample backup tube, no A and B is the blood collection tube. For urine PK label, the first row is the subject's randomization number; the y-z indicates the corresponding sampling time; the UA of the third row represents urine samples inspection tube, while UB represents urine samples backup tube. For feces PK label, the first row is the subject's randomization number; the second row indicates collection times. ZGJAK001 is the protocol number.

Biological Sample Collection and Processing

Any blood samples collection time beyond the time window (appendix 1) must be reported as a protocol deviation. Under this circumstance, pharmacokinetics and statistics should be analyzed by scheduled time and adjusted by actual collection time. Blood samples are collected by K_2-EDTA vacutainer, and placed in an ice bath to rapidly freeze the samples. The plasma is centrifuged (3000 rpm) at 2-8 ℃ for 10 min, and divided two parts into the one of 1.0 mL in sample inspection tube, and another one of remaining samples in backup tube. Blood samples should be centrifuged within 30 minutes and stored in the refrigerator within 1 hour. All blood samples are cryopreserved at -70 to -90 ℃. Sample inspection tubes are packaged by foamed polystyrene with sufficient dry ice to ensure frozen condition at least 24 hours. Samples should be delivered to the inspection unit in a timely manner. The blood samples are extracted and the plasma concentration of jaktinib hydrochloride is determined using the validated LC-MS/MS assay by sample inspection unit.

3 Biological Sample Transport and Cold Chain Logistics

At the end of the study, samples should be packaged with constant temperature, in which a thermometer will be used for real-time temperature monitoring to ensure frozen condition, and shipped by an express delivery company.

4 Subject Selection

Inclusion Criteria (Must Meet All Criteria to be Eligible)

1) Sign the informed consent form, and fully understand the content, procedure and possible adverse events before the trial starts;
2) Capable of completing the trial according to trial protocol;
3) Subjects (including male subjects) do not have birth plans in the next 6 months, and agree to adopt effective contraceptive measures. Refer to Appendix 6 for specific contraceptive measures;
4) Male and female subjects of 18-45 years of age (inclusive);
5) Male subjects must not weigh less than 50 kg, and female subjects must not weight less than 45 kg. (BMI) = weight (kg)/ height^2 (m^2), BMI between 18-28 (inclusive);
6) Health status: subjects do not have a clinically significant medical history related to the heart, liver, kidneys, digestive tract, nervous system, respiratory system, mental disorders or metabolic disorders;
7) Normal physical examination and vital signs, or abnormal physical examination and vital signs with no clinical significance.

Exclusion Criteria (Excluded if any criteria is met)

1) Smoke more than 5 cigarettes per day within 3 months before the trial starts;
2) Allergic to the investigational drug or its excipients, or allergies to various drugs and food;
3) Having a history of drug abuse and/or alcohol abuse (14 units of alcohol consumed per week: 1 unit = 285 mL of beer, 25 mL of liquor, or 100 mL ofwine);
4) Blood donation or massive blood loss (> 450 mL) within 3 months prior to the first

dose of investigational drug;

5) Having swallowing difficulty or a history of gastrointestinal disorders that may affect drug absorption;
6) Suffering from any disease that increases the risk of bleeding, such as hemorrhoids, acute gastritis, or gastric and duodenal ulcers;
7) Have taken any drugs that may affect liver enzyme activities 28 days prior to the first dose of investigational drug;
8) Have taken any prescription drugs, over-the-counter drugs, any vitamin products or herbal medicines within 14 days prior to the first dose of investigational drug;
9) Having a special diet (including dragon fruit, mango, grapefruit, and/or diet rich in xanthine) within 2 weeks prior to the trial, or intense physical activity, or other factors that may affect drug absorption, distribution, metabolism, and excretion;
10) Having concomitantly used the following inhibitors or inducers of CYP3A4, P-gp or Bcrp, such as itraconazole, ketoconazole, or dronedarone;
11) Have undergone major changes in diet or physical activity habits recently;
12) Have taken the investigational drug or participated in a clinical trial within 3 months prior to the trial;
13) Subjects who are unable to tolerate the high-fat meal (2 hard-boiled eggs of 100 g, 20 g of bacon, a pieces buttered toast of 50 g, 115 g of fries, 240 ml of whole milk) - only applicable to subjects participating in the postprandial study;
14) Abnormal ECG result with clinical significance;
15) Female subjects who are lactating, or who test positive for pregnancy during the screening or during the trial;
16) Clinical laboratory abnormalities with clinical significance, or other clinical findings indicating the following diseases with clinically significance (including but not limited to gastrointestinal, renal, hepatic, neural, hematological, endocrine, tumors, pulmonary, immune, psychiatric, or cerebrovascular diseases);
17) Positive hepatitis (including Hep B and Hep C), AIDS, and syphilis during

screening;

18) Acute disease during the screening or before the administration of investigational drug;
19) Have taken chocolate, any food or drink containing caffeine or xanthine 48 hours prior to the first dose of investigational drug;
20) Have taken any alcoholic products within 24 hours before dosing;
21) Subjects with positive alcohol and drug screening results, or subjects with a history of drug abuse in the past 5 years, or a history of narcotics within 3 months prior to the trial.

Rejection Criteria

The principal investigator should decide whether individual cases should be rejected from the statistical analysis of trial data. When one of the following situations occurs, the principal investigator should decide whether the subject should be rejected based on the factors such as the degree of completion of the trial and the reason for withdrawal, and provide a relevant explanation.

1) Subjects, who violate the inclusion/exclusion criteria, should not be included into the trial.
2) An adverse event occurs during the trial that results in the subject being unable to continue or complete the trial as scheduled.
3) Subject has poor compliance during the trial, such as subject does not receive the study drug, or samples can not
be collected according to the trial protocol for PK and safety evaluations.
4) During the trial, the investigator determines that the subject cannot continue to participate in the trial and actually terminates subject's participation in the trial.

Managing Subject Dropouts

(1) Determined by the Investigator

If the enrolled subjects are unsuitable to continue the trial, the investigator will

determine to ask the subject to withdraw from the study.

- The investigator believes there is a need to discontinue the study from an ethical standpoint.
- A serious adverse event (SAE) occurs, making the subject unsuitable to continue the trial.
- The investigator determines that withdrawal from the study is in the best interest of subject.
- Subject has poor compliance, including the following:

a. Subject does not take the medication or receive examinations as required;

b. Subject takes medications or food that will affect the safety assessment and the PK analysis results;

c. Subject is smoking or drinking (the investigator will determine whether subject needs to withdraw from the study);

d. Other behaviors of the subjects that may affect the results of the study.

(2) Subject voluntarily withdraws from the study

According to the informed consent form, subject reserves the right to withdraw from the study; or the subject does not withdraw the informed consent, but no longer accepts medication and examinations, and is lost to follow-up (also considered as withdrawal, or dropout). The reasons for the withdrawal should be recorded, and a safety assessment should be conducted. For subjects participating in the MAD study, PK blood samples should be collected at the scheduled time for the last dose.

(3) Processing

The reasons for withdrawal should be recorded based on the following: adverse event, lost to follow-up, protocol violation, death, or other. The investigator must make an effort to contact subjects who are lost to follow-up, with at least 3 phone calls attempts. If subjects prematurely withdraw from the trial, the following should be tested: vital signs, physical examinations, 12-Lead ECG, routine blood, blood biochemistry, routine urinalysis, and routine stool tests.

Criteria for Study Termination

1) According to NCI CTCAE version 4.03, if more than 1/3 of the subjects experience drug-related grade 3 non-hematologic adverse events or grade 4 hematologic adverse events, or 1 case experiences drug-related serious adverse event (SAE), then that suggests subjects cannot tolerate the drug.

2) During the trial, a major error is found in the clinical trial protocol that makes it difficult to evaluate the study drug.

3) The sponsor requests the premature termination of the study to fully ensure the rights and safety of the subjects.

4) The CFDA or ethics committee orders the premature termination of the study due to some reasons.

5 Trial Procedures

Administration

Single Dose:

On the morning of D1, under the fasting conditions, the subjects in 25 mg, 50 mg, 100 mg, 150 mg, 200 mg, 250 mg, 300 mg, and 400 mg dose groups are given 1 dose with 240 mL of warm water.

Multiple Dose:

For dose groups of 100 mg q24h, 150 mg q24h, 100 mg q12h, 200 mg q24h, and 150 mg q12h, the subjects are administered with 240 mL of warm water under the fasting condition on the morning of D1 and D10 (skip breakfast). On D2-D9, have breakfast 0.5 h after dose administration; have lunch 4 hours after administration, and have supper 10 hours after dose administration. q12h dosing means drug is given 2 hours after supper, at 12 hours intervals from the last dose.

The effects food on PK:

200 mg Dose Group or MTD Group: receive the investigational drug with 240 mL of warm water in the morning under the fasting condition or after the meal. Atotal

of 2 doses. Water was not permitted within 1 hour before and 2 hours after administration.

Fasting condition: after an overnight fast of at least 10 h, subjects need to receive the investigational drug with 240 mL of warm water according to the randomization list.

After the meal: after an overnight fast of at least 10 h, subjects are given a standard meal 30 min before the dose administration and finish the meal before dosing. The investigational drug is given according to the randomization list, along with 240 mL of warm water.

Standard Meal (High-Fat, 800-1000 kcal)

The postprandial study requires an overnight fast of at least 10 hours, followed by a standard meal 30 minutes before the dose. Fat content should be approximately 50% of the total caloric content, i.e., 800-1000 kcal breakfast, which must be finished before taking the dose. Detailed menu is as follows (refer to appendix 7):

1) 2 hard-boiled eggs;

2) 20 g bacon;

3) 1 slice of buttered toast;

4) 115 g fries;

5) 240 mL whole milk.

Investigational Drug Information

Jaktinib hydrochloride tablets: specification: 50 mg/tablet, lot number: 1703FP2157-01 (25 mg/tablet is 50 mg/tablet divided into halves, lot number: 1703ZG2157-01). Refer to product packaging for manufacture date. Manufactured by WuXi AppTec Co., Ltd. and provided by Suzhou Zelgen Biopharmaceuticals Co., Ltd.

Jaktinib hydrochloride placebo tablets: specification: 50 mg/tablet, lot number: 1703FP2158-01 (25 mg/tablet is 50 mg/tablet divided into halves, lot number: 1703ZG2158-01). Refer to product packaging for manufacture date. Manufactured by

WuXi AppTec Co., Ltd. and provided by Suzhou Zelgen Biopharmaceuticals Co., Ltd.

Formulation, Packaging, and Labeling

Jaktinib hydrochloride tablet (lot number: 1703FP2157-01) is an orange-red film-coated tablet, yellow without the coating. The tablet is composed of drug substance ZG0128 and the inactive ingredients of microcrystalline cellulose (101), croscarmellose, hypromellose (E15), magnesium stearate, and film coating premix (295F630005 orange). Jaktinib hydrochloride placebo tablet (1703FP2158-01) is consistent with the active drug in appearance, odor, and color. Investigational drugs are labeled according to GCP requirements.

The drugs are labeled in a uniform format. The package label of placebo contains the following information: clinical trial approval number, protocol number, medication number, name of clinical investigational drug (indicating for clinical trial use), dosage and administration, specifications, storage, lot number, expiration date, and sponsor. The package label of investigational drug contains the following information: clinical trial approval number, protocol number, medication number, name of clinical investigational drug (indicating for clinical trial use), dosage and administration, specifications, storage, lot number, expiration date, and sponsor.

Acceptance, Dispensing and Storage of Investigational Drugs

Drug Dispensing:

The investigational drug and placebo tablets are provided free of charge by Suzhou Zelgen Biopharmaceuticals Co., Ltd. and distributed to the study site as planned. Clinical trial personnel are responsible for safekeeping and dispensing the investigational drug, which should be locked up in a medicine cabinet. At the end of the trial, any unused or partially used medication, along with empty packaging, should be returned by the investigators to sponsor-designated monitor for destruction. The drug dispensing and retrieval should be recorded.

The investigational drugs should be dispensed by the investigator based on randomization numbers.

Storage:

The investigational drugs are stored, managed, and dispensed by clinical trial personnel. The director of study site should confirm the receipt of the study drugs in writing, and only use the study drugs within the framework of this clinical trial, as required in the protocol. The acceptance, distribution and return of investigational drugs should be documented in accordance with detailed procedures in the sponsor agreement. At the end of the trial, the sponsor will collect the packaging of the investigational drug.

The drug should be dispensed by a research group member. This member should ensure subjects are compliant with the dosing schedule, and document the date and quantity of dispensed and returned drug in the original medical records.

Administration Method

The investigator of phase I clinical trial distributes the investigational drugs for each phase of the trial according to the randomization list. Refer to Section 5.1 for details.

Dosage Determination

In a long-term toxicity study of 28-day continuous drug administration, the no-observed-adverse-effect-level (NOAEL) in beagles was 18.3 mg/kg. However, the NOAEL was 30 mg/kg in a long-term toxicity study of 26-week continuous drug administration in rats. The human equivalent dose (HED) for the above tests are 9 mg/kg and 5 mg/kg, respectively. According to the "Guidance for Estimating the Maximum Recommended Dose in Initial Clinical Trials for Therapeutics in Adult Healthy Volunteers" issued by CFDA and a safety factor of 10, the maximum safe starting doses for the first dose in human were calculated as about 50 mg and 30 mg, respectively.

The single starting dose of this study was set to 25 mg, while

the highest dose was set to 400 mg. The 400 mg dose would not be further increased even it was tolerated.

Administration Time

The scheduled administration time for each subject is 08:00 ± 1 h, with a 2 minute interval between subjects. Refer to the original clinical records for the actual administration time.

Blinding

This is a double-blind study. The investigators, project managers, monitors, pharmacokinetic analysts, data management are all blinded. The analysts will use blind analysis.

Other Drugs Used Before and During the Trial

During the trial, all drugs are not permitted except for the drug used to treat adverse events. Any drug used in the trial except the investigational drug should be documented in the original records and case report form, detailing the drug name, dosage, date of administration, and indication.

Drugs Not Permitted During the Trial

During the administration period, all drugs except the investigational drug should be discontinued.

In order not to affect the metabolism of the drug, the strong inhibitors and inducers of hepatic metabolizing enzymes should not be concomitantly used from 4 weeks (28 days) before dose administration until the end of the trial (inhibitors: allopurinol, amiodarone, chloramphenicol, chlorpromazine, cimetidine, ciprofloxacin, dextropropoxyphene, diltiazem, ethanol, erythromycin, imipramine, isoniazid, ketoconazole, metoprolol, metronidazole, miconazole, nortriptyline, oral contraceptives, oxyphenbutazone, perphenazine, phenylbutazone, primaquine, propranolol, quinidine, sodium valproate, sulfinpyrazone, sulfonamides, thioridazine,

trimethoprim, and verapamil; inducers: barbiturates, carbamazepine, aminoglutethimide, griseofulvin, meprobamate, phenytoin, glutethimide, rifampicin, and sulfinpyrazone). Closely observe the subject when CYP2C9 substrates with limited treatment (such as celecoxib, diclofenac, dronabinol, THC, phenytoin or fosphenytoin, piroxicam, sertraline, tolbutamide, topiramate, and warfain) are concomitantly used, so as to prevent serious adverse events.

Appropriate Use of Drugs during the Trial

The unexpected symptoms or adverse events arise during the trial should be judged by research physician. When a mild adverse reaction occurs during the trial, the subject should be closely monitored, but are generally not treated symptomatically so as to observe the degree of adverse reaction and its reversibility. However, a moderate or more severe adverse reaction should be treated symptomatically. The drug name, the route of administration, dosage, start and stop date should be documented in the original records and eCRF.

Contraindications and Restrictions

- Subjects enrolled in the single dose study are required to be hospitalised for3 days. Subjects enrolled in the multiple dose study and study of the effect of food on PK are required to be hospitalised for 12 and 8 days, respectively. Subjects may participate in normal indoor activities after dose administration. However, excessive activity or prolonged bed rest should be avoided;
- Subjects should not smoke or drink caffeinated or alcoholic beverages within the specified time period before the start of the trial (see exclusion criteria), and should not eat foods that affect the drug metabolism such as grapefruit. Non-compliant subjects may withdraw from the trial;
- Vaccines of any dosage form should not be used;
- Concomitant medications not outlined in the trial protocol are decided by the investigator based on a comprehensive evaluation of subjects' condition and the effect the trial results.

Medication Compliance

To fully inform subjects and improve medication compliance, the detailed description of the trial objective, basic information about the investigational drug, trial protocol, trial procedures, dosage regimen (such as dosage, route of administration, treatment cycles), clinical observation, frequency and process of sample collection, potential risks, reimbursement and compensation are provided during recruitment and screening. Before each dose, subjects' randomization number and the corresponding drug number, the dosage and administration sequence should be carefully verified. After dosing, the amount of the remaining study drug, the empty packaging and drug delivery device, and subjects' hands and mouth (oral medication) should be checked. If subjects need to use the washroom within 2 hours after dose administration, the investigator should accompany them to prevent subjects from hiding or spitting out the drug, and to prevent vomiting.

6 PK Parameters and Safety Assessment Indexes

PK Parameters for Evaluation and Safety Assessment Indexes

PK Parameters for Evaluation

Refer to Section 6.4 "Primary Pharmacokinetic Parameters" for details.

Safety Assessment Indexes

Include adverse events, serious adverse events, concomitant medications, changes from laboratory test (routine blood, blood biochemistry, routine urinalysis, etc.), clinical symptoms, vital sign measurements, 12-Lead ECG and physical examinations.

Assessment of Adverse Events and Serious Adverse Event

Definition of Adverse Events

Adverse event (AE) refers to any adverse medical event occurs after a subject signs the informed consent form, which does not necessarily have to have a causal relationship with the treatment. An AE can therefore be any unfavorable and/or

unintended sign, symptom, or disease temporally associated with the use of a medicinal product, whether or not considered related to the medicinal product. AE may also include pre- or post-treatment complications resulting from protocol-specific procedures, drug overdose, drug abuse/misuse, or occupational exposure. Any existing event that causes exacerbation or nature change of disease during the clinical study or by participating in the clinical study is also considered an AE.

AE does not include the following:

- Any pre-existing and stable disease and condition detected before the screening and visit, or abnormal laboratory test;
- Events other than adverse medical events (such as elective surgery, hospitalization due to social reasons and/or convenience);
- Any medical condition that occurs before signing the informed consent form and is not related to trial procedures, or any laboratory abnormality with clinical significance is not an AE, which is considered as a pre-existing condition.

Obtaining Adverse Event Information

The investigator should document any adverse events that is directly observed or self-reported by the subject in a clear and concise manner. In addition, subjects should be regularly asked regarding any adverse events after the trial begins.

6.1.3.3. Adverse Event Record

Adverse events should be documented during the trial, including the start date, severity, duration, measures taken, and outcome. Adverse events should be documented in the designated adverse events table of the case report form.

Adverse Event Severity Grading Scale

The severity of adverse events are graded according to the NCI CTCAE 4.03 criteria. Refer to the following criteria if an adverse event is not listed:

Grade I:	Mild, asymptomatic or mild symptoms, abnormal clinical or laboratory test only;

	no intervention indicated.
Grade II:	Moderate, minimal, local or noninvasive intervention indicated; limiting age-appropriate instrumental activities of daily living (ADL). ADL refers to preparing meals, shopping, using the telephone, etc.
Grade III:	Severe or medically significant but not immediately life-threatening; hospitalization or prolongation of hospitalization indicated; disabling; limiting self-care ADL. Self-care ADL refers to bathing, dressing and undressing, feeding self, using the toilet, taking medication, and not bedridden.
Level IV:	Life-threatening and requiring urgent treatment.
Level V:	Died of adverse events.

Criteria of Judgment on the Association Between Adverse Events and the Investigational Drug

According to the criteria for judging the causal relationship between the drug and adverse events, the correlation between adverse events and the application of the investigational product is divided into five levels, i.e. definitely associated, probably associated, possibly associated, possibly unassociated, definitely unassociated. Definitely associated, probably associated and possibly associated are all listed as adverse drug reactions. Take the total number of cases of adverse drug reactions as the numerator, and the total number of the included cases for adverse reaction evaluation as the denominator to calculate the incidence of adverse reactions.

Definitely associated: The adverse reaction conforms to the known reaction types of the suspected product and the reasonable time sequence after administration. The adverse event relieves or disappears after dose reduction or withdrawal, and re-appears after administration again.

Probably associated: The adverse reaction conforms to the known reaction types of the suspected product and the reasonable time sequence after administration. The adverse event relieves or disappears after dose reduction or withdrawal, but the event may also be attributable to the clinical state of the subject or other causes.

Possibly associated: The adverse reaction conforms to the known reaction types of the suspected product and the reasonable time sequence after administration. The adverse event relieves or turns unapparent after dose reduction or withdrawal, but the

event may be explained by the clinical state of the subject or other causes.

Possibly unassociated: The adverse reaction does not quite conform to the known reaction types of the suspected product or the reasonable time sequence after administration. The event may also be attributable to the clinical state of the subject or other causes.

Definitely unassociated: The adverse reaction does not conform to the known reaction types of the suspected product and the reasonable time sequence after administration. The event may be explained by the clinical state of the subject or other causes, and the event relieves or disappears after elimination of the clinical symptoms or other causes.

Evaluation of Clinical Laboratory Examination Abnormalities and Other Abnormalities Deemed as Adverse Events or SevereAdverse Events

Laboratory examination abnormalities and vital sign abnormalities without clinical significance shall not be recorded as AEorSAE. Laboratory examination abnormalities (such as clinical analysis, hematology and urine analysis) with clinical significance and other abnormalities (such as electrocardiogram, X ray, vital signs) must be recorded as AE and SAE;however, the above abnormalities discovered during screening shall be deemed as already existing before signing of the informed consent form, and shall not be recorded as AE. The abnormalities conforming to AE or SAE definitions must be recorded as AE or SAE. If the laboratory examination abnormality is part of any syndrome, the syndrome or diagnostic result (e.g. anemia) shall be recorded instead of the laboratory examination result (i.e. hemoglobin decrease).

Adverse Reaction Management

All clinical events and clinically significant laboratory examination adverse reactions shall be managed in accordance with the uniform guidelines specified in Common Terminology Criteria Adverse Events (CTCAE) Version 4.03 and Appendix 5. Clinical events and clinically significant laboratory examination abnormalities shall

be graded according to CTCAE 4.03. The investigators shall record the adverse reactions caused by the treatment, and shall remind and warn the medical monitors of the sponsor. The monitors shall discuss with the investigators and decide on appropriate actions. Regardless of the association with the treatment, all subjects with AE must be regularly monitored (if practicable) until symptoms subside, any abnormal laboratory values return to normal or baseline levels or considered irreversible, or until the observed changes can be properly explained. Level 3 or Level 4 laboratory examination abnormalities with clinical significance shall be confirmed by repeated tests if practicable, preferably within 3 working days after receiving the initial examination results.

Severe Adverse Events

Serious Adverse Event (SAE) refers to one of the following damaging reactions caused by the drug use: 1) causing death; 2) threatening life; 3) causing cancer, malformation or birth defect; 4) causing significant or permanent disability or damage of organ functions; 5) resulting in hospitalization or prolonged hospitalization (except for hospitalization due to medical insurance reimbursement, elective surgery or hospitalization without consent of the investigation physician); 6) causing other significant medical events which may result in the above situations if not treated.

Pregnancy Event

If any subject or spouse of the subject becomes pregnant or is found pregnant during the study, the investigators must fill in this information in Pregnancy Event Form and submit to the sponsor.

If the result of pregnancy meets the standard of SAE (such as spontaneous abortion, stillbirth, neonatal death or congenital malformation including aborted fetus, stillbirth or neonatal death), the investigators shall report as per the SEA reporting procedure.

Any neonatal death occurring within one month after birth shall be reported as SAE regardless of the cause of death. In addition, any infant death occurring one month

after birth which the investigators consider possibly associated with the investigational drug shall also be reported.

Management of Subjects with SAE

Any subject with SAE shall withdraw from the trial and receive active treatment.

Recording and Report of SAE

In case of SAE, the investigators shall fill in the Severe Adverse Event Report as detailed as possible with signatures and date. The investigators must report to the project leader appointed by the sponsor, Ethics Committee of the hospital, and China Food and Drug Administration (CFDA) within 24 hours after becoming aware of SAE. The initial report shall contain the following contents as far as possible: source of report, basic information of the subject, name of the investigational drug, name of the SAE, duration, severity, and association with the investigational drug, treatment and results of the event. Reporting Channel of Severe Adverse Events:

Reporting Channel of Severe Adverse Events

Ethics Committee of The First Affiliated Hospital of Jilin University		
Contact: Zhao Liyuan	Tel: 0431-88782013	Fax: 0431-88786015
Address: No. 71, Xin Min Street, Changchun, Jilin Province		P.O.: 130021
Suzhou Zelgen Biopharmaceuticals Co., Ltd.		
Contact: Wu Liqing	Tel: 0512-36681365	Mobile: 18015826715
E-mail：wulq_zelgen@163.com	Fax: 021-58382983	
Contact: Bi Hui	Tel: 021-58382983	Mobile: 18621697688
E-mail：bih_zelgen@163.com	Fax: 021-58382983	
Add: No. 209, Chen Feng Road, Kunshan, Jiangsu Province		P.O.: 215300
Research Supervision Office of Department of Drug and Cosmetics Registration of CFDA		010-8836 3228
Jilin Food and Drug Administration		0431-88905404
Medical Institutions Office of Medical Administration Department of National Health and Family Planning Commission		010-68792413

Trial Procedure Form

Single Administration Trial Procedure Form (25 mg, 50 mg, 100 mg, 150 mg, 200 mg, 250 mg, 300 mg, and 400 mg)

Study Procedures	Screening Visit[1]	Hospital Admission	Study Period		
	D-14 to D-1	D-1	D1	D2	D3
**Screening**					
Signing Informed Consent Form	X				
Demographic data	X				
Medical history, medication history and surgical history	X				
Normotopia chest x-ray film	X				
Abdominal color Doppler ultrasound	X				
Infection test[2]	X				
Breath alcohol test		X			
Urine tests for recreational drugs (morphine, marijuana)		X			
Blood pregnancy test	X	X			
Confirmation of inclusion/exclusion criteria	X	X			
Hospital admission		X			
Randomization		X			
**Tolerability Assessment**					
Laboratory examination[3]	X				X
Symptoms and physical examination	X				X
12-Lead ECG[4]	X				X
Vital signs[5]	X	X	X	X	X
Monitoring and record of adverse events		X	X	X	X
**PK Sampling[6]**					
Blood samples			X	X	X
**Dispensing of the Clinical Investigational Drug**					
Administration			X		
Withdrawal					X

Note:

1. Carry out subject screening within 14 days before the first administration (Day 1), and the screening procedures are the same for each group;
2. Infection test includes hepatitis b and c detection, HIV detection and syphilis spiral antibody detection;
3. Laboratory examinations include routine blood test, urine routine, blood biochemical routine, stool routine (including occult blood) and blood coagulation routine, and sampling of the examinations can take place within 48±2 h after the start of administration. If the subject produces no stool on the day of withdrawal, the routine

stool examination can be skipped, and the result within 3 days after the withdrawal (including the date of withdrawal) is acceptable as per the investigators' judgment.

4. 12-lead electrocardiogram examination time points: screening and withdrawal; and sampling of the examination can take place within 48±2 h after the start of administration;

5. The examination time points of vital signs are: screening period, hospitalization, within 1 h before first administration,2 h, 6 h, 24 h and 48 h after the start of administration. The collection time window is: within 1 h before administration, ±30 min within 4 h (including 4 h) after the start of admini stration, ±1 h within 4 -24 h(including24 h) after administration, and 2 h after more than 24 h after the start of administration;

6. The collection of PK blood samples shall take place within 30 min before administration, 0.25 h, 0.5 h, 1 h, 1.5 h, 2 h, 3 h, 4 h, 5 h, 6 h, 8 h, 12 h, 14 h, 24 h, 36 h and 48 h after administration.

Multiple Administration Trial Procedure Form

(100 mg q24h, 150 mg q24h, 100 mg q12h, 200 mg q24h, and 150 mg q12h dose group)

Study Procedures	Screening Visit[1]	Hospital Admission	Study Period											
	D-14 to D-1	D-1	D1	D2	D3	D4	D5	D6	D7	D8	D9	D10	D11	D12
**Screening**														
Informed Consent Form	X													
Demographics	X													
Medical history, medication history and surgical history	X													
Normotopia chest x-ray film	X													
Abdominal color Doppler ultrasound	X													
Infection test[2]	X													
Breath alcohol test		X												
Drug test		X[7]												
Blood pregnancy test	X	X												
Inclusion criteria	X	X												
Hospital admission		X												
Randomization		X												
**Tolerability Assessment**														
Laboratory examination[3]	X					X			X			X		X
Symptoms and physical examination	X					X			X					X

Electrocardiography[4]	X					X			X					X
Vital signs[5]	X	X	X	X	X	X			X			X	X	X
Adverse events		X	X	X	X	X	X	X	X	X	X	X	X	X
**PK Sampling[6]**														
Blood samples			X	X	X	X		X	X			X	X	X
**Dispensing of the Clinical Investigational Drug**														
Administration			X	X	X	X	X	X	X	X	X	X		
Withdrawal														X

Note:

1. Carry out subject screening within 14 days before the first administration (Day 1), and the screening procedures are the same for each group;
2. Infection test includes hepatitis b and c detection, HIV detection and syphilis spiral antibody detection;
3. Laboratory examinations: routine blood test, urine routine, blood biochemistry, stool routine (including occult blood) and blood coagulation routine during the screening period, on D4, D7 andD12 (samplingwithin72 h, 144 h and 264±2 h after first administration), and routine blood test on D10. Considering that the subject may not necessarily produce stool on the examination date during the trial, the stool routine examination time window during the trial is +3 days. If there is really no stool, the examination can be skipped, and theresults within 3 days after the withdrawal can be accepted as per the investigators' judgment.
4. 12-lead electrocardiogram examination time points are: screening period, D4, D7, and D12 (sampling within 72 h, 144 h and 264±2 h after first administration);
5. Vital sign examination time points are: screening period, hospitalization, within 1 h before first administration, 2 h and 6 h after the start of administration; within 1 h before administration on D2, D3, D4, D7 and D10; within 1 h before the last administration, 2 h, 6 h, 24 h and 48 h after the start of the last administration; The collecting time window is: within 1 hour before administration, ± 30 min within 4 h (including 4 h) after the start of administration, ±1 h within 4 -24 h (including 24 h) after the start of administration, and ±2 h after more than 24 h after the start of adm inistration;
6. PK blood sample collection: D1 - within 30 min before administration, 0.25 h, 0.5 h, 1 h, 1.5 h, 2 h, 3 h, 4 h, 5 h, 6 h, 8 h and 12 h after administration;30 min before morning administration on D2, D3, D4, D6 and D7; 30 min before administration and 0.25 h, 0.5 h, 1 h, 1.5 h, 2 h, 3 h, 4 h, 5 h, 6 h, 8 h, 12 h, 24 h, 36 h and 48 h after administration on D10.
7. Urine drug test (morphine and marijuana)

Trial Procedure Form of the Study on the Effect of Food on Pharmacokinetics

Study Procedures	Screening Visit[1]	Hospital Admission	Study Period							
	D-14 to D-1	D-1	D1	D2	D3	D4	D5	D6	D7	D8
**Screening**										
Signing Informed Consent Form	X									
Demographic data	X									
Medical history, medication history and surgical history	X									
Normotopia chest x-ray film	X									
Abdominal color Doppler ultrasound	X									
Infection test[2]	X									
Breath alcohol test		X								
Drug test		X[7]								

Blood pregnancy test	X	X								
Confirmation of inclusion/exclusion criteria	X	X								
Hospital admission		X								
Randomization		X								
**Tolerability Assessment**										
Laboratory examination[3]	X									X
Symptoms and physical examination	X									X
12-Lead ECG[4]	X									X
Vital signs[5]	X	X	X	X	X	X		X	X	X
Monitoring and record of adverse events		X	X	X	X	X	X	X	X	X
**PK Sampling[6]**										
Blood samples			X	X	X	X	X	X	X	X
Urine sample			X	X	X	X	X	X		
Stool sample			X	X	X	X	X	X		
**Dispensing of the Clinical Investigational Drug**										
Administration			X					X		
Balanced standard meal			X					X		
Withdrawal										X

1. Carry out subject screening within 14 days before the first administration (Day 1), and the screening procedures are the same for each group;
2. Infection test includes hepatitis b and c detection, HIV detection and syphilis spiral antibody detection;
3. Laboratory examinations include routine blood test, urine routine, blood biochemical routine, stool routine (including occult blood) and blood coagulation routine, and sampling of the examinations can take place within 168±2 hafter the start of administration. If the subject produces no stool on the day of withdrawal, the routine stool examination can be skipped, and the result within 3 days (including the date of withdrawal) after the withdrawal (including the date of withdrawal) is acceptable as per the investigators' judgment.
4. 12-lead electrocardiogram examination time points: screening and withdrawal; and sampling of the examination can take place within 168 ± 2 h after the start of administration;
5. Vital sign examination time points are: screening period, hospitalization, within 1 h after the first cycle of administration, 2 h, 6 h, 24 h, 48 h, 72 h and 120 h after the start of administration; 2 h, 6 h, 24 h and 48 h after the second cycle of administration; The sample collection time window is: within 1 h before administration, ± 30 min within 4 h (including 4 h) after the start of administration, ±1 h within 4 -24 h (including 24 h) after the start of administration, and ±2 h after more than 24 h after the start of administration;
6. PK blood sampling - the first cycle on empty stomach of Group A: within 30 min before administration, and 0.25 h, 0.5 h, 1 h, 1.5 h, 2 h, 3 h, 4 h, 5 h, 6 h, 8 h, 12 h, 14 h, 24 h, 36 h, 48 h, 72 h, 96 h and 120 hafter administration; Group A's second cycle after dining and Group B: within 30 min before administration, 0.25 h, 0.5 h, 1 h, 1.5 h, 2 h, 3 h, 4 h, 5 h, 6 h, 8 h, 12 h, 14 h, 24 h, 36 h and 48 h after administration.
PKurine sampling: before the first cycle on empty stomach of Group A, and at 0-6 h, 6-12 h, 12-24 h, 24-48 h, 48-72 h, 72-96 h, and 96-120 h;
Stool sampling: if and when the subject produces stool after administration in the first cycle on empty stomach in Group A (0-120 h)
7. Urine drug test (morphine and marijuana)

Sample Collection and Storage

See “Biological Sample Collection and Processing”.

Primary Pharmacokinetic Parameters

For plasma concentration data, adopt the software of Phoenix WinNolin (Pharsight Corporation, Version 6.4 and above) for estimation and analysis of pharmacokinetic parameters of the non-atrioventricular model and calculation of primary pharmacokinetic parameters, so as to fully reflect the characteristics of drug absorption, distribution, metabolism and excretion in the human body.

Single-dose PK parameters include:

- Time to peak (T_{max})
- Peak concentration (C_{max})
- Elimination half-life ($t_{1/2}$)
- Area under the plasma concentration-time curve at an interval between time 0 (first dose) and infinitely great ($AUC_{0-\infty}$)
- Area under the plasma concentration-time curve at an interval between time 0 (first dose) and 12 h (AUC_{0-12h})
- Area under the plasma concentration-time curve at an interval between time 0 (first dose) and t (AUC_{0-t})
- Amount of drug excreted in urine from time 0 to 120 h after administration (Ae_{0-120h})
- Amount of drug excreted in feces from time 0 to 120 h after administration (Ae_{0-120h})
- Cumulative excretion rate of the drug in feces and urine
- Oral clearance (CL/f)
- Renal clearance (CLr/f)

Steady-state pharmacokinetic parameters include:

- Time to peak (T_{max}, ss)
- Peak concentration (C_{max}, ss)
- Elimination half-life ($t_{1/2}$, ss)
- Area under the plasma concentration-time curve at an interval between time 0 (last dose) and t (AUC_{0-t}, ss)
- Area under the plasma concentration-time curve at an interval between time 0 (last dose) and infinitely great ($AUC_{0-\infty}$, ss)
- Oral clearance (CL/f, ss)
- Renal clearance (CLr/f, ss)
- Cumulative index: AUC_{0-24h}, ss on Day 10 / AUC_{0-24h} on Day 1;
- Fluctuation index: Fluctuation percentage at steady state = 100 • ($C_{max, ss}$ – $C_{min, ss}$) / $C_{avg, ss}$
- Minimum plasma concentration

As for those samples with concentrations lower than the lower limit of quantitation, during pharmacokinetic analysis, those sampled before reaching C_{max} should be calculated as zero while those after C_{max} should be calculated as ND (not detectable).

The subjects are grouped according to the dosage regimen. The results of main pharmacokinetic parameters are summarized by sample size, arithmetic mean, standard deviation, coefficient of variation, median value, minimum value, maximum value, geometric mean and geometric coefficient of variation.

The investigator should analyze the pharmacokinetic parameters and explain their clinical significance.

7 Quality Assurance

The sponsor and the investigator should establish their own quality assurance system, fulfill their duties, and strictly follow the clinical trial protocol to adopt the appropriate standard operating procedures, so as to ensure the implementation of quality control and quality assurance system in the clinical trial.

Quality Assurance on the Clinical Trial

Prior to the clinical trial, the investigator should receive trainings on the protocol, thereby gaining full understanding of the clinical trial protocol and all related parameters. The quality control personnel should ensure that the basic conditions of the clinical trial meet the standards specified in the protocol. During the trial, the investigator should carefully conduct the clinical operations according to the requirements of the institutional SOP and the clinical trial protocol, and record the raw data in a timely, integrated and standardized manner. The quality control personnel should verify the trial procedure and the original records for quality concern. After the termination of the trial, the study unit needs to collect the related documents, and archive them after the verification performed by quality control personnel. The quality assurance department of the clinical study unit should conduct permitted inspections over the ongoing trial. When a non-conformity is identified, the investigator and the person in charge of the site should be promptly notified and their corrections should be tracked.

Quality Assurance on Sample Testing

The laboratory conducting the analysis should establish a quality assurance system,

strictly follow the domestic and international standards of relevant technical guidelines, laboratory's SOP and quality control procedures for quality verification, formulate the verification plan and conduct it accordingly. The verification should include but not limited to: personnel training and authorization, sample management, standard substance management, instrument qualification, calibration and maintenance, method validation, sample testing, data verification, etc. The quality assurance personnel of the laboratory should conduct respective inspections on the analytical procedure, the test results and other aspects according to the testing progress and the results of verification performed by the quality control personnel.

Quality Assurance on Data Transfer, Calculation and Report

After the data from the study site is input into the (e)CRF, the quality control personnel should verify the consistency of the data in the (e)CRF with the original records to ensure the data in the (e)CRF is accurate. The monitor should thoroughly verify the integrity, accuracy and consistency of the trial data in the (e)CRF system against the original case history records. In case of items that are in doubt or inconsistent with the original records, the monitor should promptly raise the doubt, request the data entry clerk and the investigator to verify and correct the inconsistent data.

The personnel of data management department should verify the quality of entered data by edit check, and send queries of doubted results to the investigator for verification and correction. The quality control personnel should verify data management documents and data in the database.

The quality assurance personnel of the study site should conduct spot checks on the data transfer documents and statistical report data to ensure the accuracy of the data.

The sponsor should conduct respective inspections on the aforementioned clinical trial procedure, sample testing procedure, data, report and calculation procedure according to the testing progress and the results of verification performed by the quality control personnel/monitors.

8 Statistical Method and Sample Size Determination

Statistical Analysis

Study Population

The following study population would be selected for the analysis of the study data:

Full analysis set: Full analysis set includes the data of all subjects assigned to the treatment group and had taken drugs. Full analysis set is analyzed for compliance and baseline characteristics (demographics, medical history, medication history, concomitant medication and physical examinations).

Safety analysis set: Safety analysis set includes data of all subjects received the investigational drug at least once. The data is analyzed based on the actual treatment received.

Pharmacokinetic data analysis set (PKP):

Pharmacokinetic data analysis set refers to the data of subjects randomizedly grouped and had taken the investigational drug at least once with evaluable pharmacokinetic data. The data of subjects who have seriously violated the protocol should be excluded.

Statistical Analysis of Pharmacokinetic Parameters

The concentrations of jaktinib hydrochloride and its major metabolites in blood, urine and feces measured by the trial are calculated for pharmacokinetic parameters using the NCA module of WinNonlin. The main pharmacokinetic parameters include: AUC_{0-t}, $AUC_{0-t,ss}$, C_{max}, T_{max}, CL/F, V_d/F, $t_{1/2}$, etc.

Pharmacokinetic Analysis of Single-Dose

The study should be based on the PKP, which refers to the data of subjects randomizedly grouped and had taken the investigational drug at least once with evaluable pharmacokinetic data. The data of subjects who have seriously violated the protocol should be excluded, it will disturb the pharmacokinetic analysis if otherwise. Descriptive analysis is conducted on concentrations and parameters of pharmacokinetics based on the dose groups. The mean and individual concentration-time curves will be presented graphically. Individual and mean values would be presented in these graphs. In addition, individual and mean dose-normalized parameters would be plotted against the dose to assess whether the significant trend exists.

Pharmacokinetic Analysis of Multiple-Dose

The study should be based on the PKP, which refers to the data of subjects randomizedly grouped and had taken the investigational drug at least once with evaluable pharmacokinetic data. The data of subjects who have seriously violated the protocol should be excluded, it will disturb the pharmacokinetic analysis if otherwise. Descriptive analysis is conducted on multiple concentrations and parameters of pharmacokinetics based on the dose groups. The mean and individual concentration-time curves will be presented graphically. Individual and mean values would be presented in these graphs. The time to reach steady state is assessed. In addition, individual and mean dose-normalized parameters would be plotted against the dose to assess whether the significant trend exists.

Food effects on pharmacokinetics

The bioequivalence evaluation is conducted to find out whether food influences the bioavailability of Jaktinib hydrochloride tablets. The geometric mean and the 90% CI of C_{max} and AUC_{0-t} of Jaktinib hydrochloride tablets after the meal are compared with those under the fasting condition. If the 90% CI falls in 80.00%-125.00% of corresponding PK parameters under the fasting condition, it can be considered that there is no significant difference in main PK parameters between two methods of administration, i.e., food has no influence on PK of Jaktinib Hydrochloride Tablets. After logarithmic transformation of plasma concentrations, ANOVA with 2 x 2 cross-over design is conducted. The nonparametric test is used to calculate T_{max}. The significance level of all tests is 5%.

Drug Metabolism Analysis

The concentrations of the parent compound and its main metabolites in urine at different time periods after drug administration under fasting conditions are summarized in tables along with descriptive statistical results (number of subjects, arithmetic mean, standard deviation, coefficient of variation, minimum value, median, and maximum value). In addition, the urine PK parameters (Ae_{0-120}, $\%Ae_{0-120}$, $Ae_{0-\infty}$, and $\%Ae_{0-\infty}$) of Jaktinib hydrochloride and its metabolites are summarized with the descriptive method.

The concentrations of the parent compound and its main metabolites in feces at different time periods after drug administration under fasting conditions are summarized in tables along with descriptive statistical results.

The cumulative total amount of the parent compound over the administered dose is calculated.

8.1.3 Analysis of Safety Variables

Safety analysis (including demographic statistical data) includes the data of all subjects who had taken at least one dose of the investigational drug. The safety variables include the following items:

Adverse events observed by the investigator, self-reported by the subject or obtained from non-suggestive questions;

Laboratory safety evaluation results;

Clinical symptoms and vital sign measurement results;

Physical examination;

ECG;

The adverse events, the investigational drug discontinuation, and laboratory testing results of the safety evaluation population are summarized in tables.

Statistical description of quantitative data is carried out by mean, standard deviation, median, minimum value, and maximum value. Qualitative data is descriptively analyzed using frequency or number of subjects and percentages. The safety population is used for all safety analysis. The statistical analysis method is detailed in the clinical statistical analysis plan.

8.2 Sample Size Determination

The sample size determination for the trial is based on previous Phase I clinical trial rather than statistical considerations.

64 subjects are enrolled for the single dose trial, of which 48 receive the investigational drug and 16 receive the placebo. 50 subjects are enrolled for the multiple dose trial, of which 40 receive the investigational drug and 10 receive the placebo. According to the guidelines formulated by the CFDA, at least 12 subjects should be included in the food effect study. So in the trial, 12 subjects is enrolled for the study.

9 Management of Abnormalities during the Trial

Criteria for Study Termination

The clinical trial would be terminated if the criteria of trial termination set forth in Section

4.5 are met.

Risk Assessment and Risk Management

The medium- and long-term (24 weeks) administration with the similar drug Momelotinib in myelofibrosis patients may result in adverse events. The most common hematological adverse events are mostly Grade 1 and 2, while Grade 3 is mainly thrombocytopenia because the hemopoietic function of bone marrow is inhibited. Non-hematological adverse events include diarrhea, headaches, vertigo, abnormalities of laboratory biochemical tests, etc. However, these adverse events has low incidence and the patients can recover after symptomatic treatment. In addition, any drug first given to humans may occur allergic reactions. The allergic reaction treatment and risk management plan are detailed in Appendix 4 and 5. Closely monitor the subjects in various groups after drug administration for possible adverse events. Subjects must maintain a sitting position for 2 hours after administration and ambulation limited. Subjects must inform the duty staff if they need to get off the bed within 2 hours after administration.

Protocol Deviation or Violation

All requirements stipulated in the study protocol must be strictly implemented. Any intentional or unintentional deviation or violation of the study protocol and GCP principles will be regarded as protocol deviation or violation. Any protocol deviations should be recorded in the SOP Appendix of the study site and submitted to the statistical analyst after termination of the trial. Assessment should be carried out when serious protocol violations occur. If necessary, the sponsor can prematurely terminate the trial.

10 Protocol Revision History

NA

11 Data Processing and Record Retention

Requirements of Filling Data

1) For all subjects who signed the informed consent form, their information must be written down in the original medical record clearly and comprehensively. All items need to be completed (a horizontal line is applied if the information is NA).

2) All data in the original medical record must be checked to ensure no mistakes before it

is signed and dated.

3) Any corrections in the original medical record need to use strikethrough and put the revised content at the top, bottom, or side of the removed one. The reviser's name and the date should be stated. The correction fluid is not allowed.

4) Paste the original laboratory test report on the corresponding place in the original medical record.

5) Any data that is significantly high or outside the clinically acceptable range should be checked and the investigator need to provide the explanation.

Database Establishment and Data Entry

1) Database establishment: The corresponding EDC system is designed according to the study protocol and logic review conditions are set for entry, thereby a dedicated database is established for the trial. The EDC system must pass the validation test before the trial data is officially entered.

2) Data entry: The data entry clerk (a clinical research coordinator) who is adequately trained to operate the EDC system logins to the system and enters the data synchronously. Then the data monitor (a clinical research associate) will check every item against the source data and raise doubts on inconsistent results. The data entry clerk need to check these doubts against the original data one by one and correct them. In this way, the consistency between the data in the database and in the original records is ensured.

3) The data manager supervises the progress of data entry and data validation in a timely manner, and carry out quality inspection before locking database to ensure database quality.

12 Ethical Standards and Informed Consent

Ethical Standards

The trial protocol must be reviewed and approved by the Ethics Committee of The First Affiliated Hospital of Jilin University before implementation. The sponsor and the investigator must submit to the Ethics Committee the following materials: “Application Form of Ethical Review”, “Principal Investigator Resume and Qualifications”, “Sample of the Informed Consent Form”, "Clinical Trial Protocol", “Case Report Form (CRF)”, “Investigator’s Brochure” for clinicians, subject enrollment related documents, etc. The clinical trial must comply with the “Declaration of Helsinki", “Good Clinical Practice (GCP)” stipulated by

CFDA, and other relevant regulations. Getting approval from the Ethics Committee of The First Affiliated Hospital of Jilin University is a prerequisite to start the trial. Any modifications to the trial protocol made during the clinical trial must be approved or put on record by the Ethics Committee. The investigator is responsible for submitting the interim trial reports regularly according to requirements of the Ethics Committee. After termination of the trial, the investigator needs to inform the Ethics Committee immediately.

Informed Consent

Subjects must be fully informed before they participate in this trial in order to safeguard their legal rights. The investigator is responsible for giving complete and comprehensive information of the study objectives, study methods, function of the drug, anticipated benefit, potential toxicity reactions and possible risks to the subjects or their appointed representatives. In addition, the subjects should be fully aware of their rights, possible risks and benefits, and they need to be updated of any new information about the investigational drug. The subjects should be informed that participation in this clinical trial is entirely voluntary; they can withdraw from the trial unconditionally at any time during the trial and would not be punished in any way. The subjects also need to understand that the investigator and the sponsor have the right to access to, store, and conduct statistical analysis of the trial data according to the relevant regulations. The version and date of drafting or modification should be stated in the informed consent form. Subjects need to sign and date on the informed consent form after fully understanding the risk and benefits of this clinical trial, as well as potential adverse events before they participate in the trial. If some modifications were made to the study protocol during the trial, the corresponding modifications to the informed consent form should be made as well. The updated informed consent form should be reviewed and approved by the Ethics Committee, then re-signed by the subjects.

13 Study Report

After the study has ended, the study site should generate study reports based on the study results, which include the clinical trial report, method validation report, sample test report, and statistical analysis report. The format, content, attachments, spectra, etc., of the reports should follow the latest CTD format requirement in the draft guidance stipulated by the CFDA. The reports become effective after undergoing preparation, review, signing, and stamping. The

effective study report is submitted to the sponsor, and the latter will submit it to the CFDA for marketing authorization application.

14 Data Retention

In order to ensure the assessment and supervision of the clinical trial by the CFDA and the sponsor, the investigator should agree to store all study data including the original inpatient records of the subjects, informed consent forms, case report forms, and detailed records of drug distribution. The hardcopy and electronic documents generated by the study site and the sample testing laboratory should be properly stored to ensure traceability in a timely manner. The investigator must retain the data for at least 5 years after the end of the trial. The sponsor must retain the clinical trial data for at least 5 years after the investigational drug is approved for marketing. Suzhou Zelgen Biopharmaceuticals Co., Ltd. is in possession of all data of the clinical trial. Documents from the study site should not be damaged without a written agreement between the investigator and the sponsor. The sponsor must be informed if the investigator wants to provide the trial documents to another party or transfer to other places. Unless requested by the CFDA, the investigator is not allowed to provide trial information in any form to the third party without the written consent of the sponsor.

15 Study Site

Clinical Trial Institution: Phase I Clinical Trial Laboratory of the First Hospital of Jilin University

Address: No. 71, Xin Min Street, Changchun, Jilin Province

Principal Investigator: Ding Yanhua

Sponsor: Suzhou Zelgen Biopharmaceuticals Co., Ltd.

Address: No. 209, Chengfeng Road, Kunshan City, Suzhou, Jiangsu

Person in Charge of Clinical Trial: Wu Liqing

Pharmacokinetic Sample Testing Agency: Suzhou Haike Pharmaceutical Technology Co., Ltd.

Address: Room 1113, Block D, No. 398 Ruoshui Road, Suzhou Industrial Park, Suzhou, Jiangsu

References

[1] CFDA. Guidelines for Bioavailability and Bioequivalence Studies of Pharmaceuticals in Humans. 2005. 3.

[2] CFDA. Guidelines for Pharmacokinetic Studies of Pharmaceuticals. 2005.

[3] CFDA. Good Clinical Practice. 2003.

[4] CFDA. Drug Registration Regulations. 2007.

[5] Verstovsek S, Mesa RA, Gotlib J, et al. A double-blind, placebo-controlled trial of Ruxolitinib for myelofibrosis [J].N Eng J Med 2012,366(9):799–807.

[6] Harrison C, Kiladjian JJ, Al-Ali HK, et al. JAK inhibition with Ruxolitinib versus best available therapy for myelofibrosis[J].N Eng J Med 2012;366(9):787-798.

[7] Verstovsek S, Mesa RA, Gotlib J, et al. Consistent benefit of ruxolitinib over placebo in spleen volume reduction and symptom improvement across subgroups and overall survival advantage: results from COMFORT-I. Blood,2011;118:abstract 278

[8] Harrison C, Kiladjian JJ, Gisslinger H,et al. Ruxolitinib provides reductions in splenomegaly across subgroups: an analysis of spleen response in the COMFORT-II study.Blood 2011;118:abstract 279

[9] JAKAVIR the first medication to receive Health Canada approval to treat patients with myelofibrosis [News release].Canadian Newswire Web site. Available from: http://www.newswire.ca/en/story/1003655/-pr-jakavi-the-first-medicationto-receive-health-canada-approval-totreat-patients-with-myelofibrosis.2012.[Accessed28 August 2012]

[11] Verstovsek S, Kantar Jian H, Mesa Ramjet al. Safety and efficacy of INCB018424, a JAK1 and JAK2 inhibitor, in myelofibrosis[J]. N Engl J Med 2010; 363:1117-27

Appendix 1: Deviation of Blood Collection Time Points

In this study, blood samples should be collected within the allowed time frame.

Allowable time deviation during blood collection: When the time interval of sampling points was less than or equal to 1 h, the time deviation should be within ± 5%; when the time interval of sampling points was greater than 1 h, the time deviation should be within ± 2.5%. See the following table for specific requirements for the sampling time. Blood sample for PK study should be collected 30 minutes before dosing.

Any blood collection outside the allowed time frame should be immediately recorded in the relevant source data table. During multiple blood collection, the indwelling catheter will be treated with 5 mL physiological saline and retained on the forearm vein. So the residual blood containing physiological saline must be removed before blood collection. Disposable sterile syringes and needles are used for blood collection.

Deviation of Blood Collection Time Points

Sampling point (h)	Time Deviation of Blood Collection (±min)
0	30 min before dosing
0.25	0.75
0.5	0.75
1	1.5
1.5	1.5
2	1.5
3	3
4	3
5	3
6	3
8	3
12	6
14	3
24	15
36	18
48	18
72	36
96	36
120	36

Appendix 2: Clinical Laboratory and Imaging Tests

**Biochemical Tests** *(check the following during screening and before completing the trial:)*	**Complete Blood Count** *(check the following during screening and before completing the trial:)*
Direct bilirubin (DBIL)<br>Indirect bilirubin (IBIL)<br>Total bilirubin (TBIL)<br>Urea (BUN)<br>Creatinine (Cr)<br>Aspartate Aminotransferase (AST)<br>Alanine Aminotransferase (ALT)<br>Gamma-Glutamyl Transferase (GGT)<br>Alkaline Phosphatase (AKP)<br>Total Protein (TP)<br>Albumin (ALB)<br>Fasting Glucose (GLU)<br>Serum Amylase (AMY)<br>Serum Lipase<br>Creatine Kinase (CK)<br>Lactate Dehydrogenase (LDH)<br>Creatine Kinase Isoenzyme<br>α-Hydroxybutyrate Dehydrogenase<br>Triglyceride (TG)<br>Cholesterol (CHOL)<br>HDL-C<br>LDL-C<br>Cholinesterase (CHE)<br>Globulin<br>Albumin/Globulin Ratio<br>Total Bile Acid<br>Prealbumin<br>Potassium (K+); Sodium (Na+); Chlorine (Cl-); Calcium (Ca2+)<br>Inorganic Phosphorus (P)<br>Carbon Dioxide Combining Power<br>Uric Acid (UA)	White Blood Cell (WBC);<br>Neutrophil Percentage (NE%);<br>Lymphocytes Percentage (LY%);<br>Monocytes Percentage (MO%);<br>Eosinophils Percentage (EO%);<br>Basophils Percentage (BA%);<br>Absolute Neutrophil Count (NE#);<br>Absolute Lymphocyte Count (LY#);<br>Absolute Monocyte Count (MO#);<br>Absolute Eosinophil Count (EO#);<br>Absolute Basophil Count (BA#);<br>Red Blood Cell (RBC);<br>Hemoglobin (HGB);<br>Hematocrit (HCT);<br>Mean Corpuscular Volume (MCV);<br>Mean Corpuscular Hemoglobin (MCH);<br>Mean Corpuscular Hemoglobin Concentration (MCHC);<br>Red Cell Distribution Width (RDW);<br>Platelets (PLT);<br>Plateletcrit (PCT);<br>Mean Platelet Volume (MPV);<br>Platelet Distribution Width (PDW).
**Serum Pregnancy Test**** (Females of Child Bearing Age Only)<br>*(test during screen and on admission)*	**Routine Urinalysis**<br>*(check the following during screening and before completing the trial:)*<br>Urine glucose (GLU), urine bilirubin (BIL), urinary

**Routine Stool Test:**<br>Color and shape<br>Red blood cells<br>White blood cells<br>Fecal occult blood	occult blood (BLD);<br>Urine proteins (PRO), urine ketones (KET), urine nitrite (NIT);<br>Specific gravity (SG); pH;<br>Urobilinogen (UBG);<br>Leukocyte esterase (LE);<br>Vitamin C (Vc);<br>Red blood cell count (RBC);<br>White blood cell count (WBC);<br>Epithelial cell count (EC);<br>Urinary cast count (CAST);<br>Bacterium count (BACT);<br>Small round cell (SRC);<br>Yeast-like cell (YLC);<br>Urine crystal (XTAL)
**Breath Alcohol Test**<br>*(test on admission)*	
**Substance Abuse Screening**<br>*(test on admission)*<br>Marijuana and morphine content in urine	
Infectious diseases<br>*(check the following during screening only:)*<br>Hep B (HBsAg, anti-HBs, HBeAg, anti-HBe, anti-HBc)<br>Hep C antibodies<br>HIV<br>TP-Ab	**Coagulation Tests**:<br>*(check the following during screening and before completing the trial:)*<br>Thrombin time (TT)<br>Activated partial thromboplastin time (APTT)<br>Prothrombin time (PT)<br>International normalized ratio (INR)
X-Ray Examination<br>*(check the following during screening only:)*<br>Normotopia chest x-ray film<br>**Abdominal** Ultrasound<br>*(check the following during screening only:)*<br>Hepatobiliary and pancreas	12-Lead ECG<br>*(during screening and before completing the trial:)*

Appendix 3: Creatinine Clearance Formula

Ccr=[(140-age) x weight (kg)]/[0.818 x Scr(umol/L)] Pay attention to the unit of creatinine during calculation; for females, results x 0.85.

Appendix 4: Anaphylactic Shock Treatment Plan

Anaphylactic shock is caused by the interaction of antigenic substances (such as drugs, heterologous proteins, animals and plants) with the corresponding antibodies, which can cause a wide range of type I hypersensitivity reactions. The release of histamine, bradykinin, serotonin and platelet-activating factors, leads to vasodilation and increased vascular permeability. Fluid leaks into the interstitial space, resulting in rapid decline in circulating blood volume, causing shock. Anaphylaxis must be treated decisively and timely. Treatment is as follows:

(1) Immediately stop or remove the allergen. Slow down the absorption of the allergen. Ligating a tourniquet above the injection site if the antigen had been administered in the arm in a skin test.

(2) Epinephrine: Administer epinephrine intramuscularly, ideally at the site of the original injection to slow down the absorption of the allergen.

Epinephrine dosage: 0.1% epinephrine administered subcutaneously or intramuscularly, 0.5-1.0 ml per dose for adults, 0.02-0.25 ml/kg per dose for children. For severe cases, administer 1/2-1/3 of the intramuscular dose diluted in 40-50 ml of 50% dextrose solution intravenously. For cardiac arrest, use 1 ml of 0.1% epinephrine for intracardiac injection and perform chest compressions. When the effects of epinephrine is short-lived, for example, the subject does not respond to the first dose, repeat in 10-15 minutes, or use 1-2 mg epinephrine diluted with 100-200 ml of 5% dextrose through IV drip.

(3) Adrenocortical hormone: Adults can receive IV bolus of 100-300 mg hydrocortisone diluted in 30-40 ml of 5% dextrose. Or IV push of an equivalent dose of 5-750 mg of dexamethasone diluted in 20-40 ml of 5% dextrose solution can be adopted. Repeat in 1-3 hours if needed.

(4) Fluid replacement to recover and maintain adequate blood volume is an important step in the treatment of anaphylactic shock. In general, intravenous infusion of 5% dextrose in normal saline is used. Not only can it provide the necessary fluid and calories, but also maintain a rapid and effective administration route. Plasma and serum albumin can also be infused. Vasoactive drugs can be used. For example, an IV drip of 50-100 mg metaraminol diluted in 500 ml of 5% dextrose can be used alone, or together with dopamine if needed. Rehydration therapy for adults can generally reach 4000 ml

on the first day (can increase or decrease depending on urine volume). If the treatment response is poor, central venous pressure (CVP) can be measured to guide treatment.

(5) Administer oxygen and maintain airway: If a laryngeal edema obstructs breathing or there is an inspiratory dyspnea, tracheotomy should be performed immediately.

(6) Antihistamines: 25-50 mg IM promethazine; 4-8 mg oral chlorpheniramine; 25-50 mg oral diphenhydramine.

(7) Other treatments: quick acupuncture of GV-26 (Shui Gou), bilateral PC-6 (Nei Guan), LI-4 (He Gu) and KI-1 (Yong Quan) and moxibustion of DU-20 (Bai Hui), CV-6 (Qi Hai), and BL-44 (Shen Tang). Specific treatments may be used for different allergens. For example, 10-20 ml of 10% calcium gluconate can be given intravenously for penicillin allergy, or streptomycin, gentamicin and other aminoglycoside allergies.

In general, the later the symptoms appear after the exposure to the allergen, the better the prognosis tends to be. Oftentimes, patients with severe hypersensitivity reactions or who have "lightning quick" anaphylactic shock suffered poor prognoses. Patients with coronary heart disease are prone to myocardial infarction. Patients with nervous system symptoms may be prone to carries over various complications caused by hypoxia after prominent improvement of symptoms.

Appendix 5: Risk Management Plan

The "Risk Management Plan" is primarily used to identify, describe, prevent, or minimize drug-related risks, and also evaluate the effectiveness of interventions taken.

The interventions in the "Risk Management Plan" are risk minimization measures for investigators and relevant personnel to use as a reference only. It is supplementary information not included in the clinical trial protocol, and should not be used as a guidance for dose adjustment and determining adverse reactions. Please consult a specialist for specific treatment strategies.

Ensuring Subjects Are Informed

The investigator must provide information regarding this trial both in oral and written form. Subjects, guardians and legal representatives (if necessary) have the right to know all details regarding this trial.

The informed consent form (along with the trial protocol) must be submitted to the ethics committee for review and approval. If necessary, the investigator has the responsibility to explain the contents of the informed consent form using a manner and wording that subjects can understand. Subjects and representatives must have sufficient time to read the informed consent form prior to signing it.

The final informed consent form should include the following: purpose of the trial, process and duration of the trial, examinations and procedures, potential benefits and risks, the different intervention groups that subjects may be assigned to; the treatment and corresponding compensation available to subjects in the event of harm to subjects associated with the trial; the confidentiality nature of subjects' personal information.

The informed consent form must be signed and dated by the subject (or subject's legal representative). The investigator should also sign and date the informed consent form. The form should also be signed by an independent witness who can prove that the subject has agreed to participate in the trial. A copy of the informed consent form should be kept by the investigator and one by the subject. If important new information is discovered regarding the investigational drug, the informed consent form must be revised and submitted to the ethics committee for approval, after which informed consent must be obtained again from the subject.

Subject Selection: Jaktinib Hydrochloride Tablets Applied To Human Without Precedent

In the randomized, double-blind, dose escalation trial, healthy subjects are enrolled to receive single- and multiple-dose jaktinib hydrochloride tablets or placebo to investigate the tablet's tolerability, pharmacokinetics and food-related influence (Protocol Number ZGJAK001).

JAK-STAT (Janus Kinase-Signal Tranducer and Activator of Trancription) pathway is a key signaling pathway in the cell. This signaling cascade communicates information from chemical signals outside the cell to the nucleus to control gene expression. Various cytokines and growth factors utilize the JAK-STAT pathway to mediate physiological processes, such as hematopoiesis and immune responses.

Jaktinib is a JAK1 and JAK2 inhibitor. Preclinical toxicology studies show that jaktinib has good tolerability. No irreversible, serious adverse reactions occurred at standard doses. Like other similar medications, the most common adverse effects for jaktinib include: thrombocytopenia, neutropenia, anemia, nausea, diarrhea, headache, dizziness, fatigue, and shortness of breath (dyspnea). Non-hematologic toxicities are mild and infrequent. Hematologic toxicities are primarily mild to moderate, and can be relieved by dose reduction or treatment suspension.

To minimize subject risks, treatment time was shortenedmaximumly and scientifically. All subjects will receive the medication for no more than 10 days.

This trial is carried out in a clinical institution of national standards. It has a strict medical management system, complete first aid measures and accident management SOP to ensure the safety of subjects.

In summary, we believe that it is safe and reasonable to conduct single- and multiple-dose escalation trials in healthy subjects.

The starting dose is set as 25 mg in this dose escalation study. Since subjects enrolled in this trial are healthy volunteers, jaktinib 400 mg dose group is proposed as the tentative maximum dose for the single- dose trial. The doses given in the multiple ascending dose study include 100 mg q24h,

150 mg q24h, 100 q12h, 200 mg q24h, and 150 q12h.

In the single ascending dose study, eligible subjects are enrolled randomly to a certain dose group. Each dose group contains 8 subjects (jaktinib hydrochloride vs. placebo ratio = 3:1). In multiple ascending dose study, eligible subjects are enrolled randomly to a certain dose group to receive either jaktinib or placebo. Each dose group contains 10 subjects (jaktinib hydrochloride vs. placebo ratio = 4:1).

Dose Escalation Requirements

The intact observation period of the previous dose group must be completed first, and if no more than 1/3 of subjects in a dose group show drug-related grade 3 non-hematologic or grade 4 hematologic adverse events (NCI CTCAE version 4.03) or if there was no drug-related serious adverse event, then it shows subjects tolerate the medication at that dose, and the trial can move on to the next dosage level. Each subject may only participate in one dosage level. If the MTD is not observed even in the high dose group, then the trial can be terminated.

Withdrawal Criteria

To ensure the safety of subjects, the investigator may determine that it is unsuitable for the subject to continue participating in the trial, and thus asks the subject to withdraw from the study.

- The investigator believes there is a need to discontinue the study from an ethical standpoint.
- A serious adverse event (SAE) occurs, making the subject unsuitable to continue the trial.
- The investigator determines that withdrawal from the study is in the best interest of subject.
- Subject has poor compliance, including the following:

a. Subject does not take the medication or receive examinations as required;

b. Subject takes medications or food that will affect the safety assessment and the PK analysis results;

c. Subject is smoking or drinking (the investigator will determine whether subject needs to withdraw from the study);

d. Other behaviors of the subjects that may affect the results of the study.

Subject voluntarily withdraws from the study

According to the informed consent form, subjects reserve the right to withdraw from the trial at any time for any reason.

Criteria for Trial Termination:

1) According to NCI CTCAE version 4.03, if more than 1/3 of the subjects experience drug-related grade 3 non-hematologic adverse events or grade 4 hematologic adverse events, or 1 case of drug-related serious adverse event (SAE) occurs, then that suggests subjects cannot tolerate the drug.

2) During the trial, a major error is found in the clinical trial protocol that makes it difficult to evaluate the study drug.

3) The sponsor requests the premature termination of the study to fully ensure the rights and safety of the subjects.

4) The CFDA or ethics committee orders the premature termination of the study due to some reasons.

Determining and SAE and Submitting SAR

If a SAE occurs during the trial, the investigator must fill out the serious adverse report (SAR), and submit it to the pharmaceutical supervisory and administrative department, the department of public health administration, the sponsor, and the ethics committee within 24 hours. The investigator must sign and date the report.

Once a SAE occurs, the sponsor and the investigator should promptly investigate the event and report it to relevant agencies based on GCP requirements. The sponsor should provide the investigator with legal and economic guarantees, and provide the subject with corresponding treatment and other expenses, or corresponding financial compensation, for SAE which may be associated with the trial or investigational product;

Appendix 6: Contraceptive Measures, Definition of Women of Childbearing Age, and Contraception Requirements

1. Background

In animal studies, jaktinib, at dosages with no obvious toxicity observed, did not show adverse effects on fertility or embryonic development. However, no clinical trials of jaktinib has been conducted in pregnant women, so its effects on human fetus and pregnancy is not yet clear. There is currently no relevant human-related data for reference. Refer to "Jaktinib Hydrochloride Investigator Manual" version 1.0 for details

2. Definition of women of child-bearing age

Women > 54 years of age with no menstrual period ≥ 12 months, or women of any age who have undergone hysterectomy or bilateral oophorectomy or clinically diagnosed with ovarian failure are not considered women of child-bearing age.

Women ≤ 54 years of age (including women with any duration of amenorrhea) without hysterectomy or bilateral oophorectomy or clinically diagnosis of ovarian failure are considered women of child-bearing age.

3. Contraception requirements for women of child-bearing age (and their male partners)

Prior to enrollment, female subjects of child-bearing age must test negative for serum pregnancy during screening and the visit on Day -1. Starting from 3 weeks prior to dose administration to 6 months after the last dose, subjects must agree to the following.

·Sexual restraint . Regular sexual restraint is not allowed (such as calendar method, ovulation method, symptom-body temperature method, and post-ovulation method). From date of screening to 6 months after the last dose, in addition to the proper use of the condom by the male partner, women of child-bearing age must adhere to the correct use of one of the following contraceptive methods:

- Intrauterine device (IUD) with annual failure rate < 1%
- Female barrier: a cervical cap or uterine cap with spermicide
- Tubal sterilization
- Vasectomy of the male partner
- Vaginal ring

4. Contraceptive requirements for male subjects (and their female partners)

Within 6 months after the last dose, all male subjects must agree to adhere to the proper use of condoms. If their female partner is of child-bearing age (see definition above), one of the contraceptive methods described above must be used from the date of screening to 6 months after the last dose.

5. Male subjects must agree to avoid donating sperm within 6 months after the last dose.

6. The following procedures should be taken if pregnancy takes place

If subjects (or their partners) become pregnancy during the trial, or within 6 months after the last dose, then the investigator should be notified. Subjects who are pregnant or suspected of being pregnant should immediately notify the investigator and suspend receiving the study drug. Subjects' partners who are pregnant or pregnant subjects must immediately notify the investigator.

Appendix 7: Standard Western Meal (800-1000 kcal) Ingredients, Preparation Methodsand Calories

Western standard meal ingredients and preparation methods

Ingredients	Portions Per Person
2 Hard-Boiled Eggs: 100 g	2 eggs per person, (100 ± 10) g
Butter: 20 g	Each box 10 g, 2 boxes per person (20 ± 2) g
Bacon: 20 g	(20 ± 2) g per person
Toast: 50 g	1 slice per person, (50 ± 5) g
Fries: 115 g (After Deep-Frying)	(115 ± 12) g per person
Whole Milk: 240 ml	1 cup per person (240 ± 10) ml

Preparation methods:

1. Butter: No need to weigh, directly spread over the toast.
2. Fries shall be crispy. Weigh 115 g after frying, sprinkle small amounts of salt , and then put them on atray.
3. Bacon: Heat with microwave and place it on toast.

Western standard meal ingredient calories

Food Categories	Carbohydrate Weight (g)	Fat Weight (g)	Protein Weight (g)
Hard-Boiled Egg	0.10/100 g	10.50/100 g	12.10/100 g
Butter	0	80.00/100 g	0.7/100 g
Bacon	0	19.0/100 g	19.1/100 g
Toast	48.7/100 g	2.8/100 g	9.9/100 g
Fries	24.4/100 g	5.9/100 g	2.6/100 g
Cooking Oil	0.10/100 g	99.80/100 g	0
Whole Milk	5.0/100 ml	3.6/100 ml	3.1/100 ml

Calorific values of 1 western standard meal

Food categories (Weight)	Carbohydrate (g)	Fat (g)	Protein (g)
Hard-boiled eggs: 100 g	0.1	10.5	12.1
Butter: 20 g	0	16.0	0.14
Bacon: 20 g	0	3.8	3.82
Toast: 50 g	24.35	1.4	4.95
Fries: 115 g	28.06	6.79	2.99
Cooking Oil: 9.2 g (10 ml)	0.01	9.98	0
Milk (Yili Group): 240 ml	12	8.64	7.44
Total (g/kcal)	64.52/258.08	57.11/513.99	31.44/125.76
Total calories	897.83 kcal		

Note: 1 g protein can produce 4000 calories

1 g fat can produce 9000 calories

1 g carbohydrate can produce 4000 calories

Calories of hard-boiled eggs:http://www.boohee.com/shiwu/jidan_zhu

Calories of cooking oil:http://www.boohee.com/shiwu/selayou

Appendix 8: Known CYP Liver Enzyme Activity Inhibitors and Inducers

I. CYP Inhibitors:

1) Strong inhibitors (AUC increase ≥ 5-fold)

atazanavir, telithromycin, clarithromycin, itraconazole, ketoconazole, indinavir, nelfinavir, ritonavir, saquinavir, nafazodone

2) Medium inhibitors (AUC increase ≥2-fold and ≤ 5-fold)

diltiazem, erythromycin, fluconazole, verapamil, ciprofloxacin, grapefruit juice

3) Weak inhibitors (AUC increase ≥1.25-fold and ≤ 2-fold)

cimetidine

ciprofloxacin and erythromycin

Ⅱ. CYP Inducers:

barbital, phenytoin, carbamazepine, rifampin, dexamethasone, steroids, troglitazone, ethanol
